# The Aza-Prins Reaction of 1,2-Dicarbonyl Compounds with 3-Vinyltetrahydroquinolines: Application to the Synthesis of Polycyclic Spirooxindole Derivatives

**DOI:** 10.1021/acs.joc.1c01785

**Published:** 2021-11-18

**Authors:** Shinichi Saito, Tomohiro Katamura, Rei Tsukazaki, Akito Fujisawa, Yusuke Yoshigoe, Yuichiro Mutoh

**Affiliations:** Department of Chemistry, Faculty of Science, Tokyo University of Science, 1-3 Kagurazaka, Shinjuku, Tokyo 162-8601, Japan

## Abstract

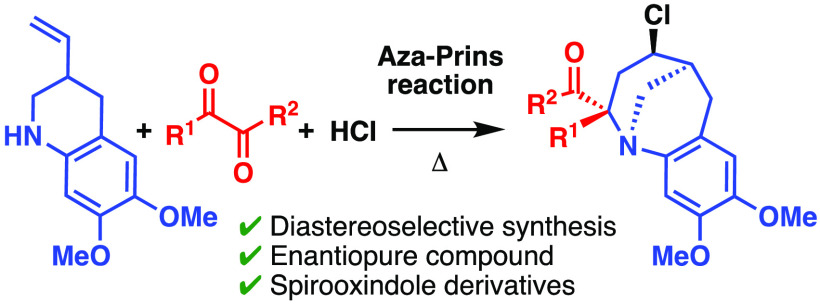

The aza-Prins reaction of 6,7-dimethoxy-3-vinyl-1,2,3,4-tetrahydroquinoline (**1**) with 1,2-dicarbonyl compounds proceeded smoothly in the presence of HCl, and the corresponding tricyclic benzazocines were isolated in yields of 20–86%. The reaction proceeded in a stereoselective manner, and the formation of the 2,4-*trans* isomer was observed. The reaction of **1** with an enantiopure ketoester gave the corresponding tricyclic benzazocine as a mixture of diastereomers. The diastereomers were easily separated and converted to enantiopure tricyclic benzazocines. The synthesis of spirooxindole derivatives was achieved by the reaction of **1** with isatin derivatives.

## Introduction

**Scheme 1:**
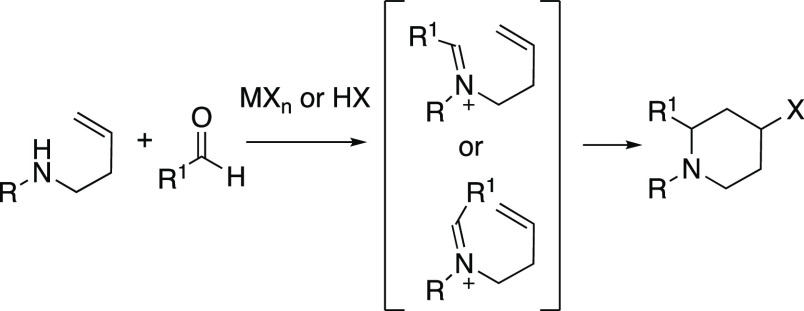
Aza-Prins Reaction

The aza-Prins reaction is a cyclization reaction of an *N*-homoallyliminium ion, which was frequently prepared by the reaction of a homoallylamine with an aldehyde under acidic conditions (Scheme 1).^1^ The importance and usefulness of the aza-Prins reaction have been demonstrated by the application of this reaction to the synthesis of a number of N-heterocyclic natural products and related compounds.^2^ In many examples, an aldehyde was used as the substrate, and other carbonyl compounds such as 1,2-dicarbonyl compounds have been occasionally employed as the substrates.^3,4^

The control of the stereochemistry in the aza-Prins reaction has been recently studied by several groups. Maruoka and Kano reported the asymmetric aza-Prins-type cyclization in the presence of chiral phosphoric acid,^5^ and Dobbs reported the stereoselective aza-Prins reaction by introducing a chiral auxiliary to the homoallylamine.^2c,6^ The enantiopure nitrogen heterocycles synthesized by these studies are expected to be important intermediates for the synthesis of biologically active molecules.

**Scheme 2:**
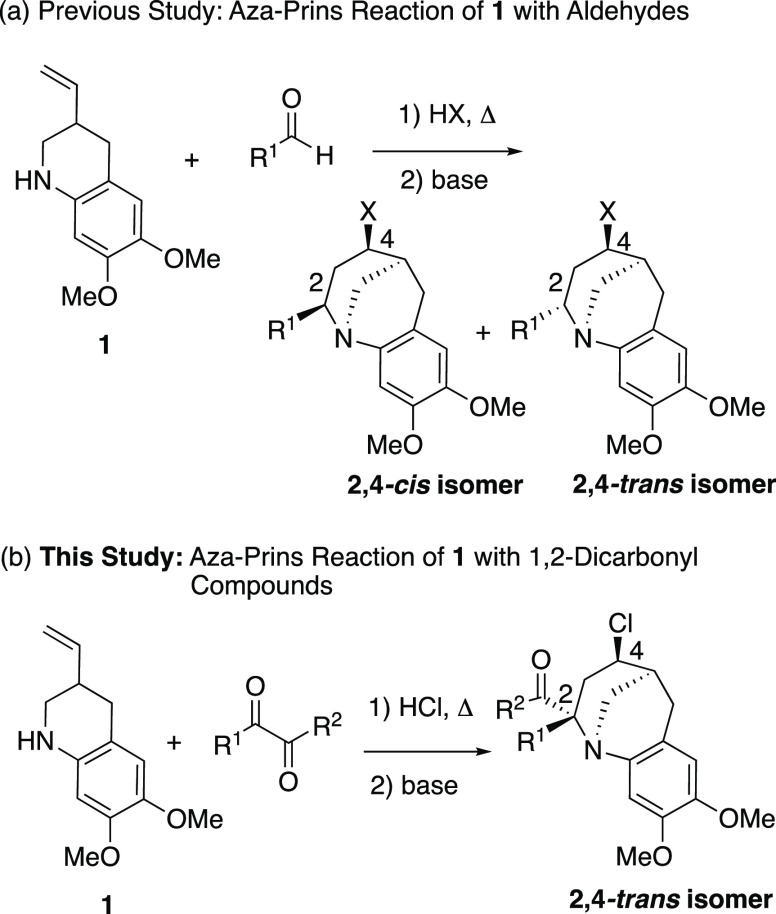
Aza-Prins Reaction of 6,7-Dimethoxy-3-vinyl-1,2,3,4-tetrahydroquinoline (**1**)

Recently we reported the aza-Prins reaction of 2-vinyltetrahydroquinolines with aldehydes (Scheme 2a).^7^ The reaction proceeded in the presence of hydrogen halides, and tricyclic benzazocines were isolated as a mixture of 2,4-*cis-* and 2,4-*trans-*isomers in good to high yields under mild conditions. We envisioned that we could significantly expand the scope of the aza-Prins reaction by introducing 1,2-dicarbonyl compounds as the substrates for this reaction. In this work, we report the aza-Prins reaction of 6,7-dimethoxy-3-vinyl-1,2,3,4-tetrahydroquinoline (**1**) with 1,2-dicarbonyl compounds (Scheme 2b). An enantiopure tricyclic benzazocine was synthesized from **1** and an enantiopure ketoester. The synthesis of spirooxindoles was realized by the reaction of **1** with isatin derivatives.

## Results and Discussion

### Aza-Prins Reaction of a Vinyltetrahydroquinoline with 1,2-Dicarbonyl Compounds

The aza-Prins reaction of **1** with 1,2-dicarbonyl compounds was studied by employing reaction conditions previously reported for the reaction of **1** with aldehydes,^7^ and the results are summarized in Table 1.

**Table 1:**
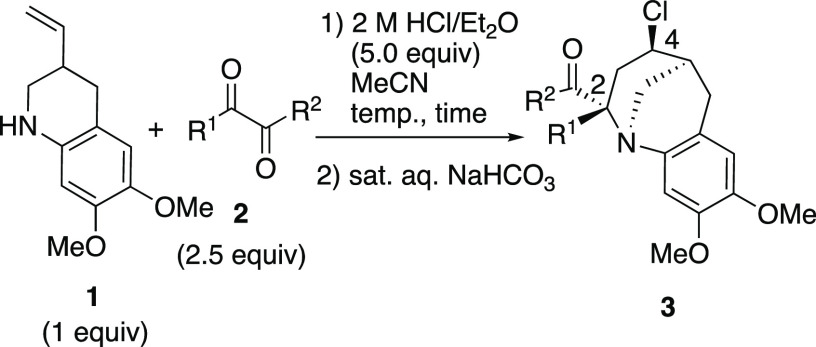

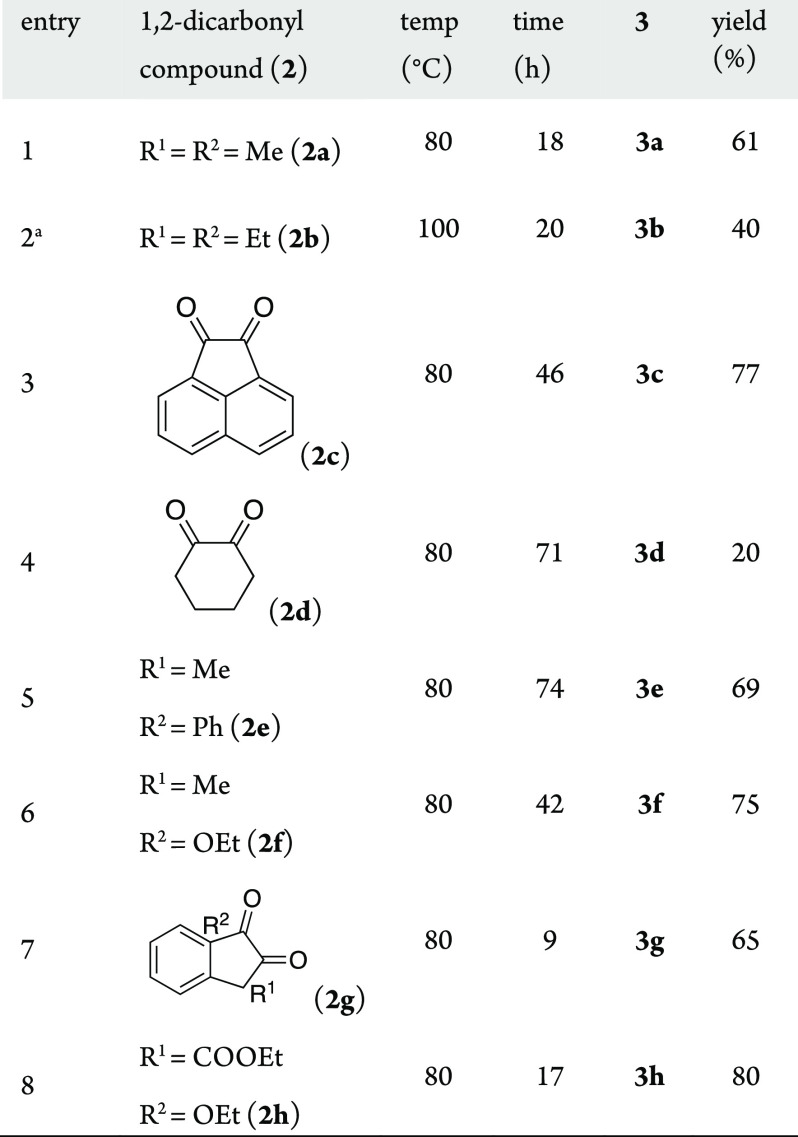
Aza-Prins Reaction of **1** with 1,2-Dicarbonyl Compounds

a A 4 M solution of HCl in dioxane was used.

A mixture of **1**(7) (1.0 equiv), butane-2,3-dione (**2a**, 2.5 equiv), and 2 M HCl (5.0 equiv) in diethyl ether was heated in acetonitrile at 80 °C for 18 h, and the tricyclic benzazocine **3a** was isolated in 61% yield (entry 1). In contrast to the aza-Prins reaction of **1** with aldehydes, where a mixture of diastereomers was isolated, this reaction proceeded in a selective manner. The 2,4-*trans* isomer was isolated as the major product, and the formation of a trace amount of the presumed diastereomer (2,4-*cis* isomer) was occasionally observed. The yield of the product decreased when hexane-3,4-dione (**2b**) was employed as the substrate (entry 2). The reaction of acenaphthoquinone (**2c**) was completed under similar conditions and gave the corresponding polycyclic benzazocine **3c** in 77% yield (entry 3). Though we expected that the reaction of 1,2-cyclohexanedione (**2d**) would proceed smoothly, the yield of the product was low (20%, entry 4).

**Figure 1:**
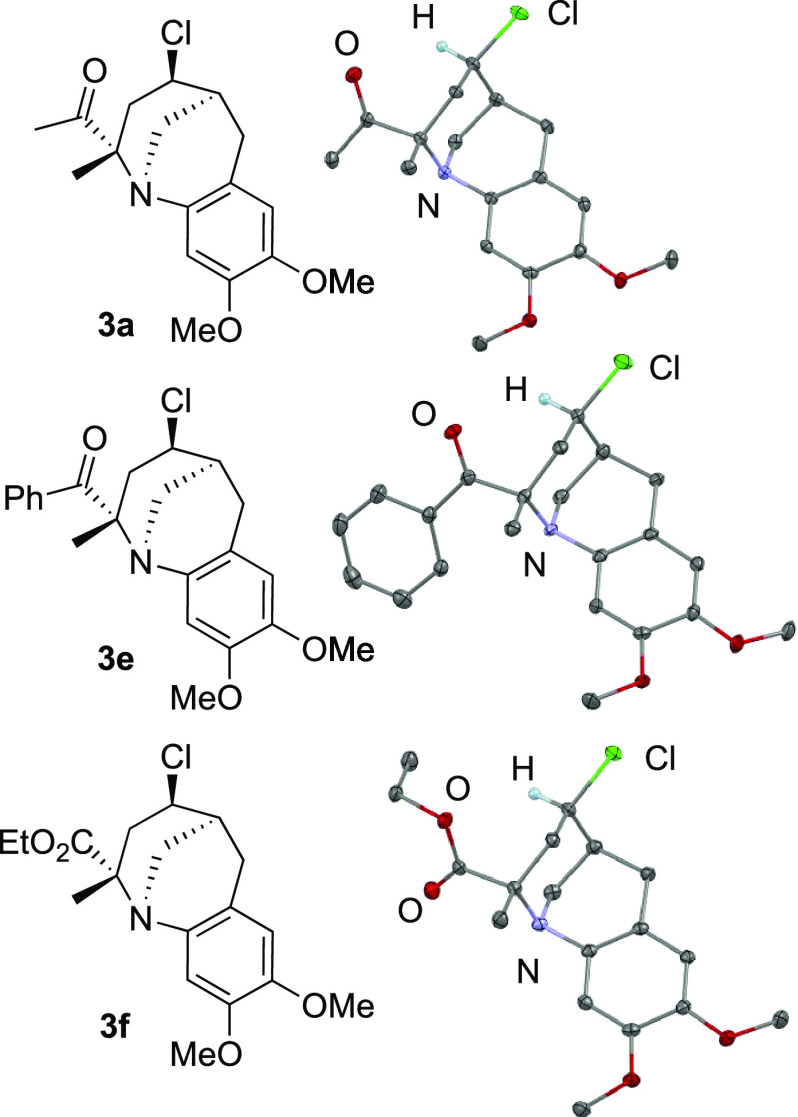
Molecular structures of **3a**, **3e**, and **3f** with thermal ellipsoids at 50% probability.

We next turned our attention to the reaction of unsymmetrically substituted 1,2-dicarbonyl compounds. When 1-phenylpropane-1,2-dione (**2e**) was employed as the substrate, a longer reaction time (74 h) was required for the completion of the reaction, and the product was isolated in 69% yield (entry 5). Only the acetyl group reacted, and the benzoyl group was inert. To expand the scope of this reaction, we examined the reaction of **1** with an α-ketoester. Gratifyingly, ethyl 2-oxopropanoate (**2f**) reacted with **1** and gave the tricyclic compound **3f** in 75% yield (entry 6). Again, the acetyl group reacted preferentially. In the reaction of 1,2-indandione (**2g**), the 2-oxo group was reactive and gave the product in 65% yield (entry 7). Finally, the reaction of a tricarbonyl compound was examined. The reaction of 1,3-diethyl 2-oxopropanedioate (**2h**) proceeded smoothly. The 2-oxo group reacted preferentially, and the corresponding benzazocine was isolated in 80% yield (entry 8).^8^ The molecular structures of **3a**, **3e**, and **3f** were determined by X-ray crystallographic analyses (Figure 1). As shown in Figure 1, the formation of the 2,4-*trans* isomer was confirmed when **1** reacted with diketones (**2a** and **2e**) and a ketoester (**2f**). The results are in sharp contrast to the results of the reaction of **1** with aldehydes, where the formation of a mixture of diastereomers (*cis* and *trans* isomers) with varying ratios was observed.^7^

**Scheme 3:**
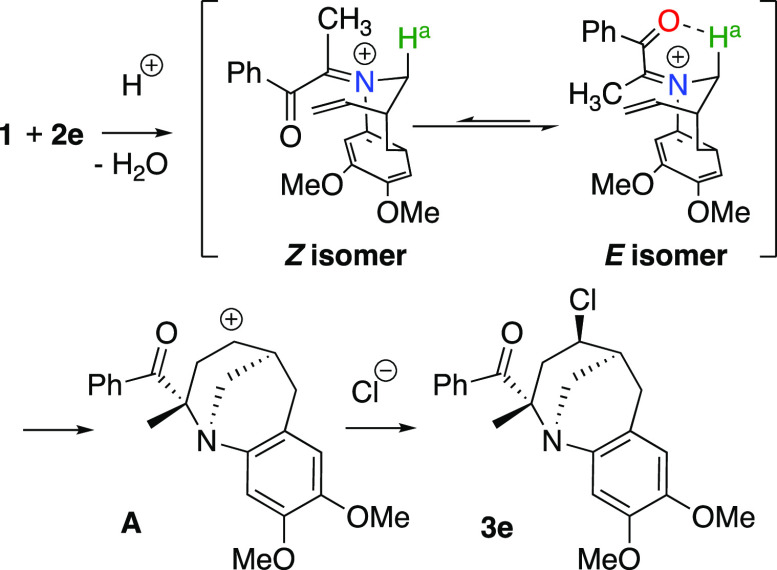
Proposed Mechanism for the Aza-Prins Reaction of **3e**

The observed selectivity of the reaction could be explained by considering the reactivity of the carbonyl group and the stability of the iminium ion, which was formed as the intermediate (Scheme 3). Thus, the acetyl group is more reactive than the benzoyl group (in **2e**) or ethoxycarbonyl group (in **2f**). The amino group of **1** would react preferentially with the acetyl group of **2e**, for example, and the corresponding iminium ion would be formed. Though two isomeric iminium intermediates, *E* isomer and *Z* isomer, would be generated, we assume that the *E* isomer would be preferentially formed. The *E* isomer would be stabilized by the formation of the intramolecular hydrogen bond between the oxygen atom of the carbonyl group and the acidic hydrogen atom (H^a^) of the methylene group bound to the iminium ion. The increased steric hindrance between the *N*-aryl group and the benzoyl group in the *Z* isomer may also contribute to the preferred formation of the *E* isomer. Carbocation **A** would be generated by the cyclization of the *E* isomer, and the chloride ion would attack **A** to provide **3e** as the final product. The attack of the chloride ion will proceed as shown in Scheme 3 because the presence of the bridging methylene group and the acyl group would prevent the formation of the 2,4-*cis* isomer.^7^

**Scheme 4:**
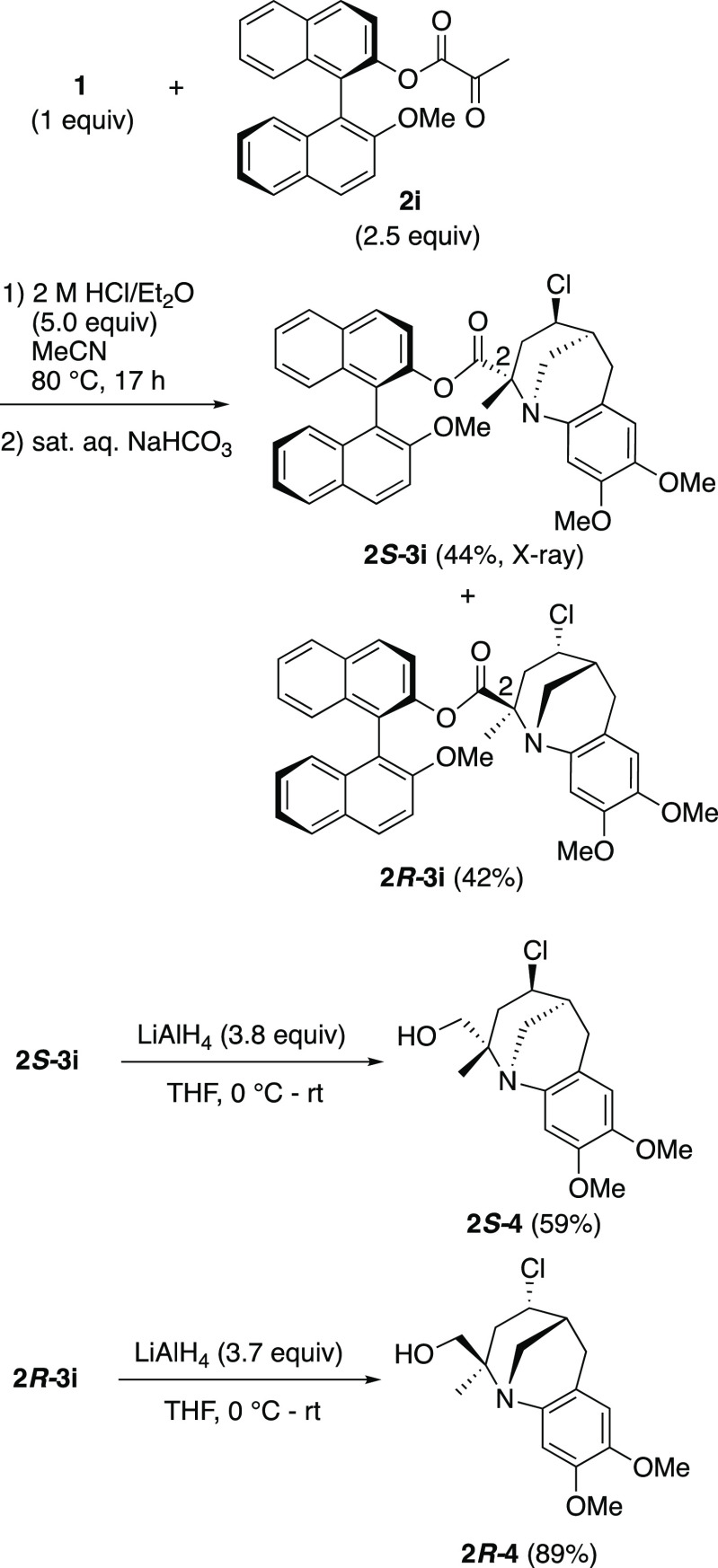
Synthesis of an Enantiopure Benzazocine

The high reactivity of the α-ketoester was applied to the synthesis of an enantiopure tricyclic benzazocine (Scheme 4). Thus, the reaction of **1** with (*R*)-BINOL-derived ketoester **2i** gave the corresponding tricyclic benzazocine as a mixture of diastereomers (**2***S***-3i** and **2***R***-3i**) in 86% combined yield. The molecular structure of **2***S***-3i** was confirmed by X-ray crystallographic analysis (Figure S1). Though essentially no diastereoselectivity was observed for this reaction, the diastereomers were easily separated by silica gel column chromatography. Enantiopure benzazocine **2***S***-4** (or **2***R***-4**) was synthesized by the removal of the chiral auxiliary by the reduction of **2***S***-3g** (or **2***R***-3g**) with LiAlH_4_. The high optical purity (>99% ee) of the products was confirmed by chiral HPLC analysis.^9^

### Synthesis of Spirooxindole Derivatives by the Aza-Prins Reaction of a Vinyltetrahydroquinoline with Isatin Derivatives

A spirooxindole skeleton is incorporated in a large number of natural products, and some derivatives exhibit interesting biological activities such as antitumor, anti-HIV, and antimalarial activities.^10^ Accordingly, the development of a new synthetic method for spirooxindole derivatives is an important issue. On the basis of the observed wide scope of the aza-Prins reaction of **1** with various 1,2-dicarbonyl compounds, we envisioned that polycyclic oxindole derivatives could be synthesized by the aza-Prins reaction of **1** with isatin derivatives.

Compound **1** reacted with isatin (**5a**) at 100 °C for 22 h under standard reaction conditions, and spirooxindole derivative **6a** was isolated in 70% yield (Table 2, entry 1). Again, the reaction proceeded with high diastereoselectivity, and only the *trans* isomer was isolated. The reactivity of 5-nitroisatin (**5b**) was higher than that of **5a**: the reaction was completed in 13 h, and the product (**6b**) was isolated in 69% yield (entry 2). The reactions of other 5-substituted isatin derivatives with electron-withdrawing groups gave the corresponding spirooxindoles in 63–74% yields (entries 3–5). The progress of the reaction of 5-methoxyisatin (**6f**) was slow, and the product was isolated in 33% yield after prolonged heating of the reaction mixture (40 h, entry 6). We also introduced substituents to other positions to the isatin structure and examined the reactivity. Though the reactivity of *N*-methylisatin was low, the reaction was completed in 40 h, and the product was isolated in 82% yield (entry 7). The reactivity of 6- and 7-chloroisatin was comparable to that of **5a**, and the corresponding benzazocines were isolated in moderate yields (entries 8 and 9). The reaction of 4-chloroisatin, however, did not proceed (entry 10). The presence of a large chlorine atom in the proximity of the carbonyl group might inhibit the formation of the corresponding iminium ion, which is the key intermediate of the reaction. The substituent effect on the reaction was briefly screened by reacting two 6-substituted isatins. The reaction of 6-trifluoromethylisatin (**5k**) with **1** was completed in 17 h, and the corresponding benzazocine was isolated in 66% yield (entry 11). In contrast, the reaction of 6-methoxyisatin (**5l**) was sluggish; the formation of unidentified byproducts was observed, and the yield of the corresponding benzazocine was low (3.4% yield, entry 12). The result implies that the facile formation and/or the high reactivity of the iminium ion intermediate would be important for the progress of the reaction.

**Table 2:**
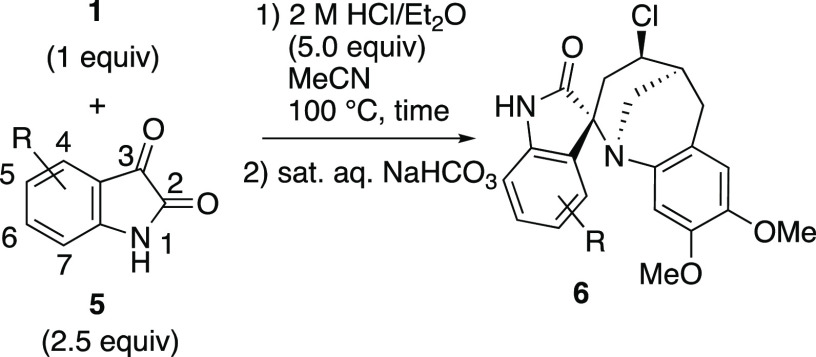
Aza-Prins Reaction of **1** with Isatin Derivatives

entry	isatin	time (h)	product	yield (%)
1	R = H (**5a**)	22	**6a**	70
2	R = 5-NO_2_ (**5b**)	13	**6b**	69
3	R = 5-CF_3_O (**5c**)	8	**6c**	63
4	R = 5-F (**5d**)	46	**6d**	67
5	R = 5-Br (**5e**)	16	**6e**	74
6	R = 5-MeO (**5f**)	40	**6f**	33
7	R = 1-Me (**5g**)	40	**6g**	82
8^a^	R = 6-Cl (**5h**)	22	**6h**	50
9	R = 7-Cl (**5i**)	19	**6i**	69
10^a^	R = 4-Cl (**5j**)	72	**6j**	0
11	R = 6-CF_3_ (**5k**)	17	**6k**	66
12	R = 6-CH_3_O (**5l**)	43	**6l**	3.4

a 4 M HCl/dioxane was used as the acid.

**Figure 2:**
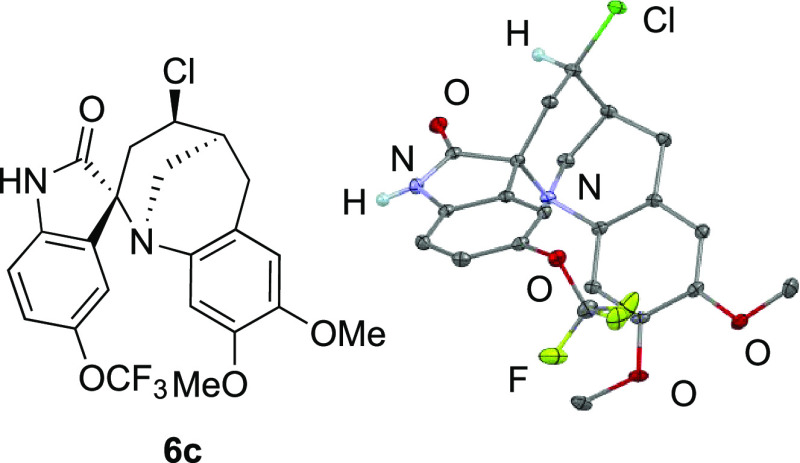
Molecular structure of **6c** with thermal ellipsoids at 50% probability.

The formation of the *trans* isomer was confirmed by an X-ray crystallographic analysis of **6c** (Figure 2). The observed selectivity of the reaction is in accordance with the results of the reactions of α-ketoesters (Scheme 3). The more reactive carbonyl group (C-3 position of the isatin moiety) reacted with the amino group, and the *E* isomer of the iminium salt would be favored because of the presence of the intramolecular hydrogen bond and/or the steric effect. It is noteworthy that the diastereoselectivity of the reaction could be controlled by the use of 1,2-dicarbonyl compounds instead of aldehydes for the aza-Prins reaction; the *trans* isomer could be selectively synthesized regardless of the structure of the dicarbonyl compounds.

## Conclusions

In summary, we developed the aza-Prins reaction of a 3-vinyl-1,2,3,4-tetrahydroquinoline with 1,2-dicarbonyl compounds. The reaction gave tricyclic benzazocines with high chemo- and diastereoselectivity. A BINOL-derived homochiral ketoester was applied to the synthesis of an enantiopure tricyclic benzazocine. The aza-Prins reaction of a 3-vinyl-1,2,3,4-tetrahydroquinoline with isatin derivatives proceeded smoothly, and spirooxindoles incorporating tricyclic benzazocine skeletons were synthesized. The study provides new methods for the synthesis of the benzazocine derivatives with defined stereocenters.

## Experimental Section

Compound **1** was synthesized according to the literature.^7^ Compounds **2a**–**h** and reagents were commercially available and used without further purification unless otherwise noted. An oil bath was used as the heat source. ^1^H and ^13^C{^1^H} NMR spectra were recorded on a 400 or 500 MHz NMR spectrometer. Chemical shifts were reported in delta units (δ) relative to residual chloroform (7.24 ppm for ^1^H NMR) or chloroform-*d* (77.0 ppm for ^13^C NMR) as the internal standard. Coupling constants, *J*, are reported in hertz (Hz). Infrared (IR) spectra were recorded on an FT-IR spectrometer using a diamond ATR module. High-resolution mass spectra were recorded on a quadrupole time-of-flight (TOF) mass spectrometer. Thin-layer chromatography (TLC) was performed on a Merck silica gel 60F_254_ plate. Column chromatography was performed using Kanto Chemical silica gel 60 N (spherical, neutral, 40–50 μm), Kanto Chemical silica gel 60 (spherical, acidic, 40–50 μm, described as “acidic silica gel”), or aluminum oxide 90 active neutral (activity stage I, 63–200 μm, Merck).

### General Procedure for the Synthesis of Tricyclic Benzazocines **3a**–**h** (Procedure A)

A mixture of **1** (0.10 mmol, 1.0 equiv), 1,2-dicarbonyl compound **2** (0.25 mmol, 2.5 equiv), and 2 M HCl in Et_2_O (0.50 mmol, 5.0 equiv) in MeCN (0.2 mL) was heated in a screw-capped vial. To the reaction mixture was added saturated aqueous NaHCO_3_ at rt. The resulting mixture was extracted with EtOAc, and the combined organic layer was washed with brine, dried over Na_2_SO_4_, filtered, and concentrated under reduced pressure. The residue was purified by flash column chromatography on silica gel to afford tricyclic benzazocine **3**.

#### 1-((1S*,2S*,4R*,5S*)-4-Chloro-8,9-dimethoxy-2-methyl-3,4,5,6-tetrahydro-2*H*-1,5-methanobenzo[*b*]azocin-2-yl)ethan-1-one (**3a**)

Procedure A was generally followed to synthesize **3a** from **1** (22 mg, 0.10 mmol, 1.0 equiv) and **2a** (22 μL, 0.25 mmol, 2.5 equiv). The mixture was heated at 80 °C for 18 h. The residue was purified by flash column chromatography on silica gel (hexane/EtOAc, 8:1) to afford **3a** (20 mg, 0.061 mmol, 61%) as a colorless solid. The single crystal for X-ray crystallographic analysis was obtained by recrystallization of **3a** from hexane/acetone: mp 183.2–184.2 °C; ^1^H NMR (400 MHz, CDCl_3_) δ 6.62 (s, 1H), 6.49 (s, 1H), 4.40 (dt, *J* = 12.4, 4.4 Hz, 1H), 3.85 (s, 3H), 3.84 (s, 3H), 3.14 (m, 2H), 2.79 (m, 2H), 2.37 (m, 4H), 2.24 (br s, 1H), 1.24 (t, *J* = 13.2 Hz, 1H), 1.06 (s, 3H); ^13^C{^1^H} NMR (100.3 MHz, CDCl_3_) δ 212.0, 147.0, 146.0, 136.5, 125.5, 112.3, 111.0, 72.2, 60.4, 56.0, 55.9, 51.6, 34.4, 33.0, 26.1, 25.7, 23.6; IR (ATR) 1703 cm^–1^; HRMS (ESI-TOF) calcd for C_17_H_23_NO_3_Cl [M + H]^+^ 324.1361, found 324.1358.

#### 1-((1S*,2S*,4R*,5S*)-4-Chloro-2-ethyl-8,9-dimethoxy-3,4,5,6-tetrahydro-2*H*-1,5-methanobenzo[*b*]azocin-2-yl)propan-1-one (**3b**)

Procedure A was generally followed to synthesize **3b** from **1** (22 mg, 0.10 mmol, 1.0 equiv) and **2b** (30 μL, 0.25 mmol, 2.5 equiv). Four M HCl in dioxane (0.13 mL, 0.50 mmol, 5.0 equiv) was used instead of 2 M HCl in ether. The mixture was heated at 100 °C for 20 h. The residue was purified by flash column chromatography on silica gel (hexane/EtOAc, 5:1) to afford **3b** (14 mg, 0.040 mmol, 40%) as a pale yellow viscous oil: ^1^H NMR (400 MHz, CDCl_3_) δ 6.61 (s, 1H), 6.52 (s, 1H), 4.46 (dt, *J* = 12.4, 4.4 Hz, 1H), 3.84 (s, 6H), 3.10 (m, 2H), 2.98 (dq, *J* = 17.6, 7.2 Hz, 1H), 2.80 (dd, *J* = 18.2, 8.4 Hz, 1H), 2.64 (m, 2H), 2.51 (dd, *J* = 13.0, 4.8 Hz, 1H), 2.26 (br s, 1H), 1.85 (dq, *J* = 13.6, 7.6 Hz, 1H), 1.19 (dq, *J* = 13.8, 8.0 Hz, 1H), 1.09 (m, 4H), 0.59 (t, *J* = 8.0 Hz, 3H); ^13^C{^1^H} NMR (100.3 MHz, CDCl_3_) δ 213.8, 147.0, 146.0, 137.0, 125.7, 112.7, 111.0, 75.6, 61.0, 56.1, 55.9, 51.8, 33.6, 32.2, 32.1, 28.7, 25.9, 8.3, 8.2; IR (ATR) 1706 cm^–1^; HRMS (ESI-TOF) calcd for C_19_H_27_NO_3_Cl [M + H]^+^ 352.1674, found 352.1667.

#### (1R*,1′S*,4′R*,5′S*)-4′-Chloro-8′,9′-dimethoxy-3′,4′,5′,6′-tetrahydro-2*H*-spiro[acenaphthylene-1,2′-[1,5]methanobenzo[*b*]azocin]-2-one (**3c**)

Procedure A was generally followed to synthesize **3c** from **1** (22 mg, 0.10 mmol, 1.0 equiv) and **2c** (46 mg, 0.25 mmol, 2.5 equiv). The mixture was heated at 80 °C for 46 h. The residue was purified by flash column chromatography on silica gel (hexane/EtOAc, 6:1) to afford **3c** (32 mg, 0.077 mmol, 77%) as a pale yellow solid: mp 186.5–189.4 °C; ^1^H NMR (400 MHz, CDCl_3_) δ 8.10 (d, *J* = 8.0 Hz, 1H), 8.00 (d, *J* = 6.8 Hz, 1H), 7.83 (d, *J* = 8.8 Hz, 1H), 7.74 (t, *J* = 7.8 Hz, 1H), 7.37 (t, *J* = 7.8 Hz, 1H), 6.70 (s, 1H), 6.27 (d, *J* = 7.2 Hz, 1H), 5.46 (dt, *J* = 12.4, 4.8 Hz, 1H), 5.28 (s, 1H), 4.34 (d, *J* = 14.0 Hz, 1H), 3.90 (s, 3H), 3.37 (d, *J* = 18.4 Hz, 1H), 3.29 (s, 3H), 3.08 (dd, *J* = 13.7, 2.7 Hz, 1H), 2.99 (dd, *J* = 18.3, 8.7 Hz, 1H), 2.62 (br s, 1H), 2.32 (t, *J* = 13.2 Hz, 1H), 2.08 (dd, *J* = 13.8, 5.2 Hz, 1H); ^13^C{^1^H} NMR (100.3 MHz, CDCl_3_) δ 201.6, 147.0, 144.8, 141.0, 139.0, 138.2, 131.7, 131.3, 130.5, 128.4, 127.1, 126.3, 125.3, 124.3, 122.9, 114.1, 110.5, 71.2, 59.9, 55.9, 55.3, 48.4, 33.8, 33.0, 25.5; IR (ATR) 1715 cm^–1^; HRMS (ESI-TOF) calcd for C_25_H_23_NO_3_Cl [M + H]^+^ 420.1361, found 420.1361.

#### (1S*,1′S*,4′R*,5′S*)-4′-Chloro-8′,9′-dimethoxy-3′,4′,5′,6′-tetrahydrospiro[cyclohexane-1,2′-[1,5]methanobenzo[*b*]azocin]-2-one (**3d**)

Procedure A was generally followed to synthesize **3d** from **1** (22 mg, 0.10 mmol, 1.0 equiv) and **2d** (28 mg, 0.25 mmol, 2.5 equiv). The mixture was heated at 80 °C for 71 h. The residue was purified by flash column chromatography on silica gel (hexane/EtOAc, 5:1) to afford **3d** (7.0 mg, 0.020 mmol, 20%) as a pale yellow solid: mp 152.6–153.4 °C; ^1^H NMR (400 MHz, CDCl_3_) δ 6.83 (s, 1H), 6.62 (s, 1H), 4.85 (dt, *J* = 12.4, 4.8 Hz, 1H), 3.85 (s, 3H), 3.82 (s, 3H), 3.27–3.16 (m, 2H), 3.09 (dd, *J* = 14.1, 1.8 Hz, 1H), 2.79 (m, 2H), 2.49–2.39 (m, 1H), 2.27–2.19 (m, 3H), 2.06 (dd, *J* = 12.8, 4.8 Hz, 1H), 1.87 (dd, *J* = 15.1, 1.8 Hz, 1H),, 1.77–1.62 (m, 2H), 1.30–1.19 (m, 2H); ^13^C{^1^H} NMR (100.3 MHz, CDCl_3_) δ 215.2, 146.8, 146.1, 136.4, 126.0, 111.3, 110.8, 73.5, 60.2, 55.9, 51.0, 40.3, 38.7, 35.2, 33.3, 30.4, 29.7, 26.4, 21.9; IR (ATR) 1705 cm^–1^; HRMS (ESI-TOF) calcd for C_19_H_25_NO_3_Cl [M + H]^+^ 350.1517, found 350.1515.

#### ((1S*,2*S*,*4R*,5S*)-4-Chloro-8,9-dimethoxy-2-methyl-3,4,5,6-tetrahydro-2*H*-1,5-methanobenzo[*b*]azocin-2-yl)(phenyl)methanone (**3e**)

Procedure A was generally followed to synthesize **2e** from **1** (22 mg, 0.10 mmol, 1.0 equiv) and **3e** (37 mg, 0.25 mmol, 2.5 equiv). The mixture was heated at 80 °C for 74 h. The residue was purified by flash column chromatography on silica gel (hexane/EtOAc, 12:1) to afford **3e** (27 mg, 0.069 mmol, 69%) as a colorless solid. The single crystal for X-ray crystallographic analysis was obtained by recrystallization of **3e** from hexane/acetone: mp 210.4–211.3 °C; ^1^H NMR (400 MHz, CDCl_3_) δ 8.65 (d, *J* = 7.2 Hz, 2H), 7.56 (t, *J* = 7.2 Hz, 1H), 7.45 (t, *J* = 8.0 Hz, 2H), 6.66 (s, 1H), 6.65 (s, 1H), 4.60 (dt, *J* = 12.4, 4.4 Hz, 1H), 3.88 (s, 3H), 3.86 (s, 3H), 3.19 (d, *J* = 18.4 Hz, 1H), 3.07 (dd, *J* = 14.0, 2.4 Hz, 1H), 2.84–2.80 (m, 2H), 2.55 (dd, *J* = 13.2, 4.4 Hz, 1H), 2.26 (br s, 1H), 1.39 (t, *J* = 12.8 Hz, 1H), 1.33 (s, 3H); ^13^C{^1^H} NMR (100.3 MHz, CDCl_3_) δ 201.8, 147.0, 146.1, 136.6, 135.3, 132.9, 130.3, 128.2, 125.8, 112.1, 111.3, 73.0, 60.7, 56.2, 55.9, 51.6, 36.5, 33.1, 27.4, 26.1; IR (ATR) 1668 cm^–1^; HRMS (ESI-TOF) calcd for C_22_H_25_NO_3_Cl [M + H]^+^ 386.1518, found 386.1512.

#### (1S*,2S*,4R*,5S*)-4-Chloro-8,9-dimethoxy-2-methyl-3,4,5,6-tetrahydro-2*H*-1,5-methanobenzo[*b*]azocine-2-carboxylate (**3f**)

Procedure A was generally followed to synthesize **3f** from **1** (44 mg, 0.20 mmol, 1.0 equiv) and **2f** (55 μL, 0.50 mmol, 2.5 equiv). The mixture was heated at 80 °C for 42 h. The residue was purified by flash column chromatography on silica gel (hexane/EtOAc, 2:1) to afford **3f** (53 mg, 0.15 mmol, 75%) as a colorless solid: mp 132.7–134.7 °C; ^1^H NMR (400 MHz, CDCl_3_) δ 6.60 (s, 1H), 6.55 (s, 1H), 4.48 (dt, *J* = 12.4, 4.0 Hz, 1H), 4.32 (m, 2H), 3.84 (s, 3H), 3.80 (s, 3H), 3.20–3.11 (m, 2H), 3.06 (d, *J* = 14.1 Hz, 1H), 2.84 (dd, *J* = 18.6, 8.4 Hz, 1H), 2.37 (dd, *J* = 13.4, 4.0 Hz, 1H), 2.28 (br s, 1H), 1.41 (t, *J* = 13.2 Hz, 1H), 1.19 (t, *J* = 6.8 Hz, 3H), 1.23 (s, 3H); ^13^C{^1^H} NMR (100.3 MHz, CDCl_3_) δ 174.3, 146.9, 145.9, 136.3, 125.5, 112.8, 110.8, 67.7, 61.6, 60.8, 55.9, 55.9, 51.2, 36.3, 33.0, 27.4, 26.0, 14.3; IR (ATR) 1740 cm^–1^; HRMS (ESI-TOF) calcd for C_18_H_25_NO_4_Cl [M + H]^+^ 354.1467, found 354.1467.

#### (1′S*,2S*,4′R*,5′S*)-4′-Chloro-8′,9′-dimethoxy-3′,4′,5′,6′-tetrahydrospiro[indene-2,2′-[1,5]methanobenzo[*b*]azocin]-1(3H)-one (**3g**)

Procedure A was generally followed to synthesize **3g** from **1** (44 mg, 0.20 mmol, 1.0 equiv) and **2g** (73 mg, 0.50 mmol, 2.5 equiv). The mixture was heated at 80 °C for 9 h. The residue was purified by flash column chromatography on silica gel (hexane/EtOAc, 3:1) to afford **3g** (6.0 mg, 0.13 mmol, 65%) as an off white solid: mp 178.6–179.2 °C; ^1^H NMR (400 MHz, CDCl_3_) δ 7.77 (d, *J* = 7.3 Hz, 1H), 7.58 (t, *J* = 7.5 Hz, 1H), 7.39 (t, *J* = 7.6 Hz, 1H), 7.32 (d, *J* = 7.3 Hz, 1H), 6.65 (s, 1H), 6.38 (s, 1H), 5.17 (dt, *J* = 12.4, 4.4 Hz, 1H), 4.21 (d, *J* = 14.0 Hz, 1H), 3.86 (s, 3H), 3.78 (s, 3H), 3.55 (d, *J* = 16.8 Hz, 1H), 3.21 (d, *J* = 18.0 Hz, 1H), 2.97 (d, *J* = 12.8 Hz, 1H), 2.87 (dd, *J* = 18.3, 8.2 Hz, 1H), 2.53–2.44 (m, 2H), 2.01 (dd, *J* = 13.7, 5.0 Hz, 1H), 1.85 (t, *J* = 13.2 Hz, 1H); ^13^C NMR (126 MHz, CDCl_3_) δ 204.1, 149.4, 147.2, 146.2, 138.4, 135.3, 135.0, 127.9, 126.6, 125.6, 125.2, 112.4, 111.3, 70.8, 60.0, 56.0, 55.9, 48.6, 41.3, 34.9, 33.6, 25.5; IR (ATR) 1704 cm^–1^ ; HRMS (ESI-TOF) calcd for C_22_H_23_ClNO_3_ [M + H]^+^ 384.1361, found 384.1361.

#### Diethyl (1S*,4R*,5S*)-4-chloro-8,9-dimethoxy-3,4,5,6-tetrahydro-2H-1,5-methanobenzo[*b*]azocine-2,2-dicarboxylate (**3h**)

Procedure A was generally followed to synthesize **3h** from **1** (44 mg, 0.20 mmol, 1.0 equiv) and **2h** (87 mg, 0.50 mmol, 2.5 equiv). The mixture was heated at 80 °C for 17 h. The residue was purified by flash column chromatography on silica gel (hexane/EtOAc, 2:1) to afford **3h** (6.0 mg, 0.16 mmol, 80%) as a colorless solid: mp 144.3–145.6 °C; ^1^H NMR (400 MHz, CDCl_3_) δ 6.60 (s, 1H), 6.53 (s, 1H), 4.41–4.17 (m, 4H), 4.11–4.03 (m, 1H), 3.82 (s, 3H), 3.72 (s, 3H), 3.68 (d, *J* = 14.0 Hz, 1H), 3.19–3.14 (m, 2H), 2.86 (dd, *J* = 18.0, 8.4 Hz, 1H), 2.50 (dd, *J* = 13.6, 4.0 Hz, 1H), 2.28–2.21 (m, 2H), 1.30 (t, *J* = 7.1 Hz, 3H), 1.24 (t, *J* = 7.3 Hz, 3H); ^13^C NMR (100 MHz, CDCl_3_) δ 168.2, 166.8, 147.2, 146.7, 137.5, 125.6, 111.0, 110.1, 76.3, 62.4, 62.2, 60.2, 55.8, 55.7, 50.6, 33.4, 31.9, 25.8, 14.0, 13.8; IR (ATR) 1740, 1714 cm^–1^; HRMS (ESI-TOF) calcd for C_20_H_27_ClNO_6_ [M + H]^+^ 412.1521, found 412.1521.

#### (*R*)-2′-Methoxy-[1,1′-binaphthalen]-2-yl 2-oxopropanoate (**2i**)

Methanesulfonyl chloride (2.32 mL, 30 mmol, 3.8 equiv) was added dropwise to a solution of (*R*)-2-hydroxy-2′-methoxy-1,1′-binaphthyl^11^ (2.38 g, 8 mmol, 1.0 equiv), pyridine (3.21 mL, 40 mmol, 5.0 equiv), and pyruvic acid (1.10 mL, 16 mmol, 2.0 equiv) in anhydrous THF (48 mL) at 0 °C under Ar, and the mixture was stirred for 4 h at rt. The mixture was quenched with water and extracted with MTBE. The combined organic layer was dried over sodium sulfate and evaporated. The residue was purified by flash column chromatography on acidic silica gel (hexane/CH_2_Cl_2_ = 1:1) to afford **2i** (2.7 g, 7.2 mmol, 90%) as a pale yellow amorphous solid: ^1^H NMR (400 MHz, CDCl_3_) δ 8.01 (d, *J* = 8.8 Hz, 1H), 7.96 (t, *J* = 8.0 Hz, 2H), 7.83 (d, *J* = 8.4 Hz, 1H), 7.49–7.45 (m, 2H), 7.40 (d, *J* = 8.8 Hz, 1H), 7.33–7.29 (m, 3H), 7.25–7.21 (m, 1H), 7.09 (d, *J* = 8.7 Hz, 1H), 3.75 (s, 3H), 1.84 (s, 3H); ^13^C{^1^H} NMR (100.3 MHz, CDCl_3_) δ 191.1, 158.6, 154.9, 146.0, 133.5, 133.4, 132.1, 130.3, 129.4, 128.8, 128.2, 127.9, 126.8, 126.7, 126.3, 125.9, 125.1, 125.0, 123.8, 120.6, 116.7, 113.4, 56.5, 26.4 ; IR (ATR) 1737 cm^–1^; HRMS (ESI-TOF) calcd for C_24_H_19_O_4_ [M + H]^+^ 371.1278, found 371.1278; [α]_D_^24^ −23.9 (*c* 1.00, THF).

### (*R*)-2′-Methoxy-[1,1′-binaphthalen]-2-yl-(1*S*,2*S*,4*R*,5*S*)-4-chloro-8,9-dimethoxy-2-methyl-3,4,5,6-tetrahydro-2*H*-1,5-methanobenzo[*b*]azocine-2-carboxylate (**2***S***-3i**) and (*R*)-2′-Methoxy-[1,1′-binaphthalen]-2-yl-(1*R*,2*R*,4*S*,5*R*)-4-chloro-8,9-dimethoxy-2-methyl-3,4,5,6-tetrahydro-2*H*-1,5-methanobenzo[*b*]azocine-2-carboxylate (**2***R***-3i**)

A mixture of **1** (22 mg, 0.10 mmol, 1.0 equiv), **2i** (93 mg, 0.25 mmol, 2.5 equiv), and 2 M HCl in Et_2_O (0.25 mL, 0.50 mmol, 5.0 equiv) in MeCN (0.20 mL) was stirred in a screw-capped vial at 80 °C for 17 h. To the reaction mixture was added saturated aqueous NaHCO_3_ at rt. The resulting mixture was extracted with EtOAc, and the combined organic layer was washed with brine, dried over Na_2_SO_4_, filtered, and concentrated under reduced pressure. The residue was purified by flash column chromatography on acidic silica gel (hexane/MTBE = 2:1) to afford **2***S***-3i** (27 mg, 0.044 mmol, 44%) and **2***R***-3i** (25 mg, 0.042 mmol, 42%). A single crystal for X-ray crystallographic analysis was obtained by recrystallization of **2***S***-3i** from hexane/CH_2_Cl_2_.

#### **2***S*-**3i**:

colorless powder; mp 123.3–125.6 °C; ^1^H NMR (400 MHz, CDCl_3_) δ 8.01 (d, *J* = 9.2 Hz, 2H), 7.96 (d, *J* = 8.4 Hz, 1H), 7.80 (d, *J* = 8.0 Hz, 1H), 7.52 (d, *J* = 9.2 Hz, 1H), 7.50–7.46 (m, 1H), 7.34–7.23 (m, 6H), 6.47 (s, 1H), 6.41 (s, 1H), 3.83 (s, 3H), 3.78 (s, 3H), 3.74 (s, 3H), 2.89–2.83 (m, 2H), 2.58 (dd, *J* = 18.2, 8.8 Hz, 1H), 2.42 (d, *J* = 14.4 Hz, 1H), 2.09 (dd, *J* = 13.6, 4.8 Hz, 1H), 1.42 (br s, 1H), 1.24–1.15 (m, 2H), 1.09 (s, 3H). ; ^13^C{^1^H} NMR (126 MHz, CDCl_3_) δ 172.8, 155.0, 146.7, 145.8, 136.1, 133.8, 133.6, 131.9, 130.1, 129.4, 128.9, 128.1, 127.5, 127.0, 126.7, 126.0, 125.8, 125.7, 125.4, 124.0, 121.4, 117.6, 114.0, 112.8, 110.6, 67.5, 60.3, 56.8, 55.9, 55.8, 49.8, 36.0, 32.7, 27.4, 25.7 (two signals are missing); IR (ATR) 1759 cm^–1^; HRMS (ESI-TOF) calcd for C_37_H_35_ClNO_5_ [M + H]^+^): 608.2198, found 608.2199; [α]_D_^24^ −61.0 (*c* 1.0, THF).

#### **2***R*-**3i**:

colorless powder; mp 119.1–122.2 °C; ^1^H NMR (400 MHz, CDCl_3_) δ 8.01 (d, *J* = 8.8 Hz, 1H), 7.95 (d, *J* = 8.8 Hz, 2H), 7.83 (d, *J* = 7.2 Hz, 1H), 7.50–7.44 (m, 1H), 7.40–7.27 (m, 6H), 7.21 (d, *J* = 8.4 Hz, 1H), 6.49 (s, 1H), 6.31 (s, 1H), 3.79 (s, 3H), 3.76 (s, 3H), 3.72 (s, 3H), 3.36-31 (m, 1H), 2.92 (d, *J* = 18.4 Hz, 1H), 2.73 (dd, *J* = 13.6, 2.4 Hz, 1H), 2.65 (dd, *J* = 18.6, 8.8 Hz, 1H), 2.22 (d, *J* = 14.0 Hz, 1H), 1.98 (dd, *J* = 13.8, 4.4 Hz, 1H), 1.73 (br s, 1H), 1.11 (t, *J* = 12.8 Hz, 1H), 0.77 (s, 3H).; ^13^C{^1^H} NMR (126 MHz, CDCl_3_) δ 172.7, 155.2, 146.8, 146.7, 145.8, 136.3, 133.7, 131.9, 130.1, 129.4, 128.9, 128.1, 127.7, 126.9, 126.6, 126.0, 125.7, 125.4, 124.0, 121.4, 117.6, 113.6, 112.7, 110.7, 67.5, 60.2, 56.7, 55.9, 55.8, 50.6, 36.0, 32.8, 26.9, 25.7 (two signals are missing); IR (ATR) 1747 cm^–1^; HRMS (ESI-TOF) calcd for C_37_H_35_ClNO_5_ [M + H]^+^ 608.2198, found 608.2198; [α]_D_^24^ +96.5 (*c* 1.0, THF).

### Large-Scale Synthesis of **2***S***-3i** and **2***R***-3i**

A mixture of **1** (221 mg, 1.0 mmol, 1.0 equiv), **2i** (932 mg, 2.5 mmol, 2.5 equiv), and 2 M HCl in Et_2_O (2.5 mL, 5.0 mmol, 5.0 equiv) in MeCN (2.0 mL) was stirred in a screw-capped vial at 80 °C for 17 h. The mixture was worked up and purified as described in the small-scale synthesis to afford **2***S***-3i** (243 mg, 0.40 mmol, 40%) and **2***R***-3i** (223 mg, 0.37 mmol, 37%).

### ((1S*,2S*,4R*,5S*)-4-Chloro-8,9-dimethoxy-2-methyl-3,4,5,6-tetrahydro-2*H*-1,5-methanobenzo[*b*]azocin-2-yl)methanol (*rac***-4**)

The racemic compound was prepared by the reduction of **3f**. To a solution of **3f** (120 mg, 0.34 mmol) in THF (1.8 mL) was slowly added LiAlH_4_ (48 mg, 1.3 mmol, 3.8 equiv) at 0 °C. The resultant mixture was then stirred at 0 °C for 20 min before being allowed to warm to rt, and the mixture was stirred for an additional 3 h. The reaction mixture was cooled to 0 °C, and Na_2_SO_4_·10 H_2_O (529 mg) was added carefully in several portions. THF (1.5 mL) was added during this quench to maintain efficient stirring. The resultant mixture was allowed to warm to rt and stirred for 2 h. The crude reaction mixture was filtered through Celite, and the filtrate was evaporated. The residue was purified by flash column chromatography on silica gel (hexane/EtOAc = 1:2) to afford *rac***-4** (85 mg, 0.27 mmol, 80%) as an off-white solid: mp 157.3–158.8 °C; ^1^H NMR (400 MHz, CDCl_3_) δ 6.60 (s, 1H), 6.41 (s, 1H), 4.48 (dt, *J* = 12.8, 4.8 Hz, 1H), 4.01 (d, *J* = 10.8 Hz, 1H), 3.84 (s, 3H), 3.80 (s, 3H), 3.33 (d, *J* = 14.1 Hz, 1H), 3.23 (t, *J* = 9.8 Hz, 1H), 3.11 (d, *J* = 18.8 Hz, 1H), 3.00 (dd, *J* = 13.6, 2.8 Hz, 1H), 2.92 (br d, *J* = 9.1 Hz, 1H), 2.84 (dd, *J* = 18.4, 8.8 Hz, 1H), 2.38 (br, 1H), 1.67 (dd, *J* = 14.4, 4.8 Hz, 1H), 1.53 (t, *J* = 13.6 Hz, 1H), 1.00 (s, 3H); ^13^C{^1^H} NMR (100.3 MHz, CDCl_3_) δ 147.0, 146.0, 137.9, 125.3, 112.3, 110.8, 64.3, 61.8, 60.5, 55.9, 55.9, 48.1, 36.8, 33.6, 26.7, 25.6 ; IR (ATR) 3328 cm^–1^; HRMS (ESI-TOF) calcd for C_16_H_23_ClNO_3_ [M + H]^+^ 312.1361, found 312.1361.

#### ((1*S*,2*S*,4*R*,5*S*)-4-Chloro-8,9-dimethoxy-2-methyl-3,4,5,6-tetrahydro-2*H*-1,5-methanobenzo[*b*]azocin-2-yl)methanol (**2***S***-4**)

To a solution of **2***S***-3i** (124 mg, 0.20 mmol) in THF (1.1 mL) was slowly added LiAlH_4_ (29 mg, 0.76 mmol, 3.8 equiv) at 0 °C. The resultant mixture was then stirred at 0 °C for 20 min before being warmed to rt and stirred for an additional 2 h. Upon completion, the reaction contents were cooled to 0 °C, and Na_2_SO_4_·10H_2_O (318 mg) was then added carefully in several portions. THF (0.87 mL) was added during this quench to maintain efficient stirring. The resultant mixture was allowed to warm to rt and stirred for 10 min. The crude reaction mixture was filtered through Celite, and the filtrate was evaporated. The residue was purified by flash column chromatography on silica gel (hexane/EtOAc = 1:2) to afford **2***S***-4** (38 mg, 0.12 mmol, 59%) as an off-white solid: mp 147.3–148.4 °C; [α]_D_^24^ −127.9 (*c* 1.0, THF). The ^1^H NMR spectrum was in accordance with the data of *rac***-4**.

#### ((1*R*,2*R*,4*S*,5*R*)-4-Chloro-8,9-dimethoxy-2-methyl-3,4,5,6-tetrahydro-2*H*-1,5-methanobenzo[*b*]azocin-2-yl)methanol (**2***R***-4**)

To a solution of **2***R***-3i** (180 mg, 0.30 mmol) in THF (1.6 mL) was slowly added LiAlH_4_ (42 mg, 1.1 mmol, 3.7 equiv) at 0 °C. The resultant mixture was then stirred at 0 °C for 20 min before being allowed to warm to rt, and the mixture was stirred for an additional 2 h. The reaction mixture was cooled to 0 °C, and Na_2_SO_4_·10 H_2_O (318 mg) was added carefully in several portions. THF (1.2 mL) was added during this quench to maintain efficient stirring. The resultant mixture was allowed to warm to rt and stirred for 10 min. The crude reaction mixture was filtered through Celite, and the filtrate was evaporated. The residue was purified by flash column chromatography on silica gel (hexane/EtOAc = 1:2) to afford **2***R***-4** (82 mg, 0.12 mmol, 89%) as an off-white solid: mp 147.2–148.0 °C; [α]_D_^24^ +122.3 (*c* 1.0, THF). The ^1^H NMR spectrum was in accordance with the data of *rac***-4**.

### General Procedure for the Synthesis of Tricyclic Benzazocine **6a**–**l** (Procedure B)

A mixture of **1** (0.1 mmol, 1.0 equiv), isatin **5** (0.25 mmol, 2.5 equiv), and 2 M HCl in Et_2_O (0.25 mL, 0.50 mmol, 5.0 equiv) in MeCN (0.20 mL) was stirred in a screw-capped vial at 100 °C. To the reaction mixture was added saturated aqueous NaHCO_3_ at rt. The resulting mixture was extracted with EtOAc, and the combined organic layer was washed with brine, dried over Na_2_SO_4_, filtered, and concentrated under reduced pressure. The residue was purified by flash column chromatography on silica gel to afford tricyclic benzazocine **6**.

#### (1′S*,3R*,4′R*,5′S*)-4′-Chloro-8′,9′-dimethoxy-3′,4′,5′,6′-tetrahydrospiro[indoline-3,2′-[1,5]methanobenzo[*b*]azocin]-2-one (**6a**)

Procedure B was generally followed to synthesize **6a** from **1** (22 mg, 0.1 mmol, 1.0 equiv) and **5a** (37 mg, 0.25 mmol, 2.5 equiv). The residue was purified by flash column chromatography on silica gel (hexane/EtOAc, 3:2–1:1) to afford **6a** (27 mg, 0.070 mmol, 70%) as a colorless solid: mp 206.6–207.6 °C; ^1^H NMR (400 MHz, CDCl_3_) δ 7.42 (br s, 1H), 7.19 (td, *J* = 7.8, 1.6 Hz, 1H), 6.83 (d, *J* = 8.0 Hz, 1H), 6.74 (td, *J* = 7.4, 1.2 Hz, 1H), 6.68 (s, 1H), 5.92 (d, *J* = 7.2 Hz, 1H), 5.62 (s, 1H), 5.30 (dt, *J* = 12.4, 4.4 Hz, 1H), 4.50 (dd, *J* = 13.6, 1.2 Hz, 1H), 3.88 (s, 3H), 3.41 (s, 3H), 3.29 (d, *J* = 18.8 Hz, 1H), 3.01 (dd, *J* = 13.7, 2.7 Hz, 1H), 2.95 (dd, *J* = 18.5, 8.4 Hz, 1H), 2.53 (br s, 1H), 2.08 (t, *J* = 14.4 Hz, 1H), 1.95 (dd, *J* = 13.6, 4.8 Hz, 1H); ^13^C{^1^H} NMR (100.3 MHz, CDCl_3_) δ 178.1, 147.1, 144.9, 140.6, 137.4, 129.5, 129.1, 128.0, 126.2, 120.9, 114.4, 110.6, 109.5, 67.3, 59.2, 55.9, 55.3, 47.4, 33.6, 33.1, 25.4; IR (ATR) 3308, 1714 cm^–1^; HRMS (ESI-TOF) calcd for C_21_H_22_N_2_O_3_Cl [M + H]^+^): 385.1314, found 385.1311.

#### (1′S*,3R*,4′R*,5′S*)-4′-Chloro-8′,9′-dimethoxy-5-nitro-3′,4′,5′,6′-tetrahydrospiro[indoline-3,2′-[1,5]methanobenzo[*b*]azocin]-2-one (**6b**)

Procedure B was generally followed to synthesize **6b** from **1** (22 mg, 0.1 mmol, 1.0 equiv) and **5b** (48 mg, 0.25 mmol, 2.5 equiv). The residue was purified by flash column chromatography on silica gel (hexane/EtOAc, 2:1–1:1) to afford **6b** (30 mg, 0.069 mmol, 69%) as a pale yellow solid: mp 243.9–244.9 °C; ^1^H NMR (400 MHz, CDCl_3_) δ 8.19 (dd, *J* = 8.4, 2.4 Hz, 1H), 8.06 (br s, 1H), 6.98 (d, *J* = 8.8 Hz, 1H), 6.84 (d, *J* = 1.6 Hz, 1H), 6.75 (s, 1H), 5.58 (s, 1H), 5.24 (dt, *J* = 12.4, 4.4 Hz, 1H), 4.42 (dd, *J* = 13.2, 0.8 Hz, 1H), 3.89 (s, 3H), 3.34–3.30 (m, 4H), 3.06–2.95 (m, 2H), 2.56 (br s, 1H), 2.14 (t, *J* = 13.6 Hz, 1H), 1.99 (dd, *J* = 14.2, 4.8 Hz, 1H); ^13^C{^1^H} NMR (100.3 MHz, CDCl_3_) δ 177.2, 148.0, 146.1, 145.7, 142.2, 136.7, 130.0, 126.6, 126.3, 124.0, 114.1, 111.8, 109.3, 66.9, 58.3, 56.3, 55.7, 47.6, 33.5, 32.8, 25.4; IR (ATR) 3096, 1715 cm^–1^; HRMS (ESI-TOF) calcd for C_21_H_21_N_3_O_5_Cl [M + H]^+^ 430.1164, found 430.1168.

#### (1′S*,3R*,4′R*,5′S*)-4′-Chloro-8′,9′-dimethoxy-5-(trifluoromethoxy)-3′,4′,5′,6′-tetrahydrospiro[indoline-3,2′-[1,5]methanobenzo[*b*]azocin]-2-one (**6c**)

Procedure B was generally followed to synthesize **6c** from **1** (22 mg, 0.1 mmol, 1.0 equiv) and **5c** (58 mg, 0.25 mmol, 2.5 equiv). The residue was purified by flash column chromatography on silica gel (hexane/EtOAc, 3:2) to afford **6c** (30 mg, 0.063 mmol, 63%) as pale yellow solid. The single crystal for X-ray crystallographic analysis was obtained by recrystallization of **6c** from hexane/acetone: mp 212.3–213.3 °C; ^1^H NMR (400 MHz, CDCl_3_) δ 7.54 (br s, 1H), 7.09 (d, *J* = 10.0 Hz, 1H), 6.85 (d, *J* = 8.4 Hz, 1H), 6.70 (s, 1H), 5.84 (d, *J* = 2.0 Hz, 1H), 5.65 (s, 1H), 5.26 (dt, *J* = 10.8, 4.8 Hz, 1H), 4.47 (d, *J* = 14.2 Hz, 1H), 3.88 (s, 3H), 3.44 (s, 3H), 3.29 (d, *J* = 18.8 Hz, 1H), 3.02–2.92 (m, 2H), 2.54 (br s, 1H), 2.08–2.02 (m, 1H), 1.97 (dd, *J* = 13.9, 5.2 Hz, 1H); ^13^C{^1^H} NMR (100.3 MHz, CDCl_3_) δ 177.8, 147.5, 145.7, 143.4, 139.1, 137.1, 130.7, 126.1, 122.7, 121.7, 120.4 (q, *J*_C–F_ = 257.2 Hz), 113.6, 111.2, 110.0, 67.4, 58.8, 56.1, 55.2, 47.6, 33.6, 33.1, 25.3; IR (ATR) 3185, 1714 cm^–1^; HRMS (ESI-TOF) calcd for C_22_H_21_N_2_O_4_F_3_Cl [M + H]^+^ 469.1137, found 469.1140.

#### (1′S*,3R*,4′R*,5′S*)-4′-Chloro-5-fluoro-8′,9′-dimethoxy-3′,4′,5′,6′-tetrahydrospiro[indoline-3,2′-[1,5]methanobenzo[*b*]azocin]-2-one (**6d**)

Procedure B was generally followed to synthesize **6d** from **1a** (22 mg, 0.1 mmol, 1.0 equiv) and **5d** (41 mg, 0.25 mmol, 2.5 equiv). The residue was purified by flash column chromatography on silica gel (hexane/EtOAc, 1:1) to afford **6d** (26 mg, 0.067 mmol, 67%) as a pale yellow solid: mp 205.4–205.9 °C; ^1^H NMR (400 MHz, CDCl_3_) δ 7.72 (br s, 1H), 6.92 (td, *J* = 8.4, 2.8 Hz, 1H), 6.79 (dd, *J* = 8.4, 4.2 Hz, 1H), 6.70 (s, 1H), 5.71 (dd, *J* = 8.4, 2.4 Hz, 1H), 5.69 (s, 1H), 5.28 (dt, *J* = 10.8, 5.6 Hz, 1H), 4.50 (d, *J* = 14.0 Hz, 1H), 3.88 (s, 3H), 3.48 (s, 3H), 3.28 (d, *J* = 18.8 Hz, 1H), 3.02–2.92 (m, 2H), 2.53 (br s, 1H), 2.06–1.93 (m, 2H); ^13^C{^1^H} NMR (100.3 MHz, CDCl_3_) δ 178.1, 157.6 (d, *J*_C–F_ = 240.9 Hz), 147.4, 145.3, 137.0, 136.5, 130.7 (d, *J*_C–F_ = 8.7 Hz), 116.0 (d, *J*_C–F_ = 20.2 Hz), 115.8 (d, *J*_C–F_ = 17.3 Hz), 114.5, 110.9, 110.1 (d, *J*_C–F_ = 7.7 Hz), 67.6, 58.8, 56.0, 55.5, 47.5, 33.5, 33.1, 25.5; IR (ATR) 3170, 1708 cm^–1^; HRMS (ESI-TOF) calcd for C_21_H_21_N_2_O_3_FCl [M + H]^+^ 403.1219, found 403.1221.

#### (1′S*,3R*,4′R*,5′S*)-5-Bromo-4′-chloro-8′,9′-dimethoxy-3′,4′,5′,6′-tetrahydrospiro[indoline-3,2′-[1,5]methanobenzo[*b*]azocin]-2-one (**6e**)

Procedure B was generally followed to synthesize **6e** from **1** (22 mg, 0.1 mmol, 1.0 equiv) and **5e** (57 mg, 0.25 mmol, 2.5 equiv). The residue was purified by flash column chromatography on silica gel (hexane/EtOAc, 2:1–1:1) to afford **6e** (35 mg, 0.074 mmol, 74%) as a pale yellow solid: mp 249.5–250.4 °C; ^1^H NMR (400 MHz, CDCl_3_) δ 7.78 (br s, 1H), 7.34 (dd, *J* = 8.2, 2.0 Hz, 1H), 6.75 (d, *J* = 8.4 Hz, 1H), 6.70 (s, 1H), 6.05 (d, *J* = 1.6 Hz, 1H), 5.66 (s, 1H), 5.27 (dt, *J* = 12.0, 4.8 Hz, 1H), 4.47 (d, *J* = 12.8 Hz, 1H), 3.89 (s, 3H), 3.52 (s, 3H), 3.28 (d, *J* = 18.4 Hz, 1H), 3.03–2.92 (m, 2H), 2.53 (br s, 1H), 2.10–1.93 (m, 2H); ^13^C{^1^H} NMR (100.3 MHz, CDCl_3_) δ 177.1, 147.5, 145.4, 139.4, 137.0, 132.2, 131.4, 131.1, 126.2, 114.3, 113.7, 111.2, 110.8, 67.4, 58.7, 56.1, 55.5, 47.4, 33.5, 33.0, 25.5; IR (ATR) 3170, 1709 cm^–1^; HRMS (ESI-TOF) calcd for C_21_H_21_N_2_O_3_ClBr [M + H]^+^ 463.0419, found 463.0419.

#### (1′S*,3R*,4′R*,5′S*)-4′-Chloro-5,8′,9′-trimethoxy-3′,4′,5′,6′-tetrahydrospiro[indoline-3,2′-[1,5]methanobenzo[*b*]azocin]-2-one (**6f**)

Procedure B was generally followed to synthesize **6f** from **1** (22 mg, 0.1 mmol, 1.0 equiv) and **5f** (44 mg, 0.25 mmol, 2.5 equiv). The residue was purified by flash column chromatography on silica gel (hexane/EtOAc, 3:2–1:1) to afford **6f** (14 mg, 0.033 mmol, 33%) as a pale yellow solid: mp 210.1–211.0 °C; ^1^H NMR (400 MHz, CDCl_3_) δ 7.31 (s, 1H), 6.74 (s, 2H), 6.69 (s, 1H), 5.67 (s, 1H), 5.52 (s, 1H), 5.31 (dt, *J* = 11.6, 4.8 Hz, 1H), 4.52 (d, *J* = 13.6 Hz, 1H), 3.87 (s, 3H), 3.49 (s, 3H), 3.45 (s, 3H), 3.29 (d, *J* = 18.4 Hz, 1H), 3.03–2.92 (m, 2H), 2.53 (br s, 1H), 2.07–1.94 (m, 2H); ^13^C{^1^H} NMR (100.3 MHz, CDCl_3_) δ 178.2, 154.2, 147.1, 145.0, 137.2, 133.9, 130.1, 126.3, 115.0, 114.6, 114.3, 110.8, 110.0, 67.7, 59.2, 56.0, 55.5, 55.4, 47.4, 33.6, 33.2, 25.5; IR (ATR) 3291, 1704 cm^–1^; HRMS (ESI-TOF) calcd for C_22_H_24_N_2_O_4_Cl [M + H]^+^ 415.1419, found 415.1414.

#### (1′S*,3R*,4′R*,5′S*)-4′-Chloro-8′,9′-dimethoxy-1-methyl-3′,4′,5′,6′-tetrahydrospiro[indoline-3,2′-[1,5]methanobenzo[*b*]azocin]-2-one (**6g**)

Procedure B was generally followed to synthesize **5g** from **1** (22 mg, 0.1 mmol, 1.0 equiv) and **5g** (40 mg, 0.25 mmol, 2.5 equiv). The residue was purified by flash column chromatography on silica gel (hexane/EtOAc, 2:1) to afford **6g** (33 mg, 0.082 mmol, 82%) as a pale yellow amorphous solid: ^1^H NMR (400 MHz, CDCl_3_) δ 7.25 (m, 1H), 6.78 (m, 2H), 6.68 (s, 1H), 5.93 (d, *J* = 7.2 Hz, 1H), 5.60 (s, 1H), 5.35 (dt, *J* = 12.4, 5.2 Hz, 1H), 4.56 (d, *J* = 12.8 Hz, 1H), 3.88 (s, 3H), 3.40 (s, 3H), 3.29 (d, *J* = 18.4 Hz, 1H), 3.21 (s, 3H), 2.97 (m, 2H), 2.55 (br s, 1H), 2.09 (t, *J* = 14.4 Hz, 1H), 1.90 (dd, *J* = 14.0, 4.4 Hz, 1H); ^13^C{^1^H} NMR (100.3 MHz, CDCl_3_) δ 176.0, 147.0, 144.9, 143.5, 137.5, 129.5, 128.5, 127.6, 126.2, 120.9, 114.3, 110.5, 107.9, 67.0, 59.3, 55.9, 55.3, 47.4, 33.6, 33.1, 26.1, 25.5; IR (ATR) 1696 cm^–1^; HRMS (ESI-TOF) calcd for C_22_H_24_N_2_O_3_Cl [M + H]^+^ 399.1470, found 399.1471.

#### (1′S*,3R*,4′R*,5′S*)-4′,6-Dichloro-8′,9′-dimethoxy-3′,4′,5′,6′-tetrahydrospiro[indoline-3,2′-[1,5]methanobenzo[*b*]azocin]-2-one (**6h**)

Procedure B was generally followed to synthesize **6h** from **1** (22 mg, 0.1 mmol, 1.0 equiv), **5h** (45 mg, 0.25 mmol, 2.5 equiv), and 4 M HCl in dioxane (0.125 mL, 0.50 mmol, 5.0 equiv). The residue was purified by flash column chromatography on acidic silica gel (hexane/EtOAc, 7:1) to afford **6h** (21 mg, 0.050 mmol, 50%) as pale yellow amorphous solid: ^1^H NMR (400 MHz, CDCl_3_) δ 7.88 (s, 1H), 6.88 (d, *J* = 2.0 Hz, 1H), 6.74 (dd, *J* = 8.2, 2.0 Hz, 1H), 6.68 (s, 1H), 5.83 (d, *J* = 8.0 Hz, 1H), 5.65 (s, 1H), 5.26 (dt, *J* = 12.4, 4.8 Hz, 1H), 4.46 (d, *J* = 13.6 Hz, 1H), 3.88 (s, 3H), 3.46 (s, 3H), 3.28 (d, *J* = 18.4 Hz, 1H), 3.01 (dd, *J* = 14.1, 2.7 Hz, 1H), 2.95 (dd, J = 18.5, 8.4 Hz, 1H), 2.54 (br s, 1H), 2.07–2.01 (m, 1H), 1.93 (dd, *J* = 14.1, 5.0 Hz, 1H); ^13^C{^1^H} NMR (100.3 MHz, CDCl_3_) δ 177.6, 147.2, 145.1, 141.54, 137.2, 135.3, 129.1, 127.5, 126.3, 121.0, 114.2, 110.7, 110.0, 66.9, 58.9, 55.9, 55.5, 47.4, 33.5, 33.0, 25.4; IR (ATR) 3167, 1705 cm^–1^; HRMS (ESI-TOF) calcd for C_21_H_21_N_2_O_3_Cl_2_ [M + H]^+^ 419.0924, found 419.0924.

#### (1′S*,3R*,4′R*,5′S*)-4′,7-Dichloro-8′,9′-dimethoxy-3′,4′,5′,6′-tetrahydrospiro[indoline-3,2′-[1,5]methanobenzo[*b*]azocin]-2-one (**6i**)

Procedure B was generally followed to synthesize **6i** from **1** (22 mg, 0.1 mmol, 1.0 equiv) and **5i** (45 mg, 0.25 mmol, 2.5 equiv). The residue was purified by flash column chromatography on silica gel (hexane/EtOAc, 3:1) to afford **6i** (29 mg, 0.069 mmol, 69%) as a pale yellow amorphous solid: ^1^H NMR (400 MHz, CDCl_3_) δ 8.01 (br s, 1H), 7.19 (d, *J* = 8.4 Hz, 1H), 6.69 (m, 2H), 5.81 (d, *J* = 7.2 Hz, 1H), 5.63 (s, 1H), 5.27 (dt, *J* = 7.3, 4.6 Hz, 1H), 4.47 (d, *J* = 13.6 Hz, 1H), 3.88 (s, 3H), 3.42 (s, 3H), 3.28 (d, *J* = 18.8 Hz, 1H), 3.01 (dd, *J* = 13.9, 2.5 Hz, 1H), 2.95 (dd, *J* = 18.7, 8.7 Hz, 1H), 2.53 (br s, 1H), 2.05 (t, 13.2 Hz, 1H), 1.96 (dd, *J* = 13.9, 5.2 Hz, 1H); ^13^C{^1^H} NMR (100.3 MHz, CDCl_3_) δ 176.9, 147.2, 145.0, 138.5, 137.2, 130.4, 129.3, 126.3, 126.3, 121.7, 114.8, 114.3, 110.6, 68.3, 58.9, 55.9, 55.4, 47.4, 33.5, 29.6, 25.4. IR (ATR) 3191, 1713 cm^–1^; HRMS (ESI-TOF) calcd for C_21_H_21_N_2_O_3_Cl_2_ [M + H]^+^ 419.0924, found 419.0922.

#### (1′S*,3R*,4′R*,5′S*)-4′-Chloro-8′,9′-dimethoxy-6-(trifluoromethyl)-3′,4′,5′,6′-tetrahydrospiro[indoline-3,2′-[1,5]methanobenzo[*b*]azocin]-2-one (**6k**)

Procedure B was generally followed to synthesize **6k** from **1** (44 mg, 0.20 mmol, 1.0 equiv) and **5k** (108 mg, 0.50 mmol, 2.5 equiv). The residue was purified by flash column chromatography on alumina (CHCl_3_) to afford **6k** (59 mg, 0.13 mmol, 66%) as a colorless solid: mp 217.9–218.6 °C; ^1^H NMR (400 MHz, CDCl_3_) δ 8.54 (s, 1H), 7.13 (s, 1H), 7.04 (d, *J* = 8.7 Hz, 1H), 6.70 (s, 1H), 6.01 (d, *J* = 7.6 Hz, 1H), 5.57 (s, 1H), 5.27 (dt, *J* = 12.3, 4.3 Hz, 1H), 4.47 (d, *J* = 14.0 Hz, 1H), 3.88 (s, 3H), 3.39 (s, 3H), 3.30 (d, *J* = 18.8 Hz, 1H), 3.03 (dd, *J* = 13.7, 2.7 Hz, 1H), 2.97 (dd, *J* = 18.7, 8.7 Hz, 1H), 2.56 (br s, 1H), 2.15–2.06 (m, 1H), 1.97 (dd, *J* = 14.1, 5.0 Hz, 1H) ; ^13^C NMR (100 MHz, CDCl_3_) δ 177.7, 147.4, 145.1, 141.2, 137.1, 132.8, 131.9 (q, *J*_C–F_ = 32.8 Hz), 128.4, 126.3, 123.6 (q, *J*_C–F_ = 273 Hz), 118.0 (br), 114.1, 110.8, 106.3 (br), 67.1, 58.7, 55.9, 55.3, 47.5, 33.5, 32.7, 25.3; IR (ATR) 3276, 1715 cm^–1^; HRMS (ESI-TOF) calcd for C_22_H_21_ClF_3_N_2_O_3_ [M + H]^+^ 453.1187, found 453.1187.

#### (1′S*,3R*,4′R*,5′S*)-4′-Chloro-6,8′,9′-trimethoxy-3′,4′,5′,6′-tetrahydrospiro[indoline-3,2′-[1,5]methanobenzo[*b*]azocin]-2-one (**6l**)

Procedure B was generally followed to synthesize **6l** from **1** (263 mg, 1.2 mmol, 1.0 equiv) and **5l** (532 mg, 3.0 mmol, 2.5 equiv). The residue was purified by flash column chromatography on alumina (CHCl_3_) and then silica gel (hexane/MTBE, 1:2) to afford **6l** (17 mg, 0.041 mmol, 3.4%) as a colorless solid: mp 217.5 °C (decomp); ^1^H NMR (400 MHz, CDCl_3_) δ 7.28 (s, 1H), 6.68 (s, 1H), 6.40 (s, 1H), 6.26 (d, *J* = 8.4 Hz, 1H), 5.81 (d, *J* = 8.8 Hz, 1H), 5.68 (s, 1H), 5.28 (dt, *J* = 11.9, 4.6 Hz, 1H), 4.47 (d, *J* = 14.0 Hz, 1H), 3.88 (s, 3H), 3.74 (s, 3H), 3.46 (s, 3H), 3.27 (d, *J* = 18.4 Hz, 1H), 3.01 (dd, *J* = 13.5, 1.6 Hz, 1H), 2.94 (dd, *J* = 18.3, 8.2 Hz, 1H), 2.52 (br s, 1H), 2.03 (t, *J* = 13.2 Hz, 1H), 1.93 (dd, *J* = 13.9, 4.8 Hz, 1H); ^13^C NMR (100 MHz, CDCl_3_) δ 177.8, 160.9, 147.0, 144.9, 141.5, 137.5, 129.0, 126.2, 121.2, 114.4, 110.6, 105.2, 96.6, 66.9, 59.4, 55.9, 55.5, 55.5, 47.4, 33.6, 33.4, 25.5; IR (ATR) 3209, 1698 cm^–1^; HRMS (ESI-TOF) calcd for C_22_H_25_ClN_2_O_4_ [M + H]^+^ 415.1419, found 415.1419.

### X-ray Diffraction Studies

All diffraction data were collected at −173 °C on a Bruker Apex II Ultra X-ray diffractometer equipped with a Mo Kα radiation source (λ = 0.71073 Å). Intensity data were processed using the Apex3 software. The structure solution and refinements were carried out using the Yadokari-XG^12^ graphical interface. The positions of the non-hydrogen atoms were determined using the SHELXT^13^ program and refined on *F*^2^ by full-matrix least-squares techniques using the SHELXL^14^ program. All non-hydrogen atoms were refined with anisotropic thermal parameters, while all hydrogen atoms were placed using AFIX instructions. Details of the diffraction data are summarized in Tables S1–S5.

## Supporting Information Available

Molecular structures; details of the diffraction data; HPLC analyses of *rac***-4**, **2***S***-4**, **2***R***-4**; copies of NMR spectra and X-ray crystallographic data for **3a**, **3e**, **3f**, **2***S***-3i**, and **6c** (PDF)

## Accession Codes

CCDC 2077096–2077100 contain the supplementary crystallographic data for this paper. These data can be obtained free of charge via www.ccdc.cam.ac.uk/data_request/cif, or by emailing data_request@ccdc.cam.ac.uk, or by contacting The Cambridge Crystallographic Data Centre, 12 Union Road, Cambridge CB2 1EZ, UK; fax: + 44 1223 336033.
