# Supplementary material for: The Aza-Prins Reaction of 1,2-Dicarbonyl Compounds with 3-Vinyltetrahydroquinolines: Application to the Synthesis of Polycyclic Spirooxindole Derivatives
Source: J Org Chem. 2021 Nov 18;86(23):16425–33. doi: 10.1021/acs.joc.1c01785 (PMC8650011; doi:10.1021/acs.joc.1c01785)

Supporting Information

The Aza-Prins Reaction of 1,2-Dicarbonyl Compounds with 3-Vinyltetrahydroquinolines: Application to the Synthesis of Polycyclic Spirooxindole Derivatives

Shinichi Saito,* Tomohiro Katamura, Rei Tsukazaki, Akito Fujisawa, Yusuke Yoshigoe, and Yuichiro Mutoh

Department of Chemistry, Faculty of Science, Tokyo University of Science, Kagurazaka, Shinjuku, Tokyo 162-8601, Japan

1. Molecular Structures, Crystal Data and Structure Refinement of 3a, 3e, 3f, 2*S*-3g, and 6c (Figures S1-S5, Tables S1-S5). **S2**

2. HPLC Analyses of rac-4, 2R-4, and 2S-4 (Figure S6). **S12**

2. NMR Spectra **S13-S37**

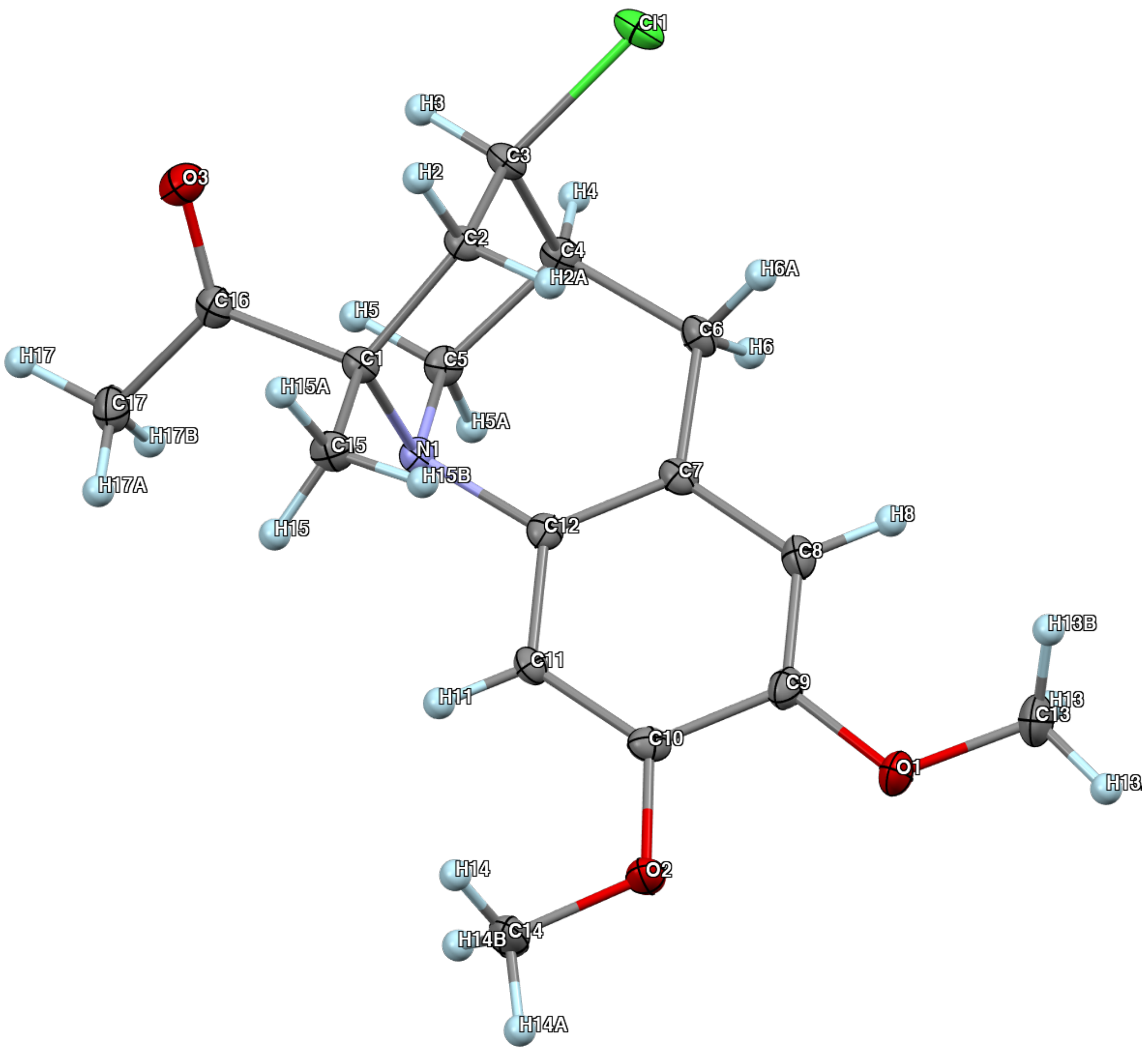

Figure S1. Molecular structure of **3a** with thermal ellipsoids at 50% probability (CCDC 2077096).

Table S1. Crystal Data and Structure Refinement for 3a.

Identification code	RT258	
CCDC	2077096	
Empirical formula	$C_{17}H_{22}ClNO_3$	
Formula weight	323.80	
Temperature	100(2) K	
Wavelength	0.71073 Å	
Crystal system	Monoclinic	
Space group	$P2_1/c$	
Unit cell dimensions	a = 17.523(4) Å	α = 90°
	b = 8.0497(16) Å	β = 103.821(2)°
	c = 11.780(2) Å	γ = 90°
Volume	1613.4(6) Å^3	
Z	4	
Density (calculated)	1.333 Mg/m^3	
Absorption coefficient	0.249 mm^{-1}	
F(000)	688	
Crystal size	0.370 × 0.110 × 0.050 mm^3	
Theta range for data collection	2.394 to 27.493°	
Index ranges	$-18 \leq h \leq 22$, $-10 \leq k \leq 10$, $-15 \leq l \leq 8$	
Reflections collected	8392	
Independent reflections	3560 (R_{int} = 0.0287)	
Completeness to theta = 25.242°	99.9%	
Refinement method	Full-matrix least-squares on F^2	
Data / restraints / parameters	3560 / 0 / 203	
Goodness-of-fit on F^2	1.032	
Final R indices [$I > 2\sigma(I)$]	R_1 = 0.0350, wR_2 = 0.0884	
R indices (all data)	R_1 = 0.0424, wR_2 = 0.0931	
Largest diff. peak and hole	0.363 and −0.251 e Å^{-3}	

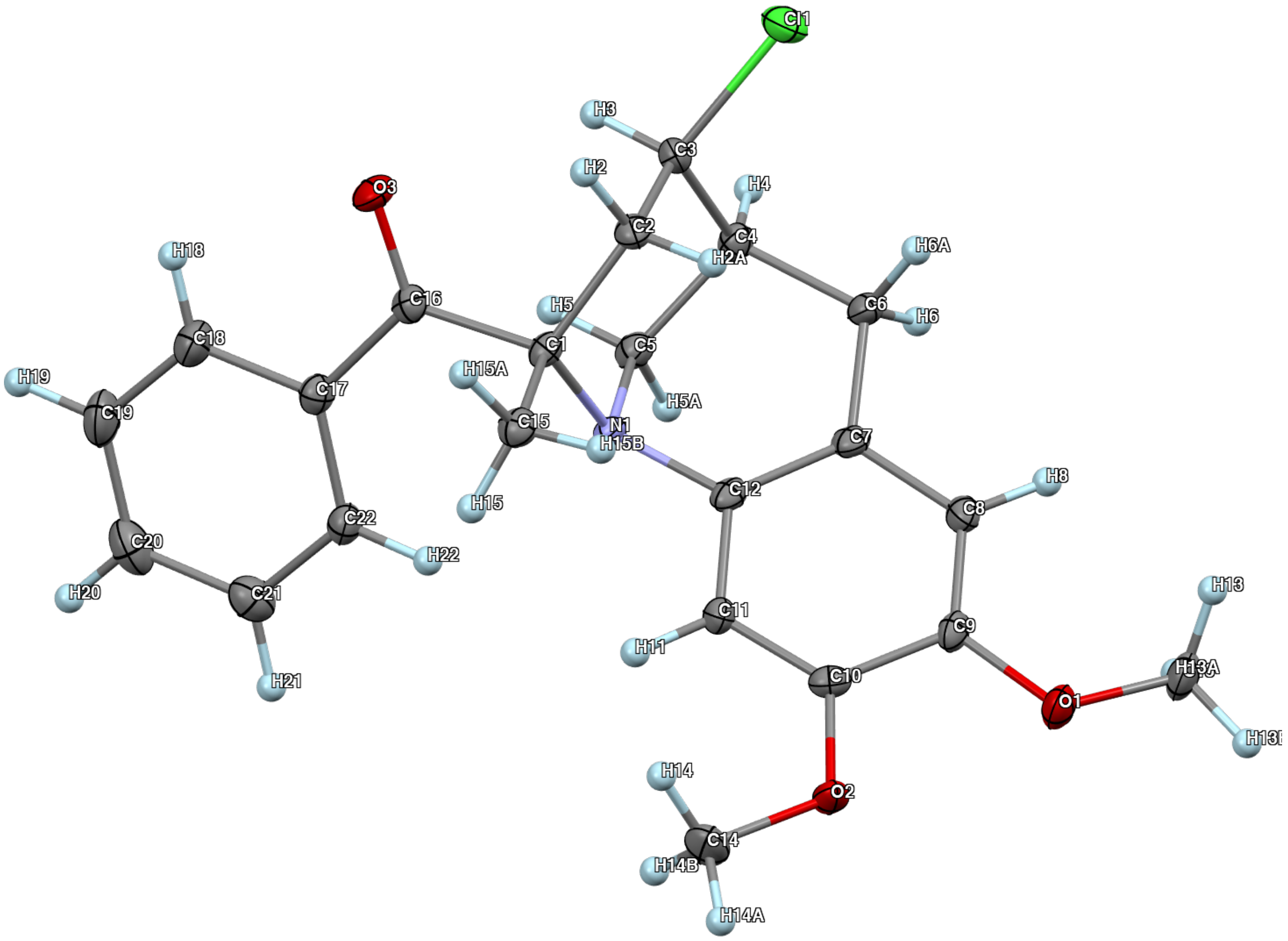

Figure S2. Molecular structure of **3e** with thermal ellipsoids at 50% probability (CCDC 2077097).

Table S2. Crystal Data and Structure Refinement for 3e.

Identification code	RT2635	
CCDC	2077097	
Empirical formula	$C_{22}H_{24}ClNO_3$	
Formula weight	385.87	
Temperature	100(2) K	
Wavelength	0.71073 Å	
Crystal system	Triclinic	
Space group	$P\bar{1}$	
Unit cell dimensions	a = 11.954(4) Å	α = 73.622(4)°
	b = 12.661(4) Å	β = 68.508(4)°
	c = 15.437(5) Å	γ = 62.551(4)°
Volume	1910.9(11) $Å^3$	
Z	4	
Density (calculated)	1.341 Mg/m^3	
Absorption coefficient	0.223 mm^{-1}	
F(000)	816	
Crystal size	0.260 × 0.070 × 0.030 mm^3	
Theta range for data collection	1.830 to 27.442°	
Index ranges	$-15 \leq h \leq 15$, $-16 \leq k \leq 16$, $-19 \leq l \leq 19$	
Reflections collected	20783	
Independent reflections	8378 (R_{int} = 0.0768)	
Completeness to theta = 25.242°	99.4%	
Refinement method	Full-matrix least-squares on F^2	
Data / restraints / parameters	8378 / 0 / 493	
Goodness-of-fit on F^2	1.003	
Final R indices [$I > 2\sigma(I)$]	R_1 = 0.0542, wR_2 = 0.1024	
R indices (all data)	R_1 = 0.1092, wR_2 = 0.1248	
Largest diff. peak and hole	0.316 and −0.328 e $Å^{-3}$	

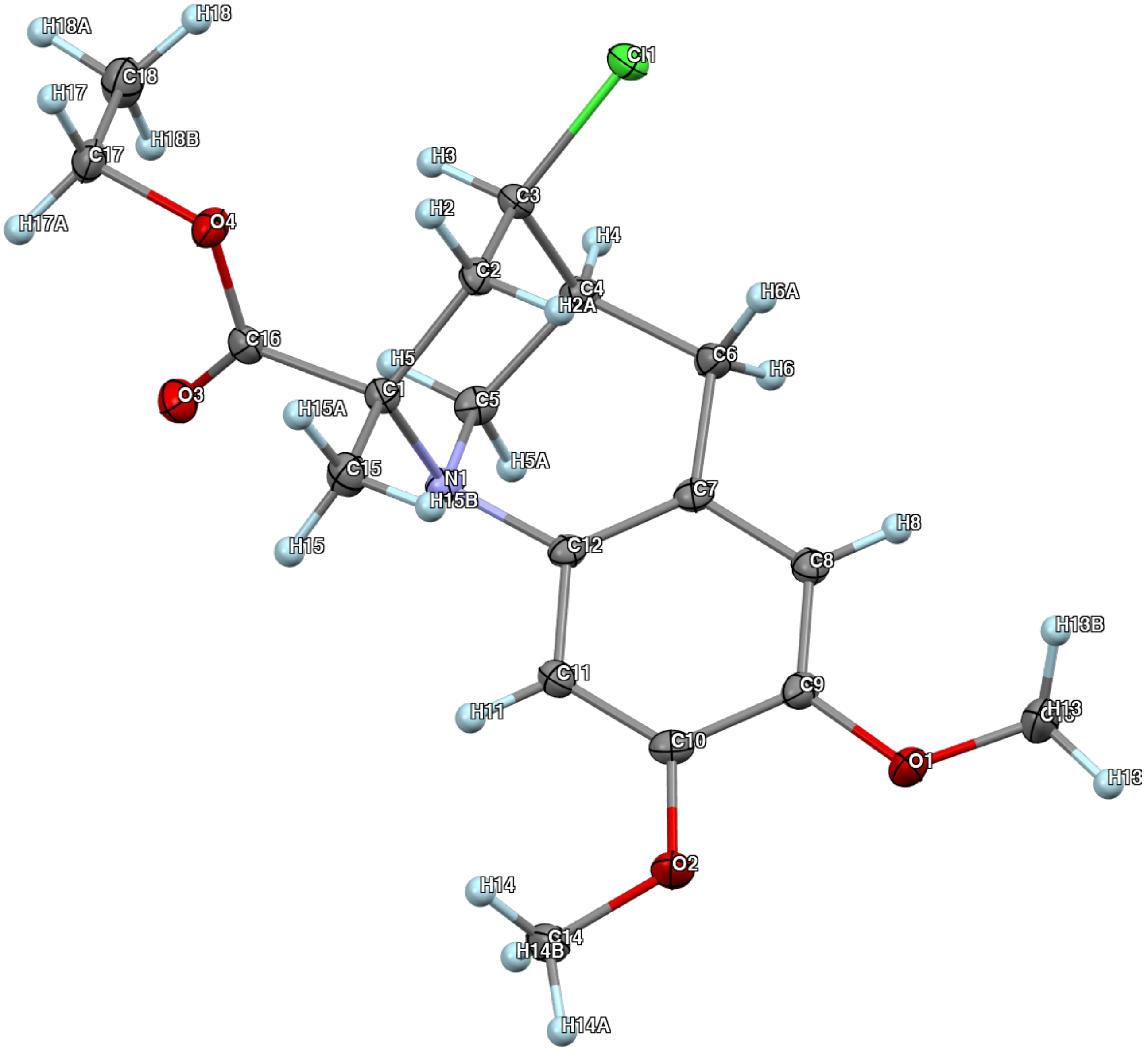

Figure S3. Molecular structure of **3f** with thermal ellipsoids at 50% probability (CCDC 2077098).

Table S3. Crystal Data and Structure Refinement for 3f.

Identification code	TK 3-31-4	
CCDC	2077098	
Empirical formula	$C_{18}H_{24}ClNO_4$	
Formula weight	353.83	
Temperature	100(2) K	
Wavelength	0.71073 Å	
Crystal system	Orthorhombic	
Space group	*Pbcn*	
Unit cell dimensions	a = 18.8473(12) Å	α = 90°
	b = 8.8466(6) Å	β = 90°
	c = 20.3714(13) Å	γ = 90°
Volume	3396.6(4) Å^3	
Z	8	
Density (calculated)	1.384 Mg/m^3	
Absorption coefficient	0.247 mm^{-1}	
F(000)	1504	
Crystal size	0.250 × 0.100 × 0.090 mm^3	
Theta range for data collection	1.999 to 27.500°.	
Index ranges	$-24 \leq h \leq 23$, $-11 \leq k \leq 11$, $-25 \leq l \leq 26$	
Reflections collected	35361	
Independent reflections	3889 (R_{int} = 0.0337)	
Completeness to theta = 25.242°	99.9%	
Refinement method	Full-matrix least-squares on F^2	
Data / restraints / parameters	3889 / 0 / 221	
Goodness-of-fit on F^2	1.040	
Final R indices [$I > 2\sigma(I)$]	R_1 = 0.0334, wR_2 = 0.0861	
R indices (all data)	R_1 = 0.0394, wR_2 = 0.0909	
Largest diff. peak and hole	0.417 and −0.381 e Å^{-3}	

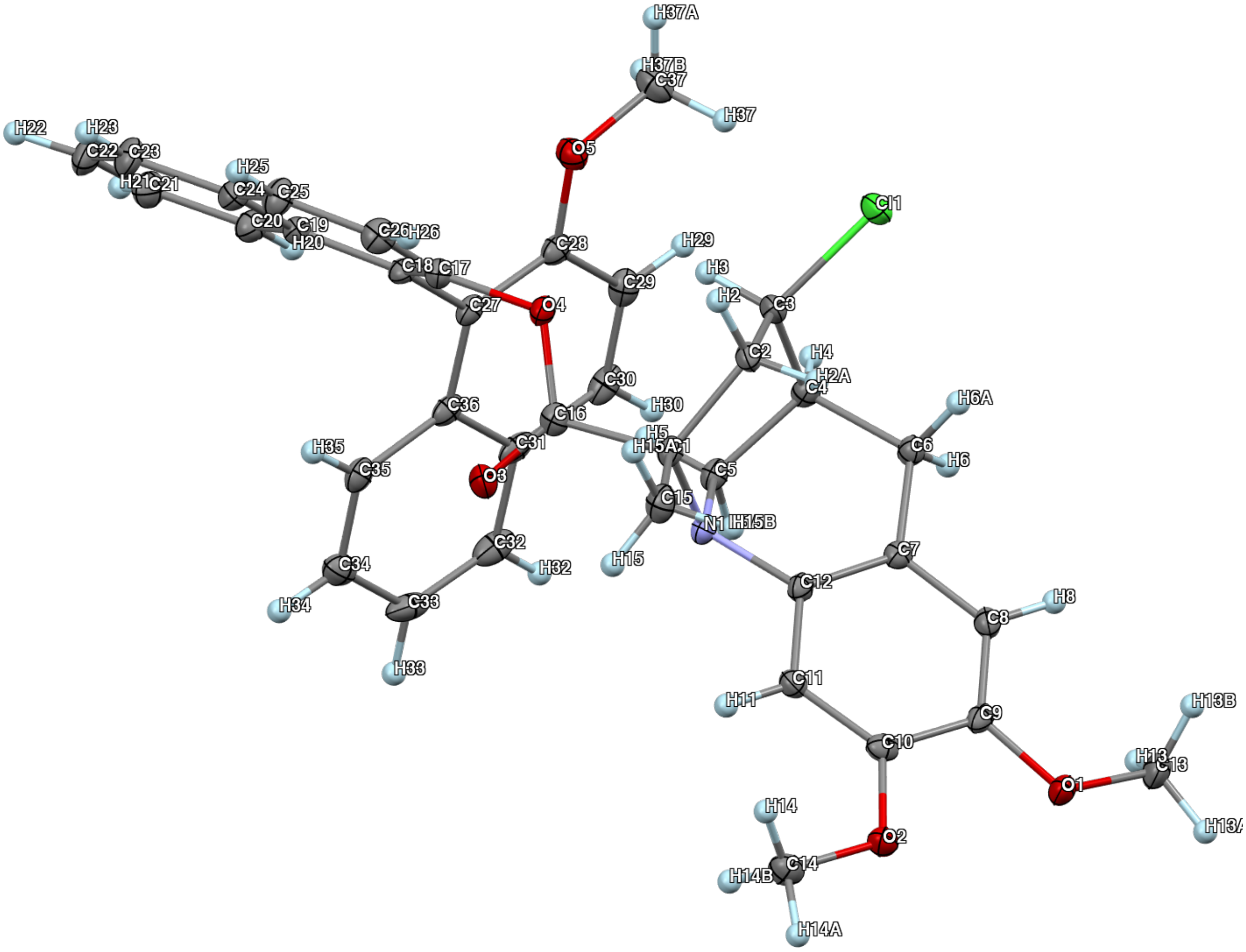

Figure S4. Molecular structure of **2*S*-3i** with thermal ellipsoids at 50% probability (CCDC 2077099).

Table S4. Crystal Data and Structure Refinement for 2*S*-3i.

Identification code	RT41921	
CCDC	2077099	
Empirical formula	$C_{75}H_{70}Cl_4N_2O_{10}$	
Formula weight	1301.13	
Temperature	100(2) K	
Wavelength	0.71073 Å	
Crystal system	Monoclinic	
Space group	*C*2	
Unit cell dimensions	a = 17.3441(17) Å	α = 90°
	b = 8.4657(8) Å	β = 109.0670(10)°
	c = 22.671(2) Å	γ = 90°
Volume	3146.1(5) $Å^3$	
Z	2	
Density (calculated)	1.373 Mg/m^3	
Absorption coefficient	0.253 mm^{-1}	
F(000)	1364	
Crystal size	0.210 × 0.140 × 0.140 mm^3	
Theta range for data collection	1.901 to 27.491°.	
Index ranges	$-22 \leq h \leq 22$, $-10 \leq k \leq 10$, $-29 \leq l \leq 28$	
Reflections collected	17655	
Independent reflections	7078 (R_{int} = 0.0259)	
Completeness to theta = 25.242°	100.0%	
Absorption correction	Semi-empirical from equivalents	
Max. and min. transmission	0.965 and 0.913	
Refinement method	Full-matrix least-squares on F^2	
Data / restraints / parameters	7078 / 1 / 415	
Goodness-of-fit on F^2	1.036	
Final *R* indices [$I > 2\sigma(I)$]	R_1 = 0.0361, wR_2 = 0.0829	
R indices (all data)	R_1 = 0.0391, wR_2 = 0.0848	
Absolute structure parameter	0.03(2)	
Largest diff. peak and hole	0.667 and -0.445 e $Å^{-3}$	

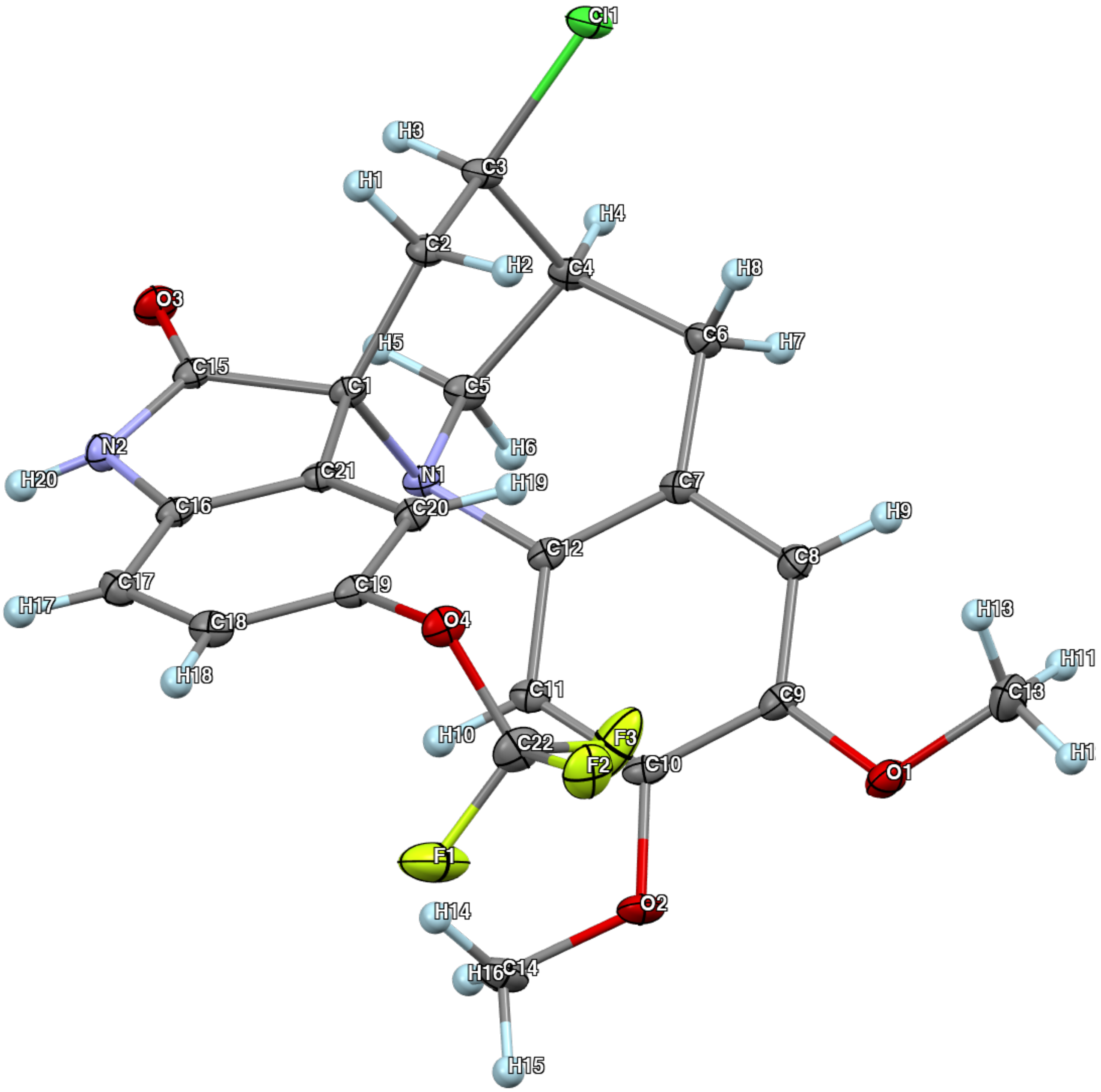

Figure S5. Molecular structure of **6c** with thermal ellipsoids at 50% probability (CCDC 2077100).

Table S5. Crystal Data and Structure Refinement for 6c.

Identification code	TK5662
CCDC	2077100
Empirical formula	C22 H20 Cl F3 N2 O4
Formula weight	468.85
Temperature	100(2) K
Wavelength	0.71073 Å
Crystal system	Triclinic
Space group	$P\bar{1}$
Unit cell dimensions	a = 10.0772(11) Å α = 80.4170(10)°
	b = 10.4364(11) Å β = 68.0910(10)°
	c = 10.6612(11) Å γ = 70.6730(10)°
Volume	980.45(18) $Å^3$
Z	2
Density (calculated)	1.588 Mg/m^3
Absorption coefficient	0.258 mm^{-1}
F(000)	484
Crystal size	0.460 × 0.420 × 0.300 mm^3
Theta range for data collection	2.061 to 27.497°.
Index ranges	$-13 \leq h \leq 13$, $-13 \leq k \leq 13$, $-13 \leq l \leq 13$
Reflections collected	10743
Independent reflections	4359 (R_{int} = 0.0317)
Completeness to theta = 25.242°	99.0%
Refinement method	Full-matrix least-squares on F^2
Data / restraints / parameters	4359 / 0 / 294
Goodness-of-fit on F^2	1.192
Final R indices [$I > 2\sigma(I)$]	R_1 = 0.0458, wR_2 = 0.1191
R indices (all data)	R_1 = 0.0477, wR_2 = 0.1212
Largest diff. peak and hole	0.462 and −0.672 e $Å^{-3}$

Figure S6. HPLC analyses of ***rac*-4, 2*S*-4,** and **2*R*-4**

Sample; ***rac*-4**

(conc. = 1 mg/mL in eluent)

Column; CHIRAL ART Amylose-SA, 5 μm

(250 x 4.6 mm I.D.)

Inject; 10 μL

Eluent; *n*-Hex : isopropyl alcohol : diethylamine

= 80 : 20 : 0.1

Flow Rate; 1.0 mL/min

Detection; 254 nm

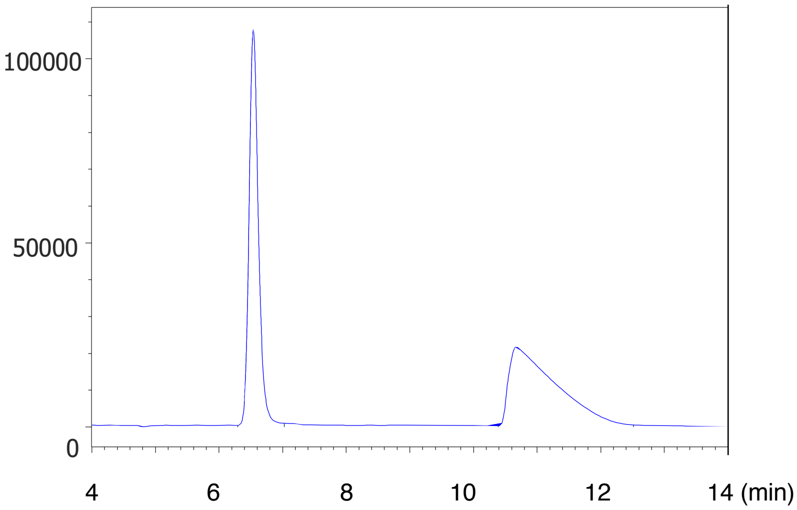

HPLC analysis of ***rac*-4**

Sample; **2*S*-4** and **2*R*-4**

(conc. = 0.5 mg/mL in eluent)

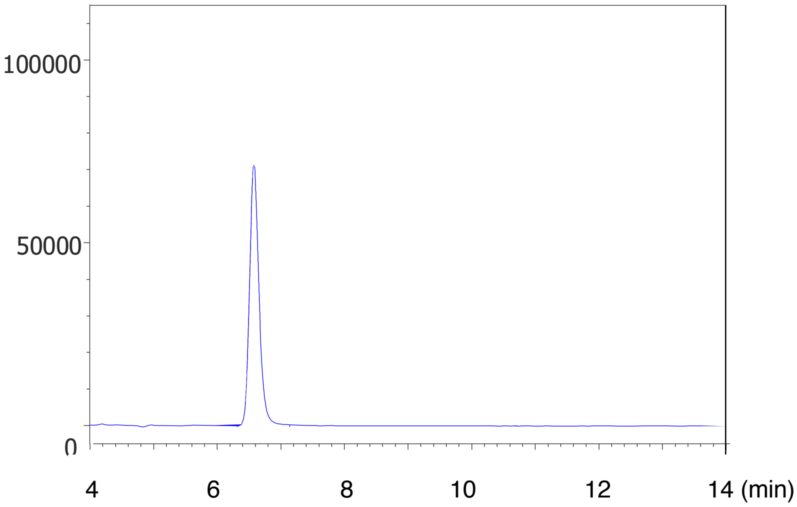

HPLC analysis of **2*S*-4**

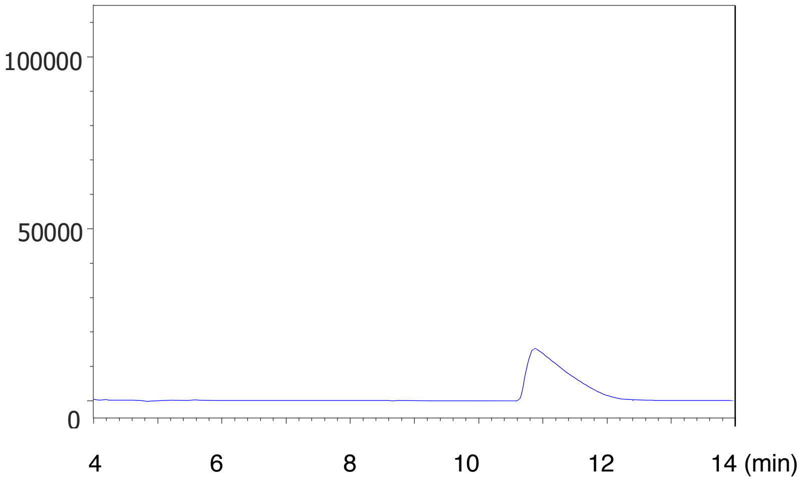

HPLC analysis of **2*R*-4**

3. NMR Spectra

^{1}H NMR Spectrum of **3a** ($CDCl_3$, 400 MHz).

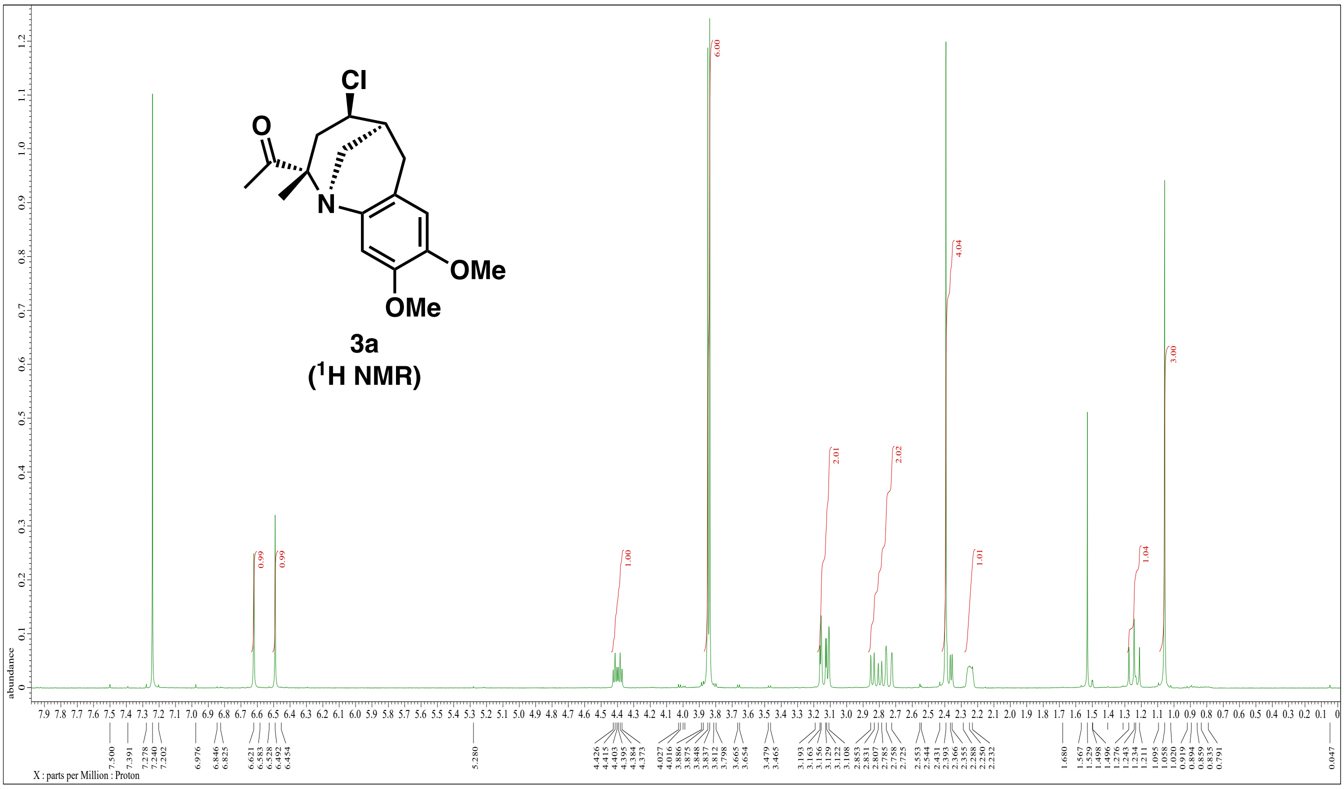

^{13}C{^{1}H} NMR Spectrum of **3a** ($CDCl_3$, 100 MHz).

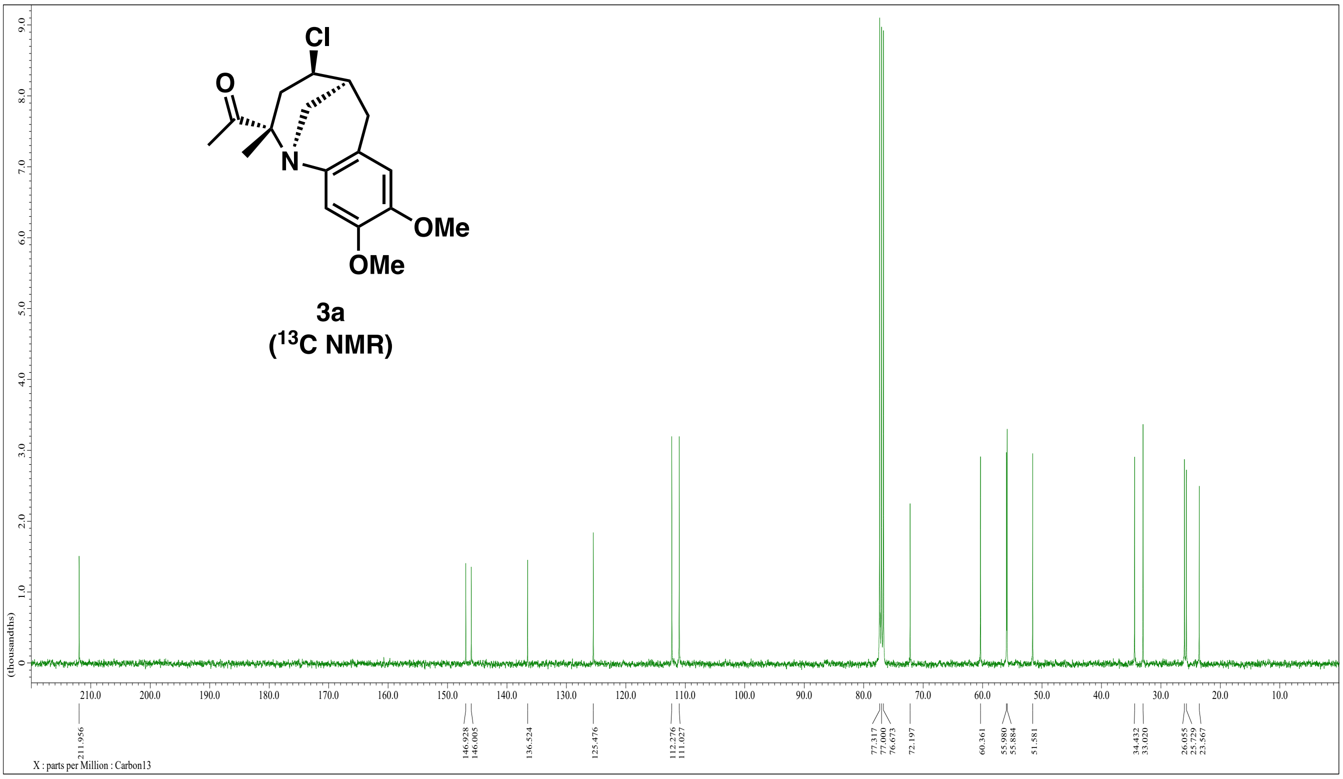

^{1}H NMR Spectrum of **3b** ($CDCl_3$, 400 MHz).

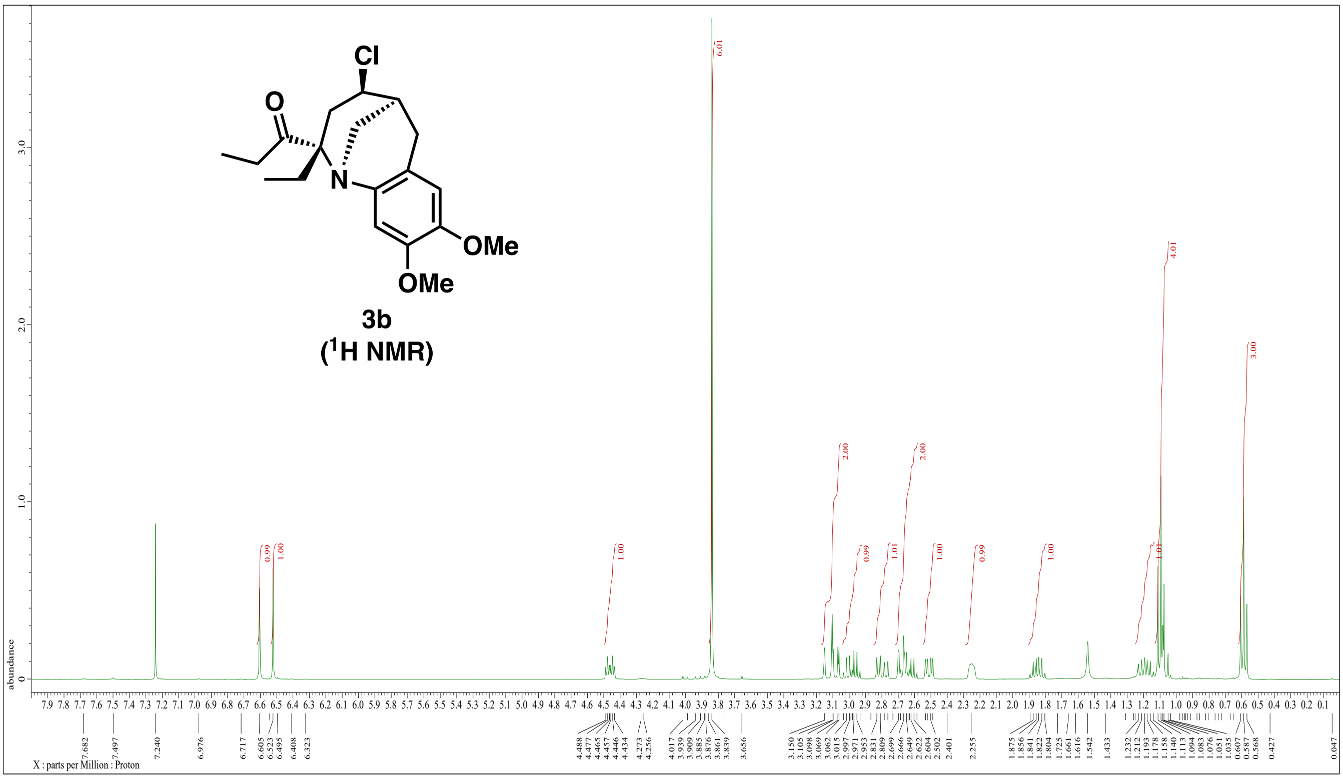

$^{13}C\{^{1}H\}$ NMR Spectrum of **3b** ($CDCl_3$, 100 MHz).

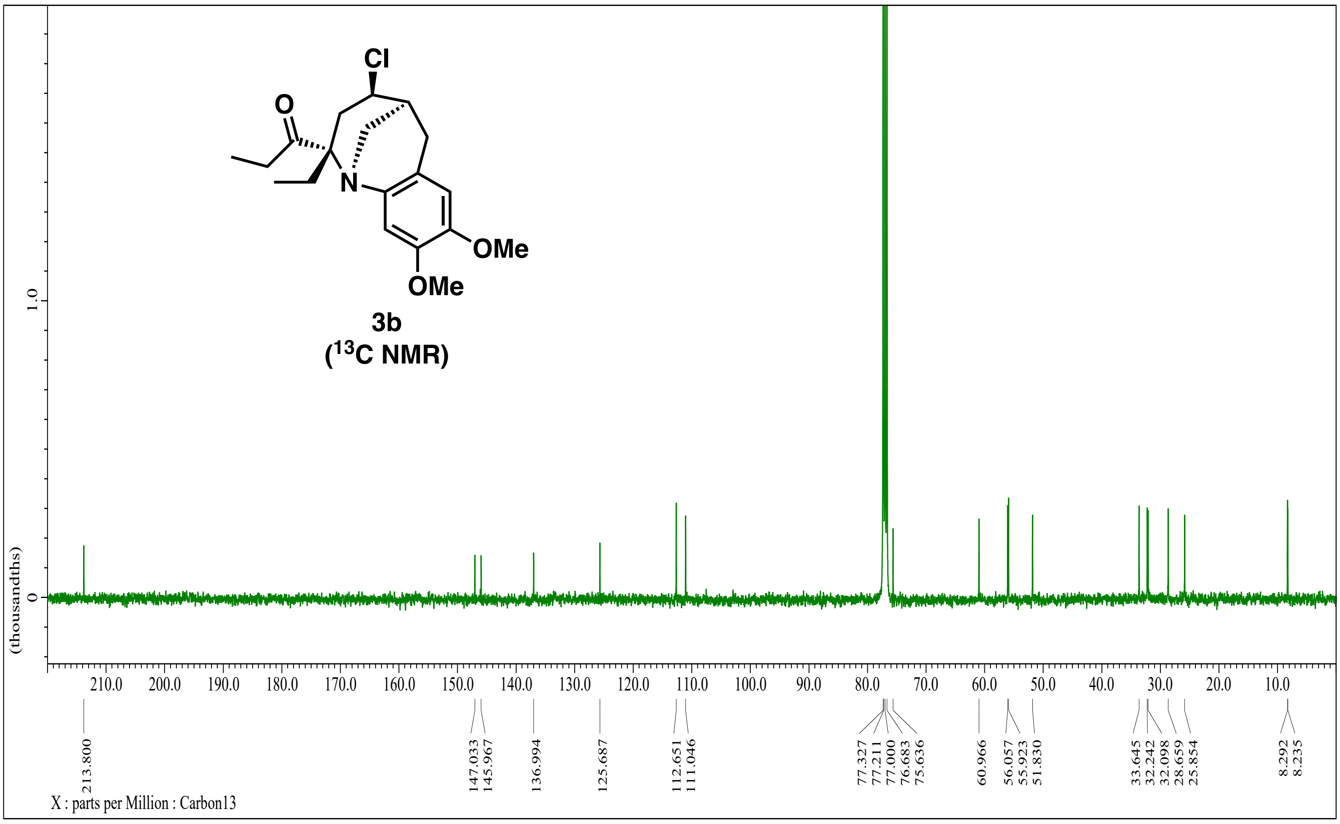

^{1}H NMR Spectrum of **3c** ($CDCl_3$, 400 MHz).

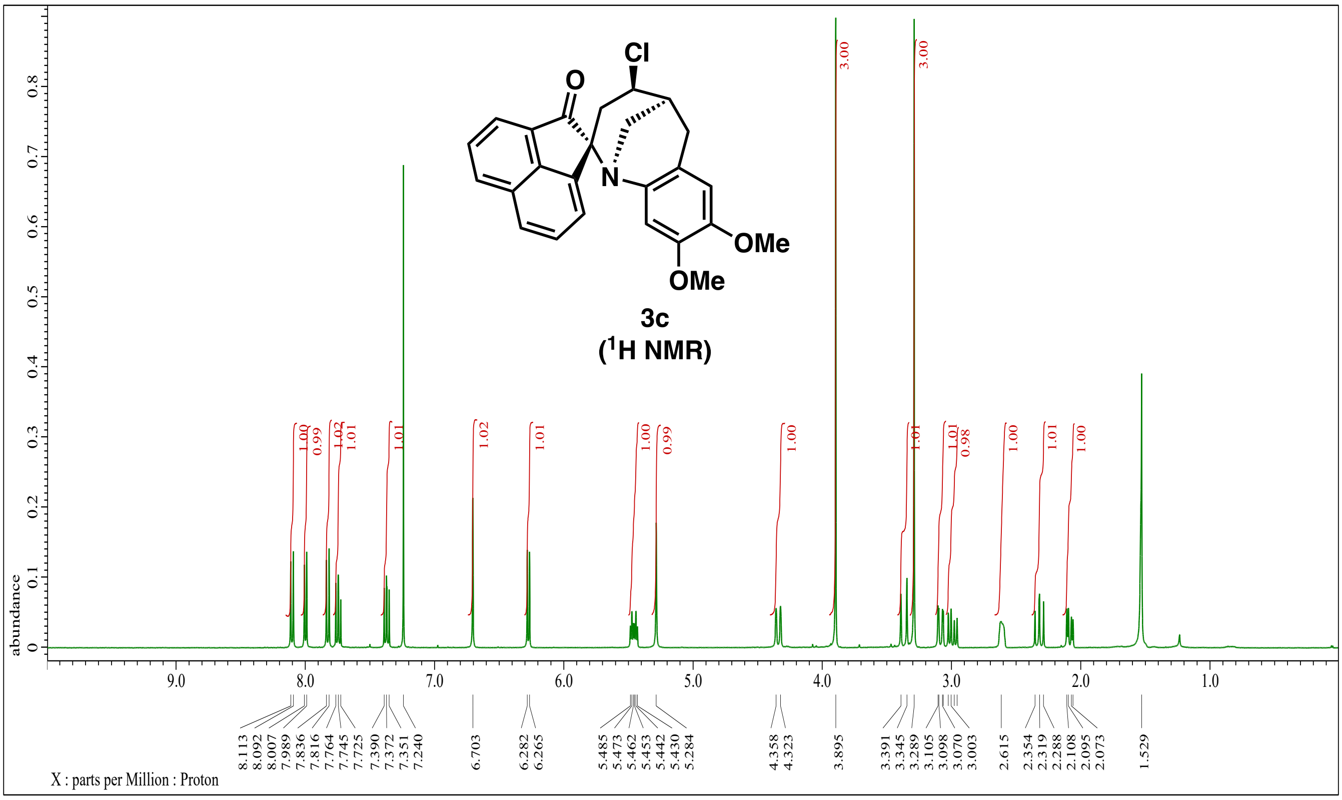

^{13}C{^{1}H} NMR Spectrum of **3c** ($CDCl_3$, 100 MHz).

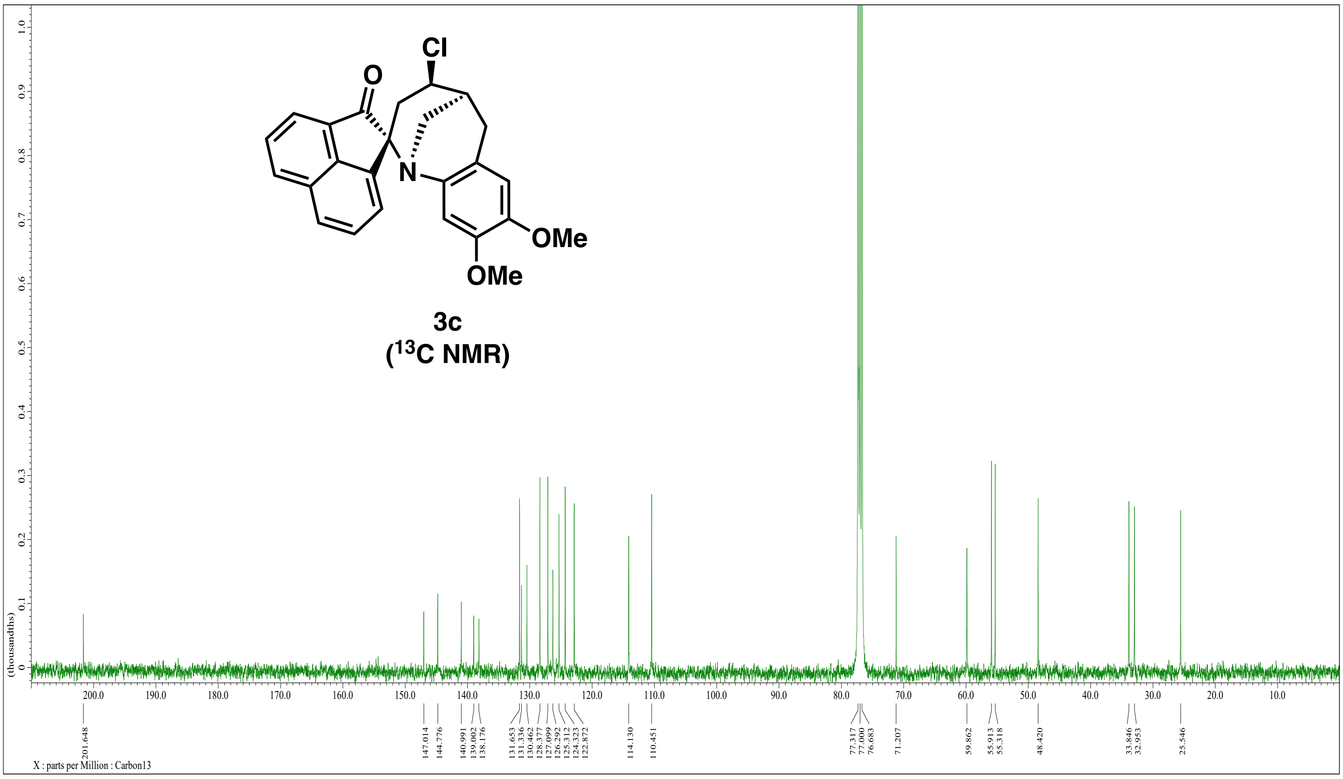

^{1}H NMR Spectrum of **3d** (CDCl$_3$, 400 MHz).

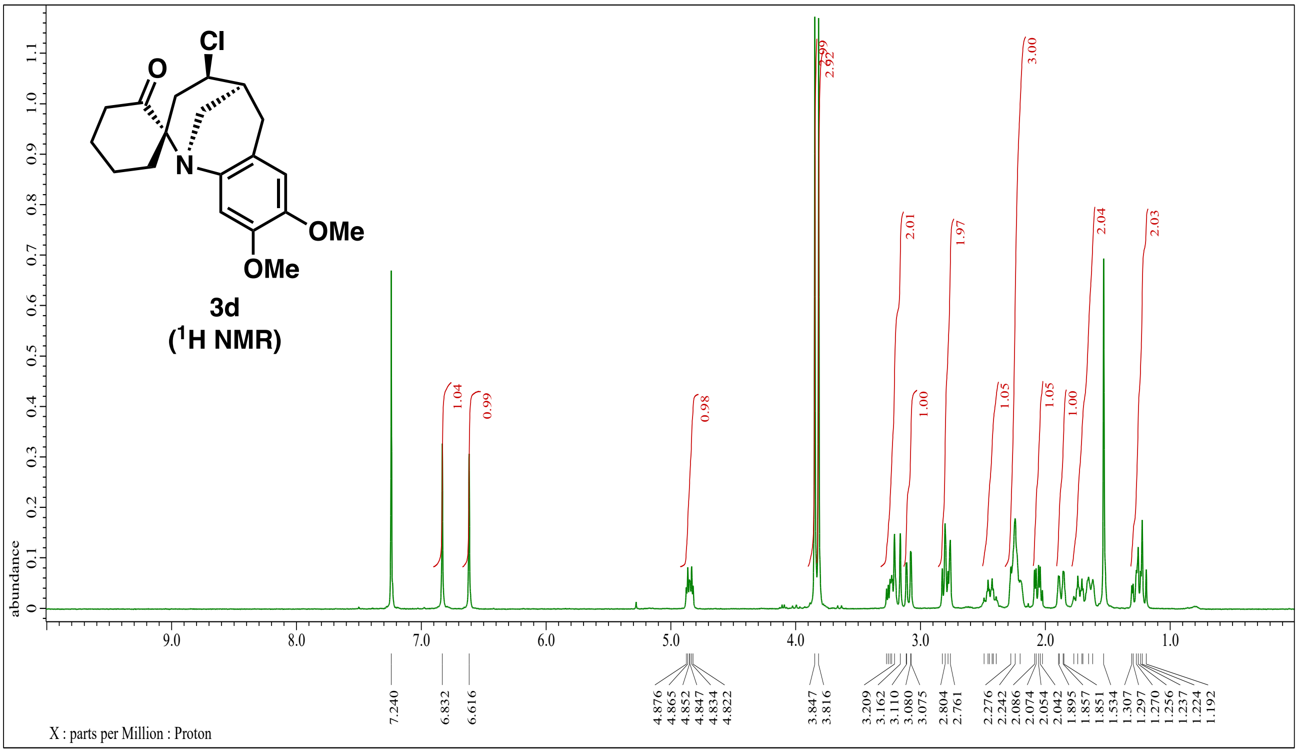

^{13}C{^{1}H} NMR Spectrum of **3d** (CDCl$_3$, 100 MHz).

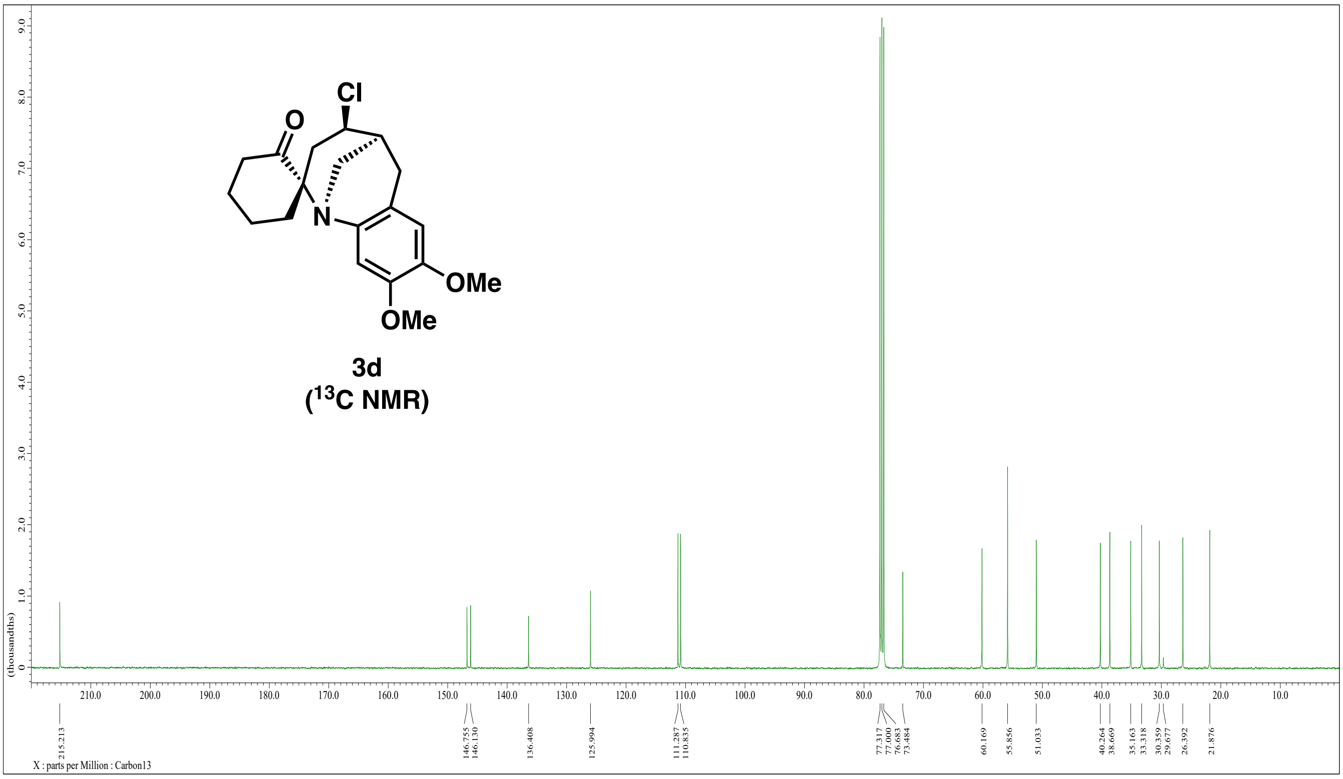

^{1}H NMR Spectrum of **3e** ($CDCl_3$, 400 MHz).

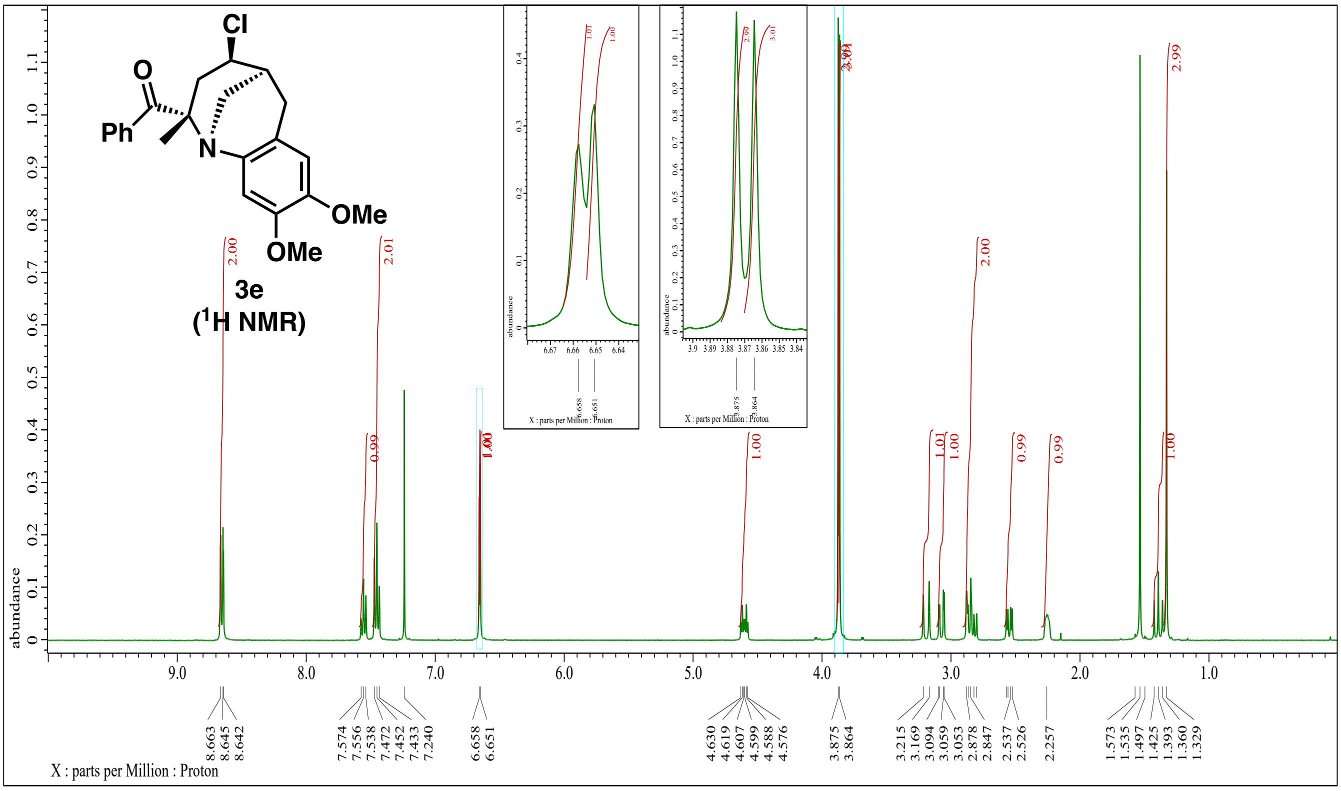

^{13}C{^{1}H} NMR Spectrum of **3e** ($CDCl_3$, 100 MHz).

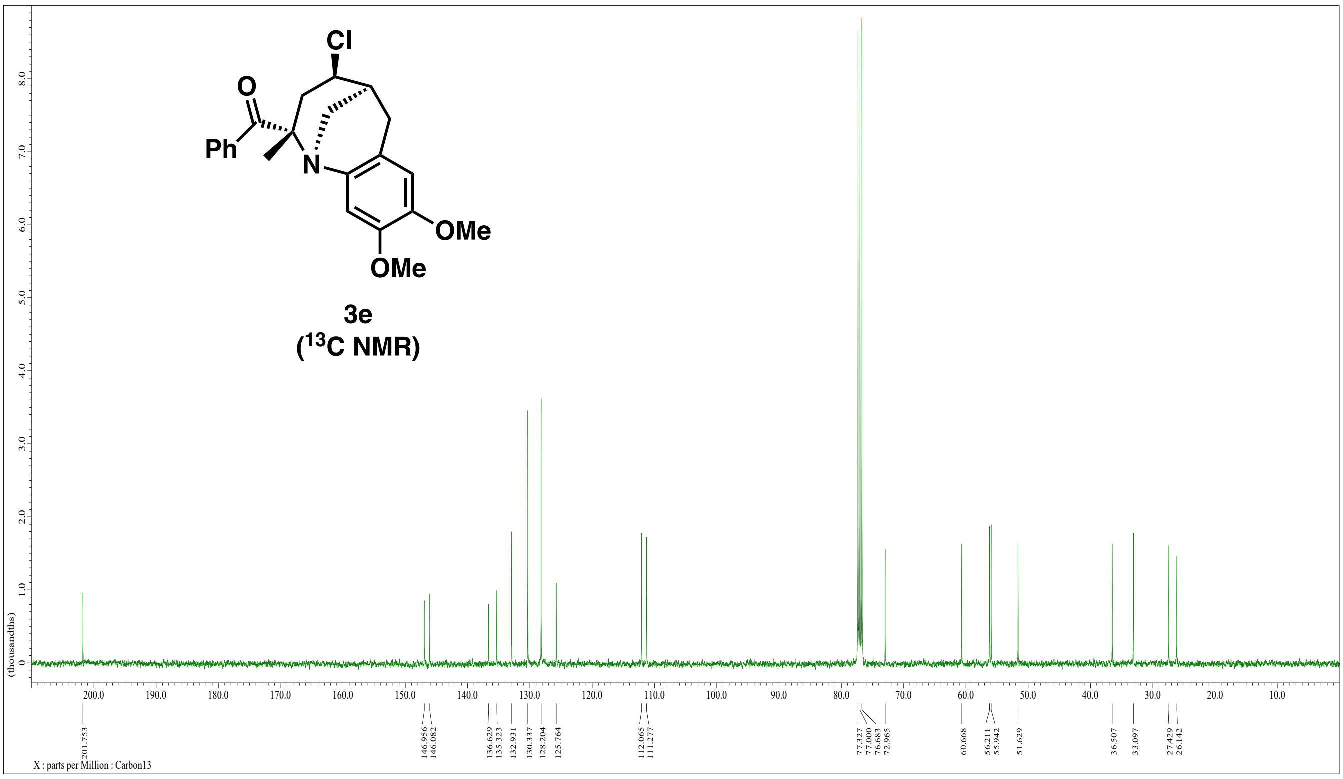

^{1}H NMR Spectrum of **3f** ($CDCl_3$, 400 MHz).

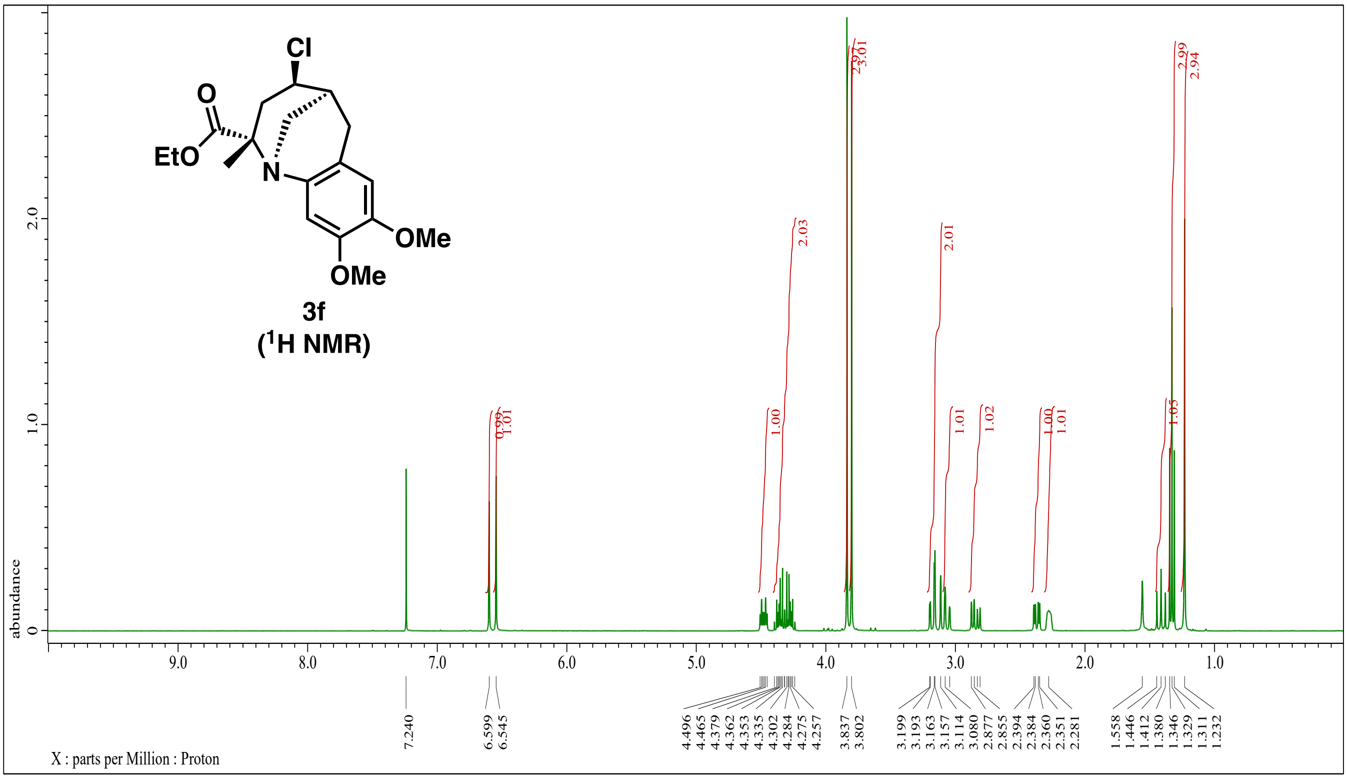

^{13}C{^{1}H} NMR Spectrum of **3f** ($CDCl_3$, 100 MHz).

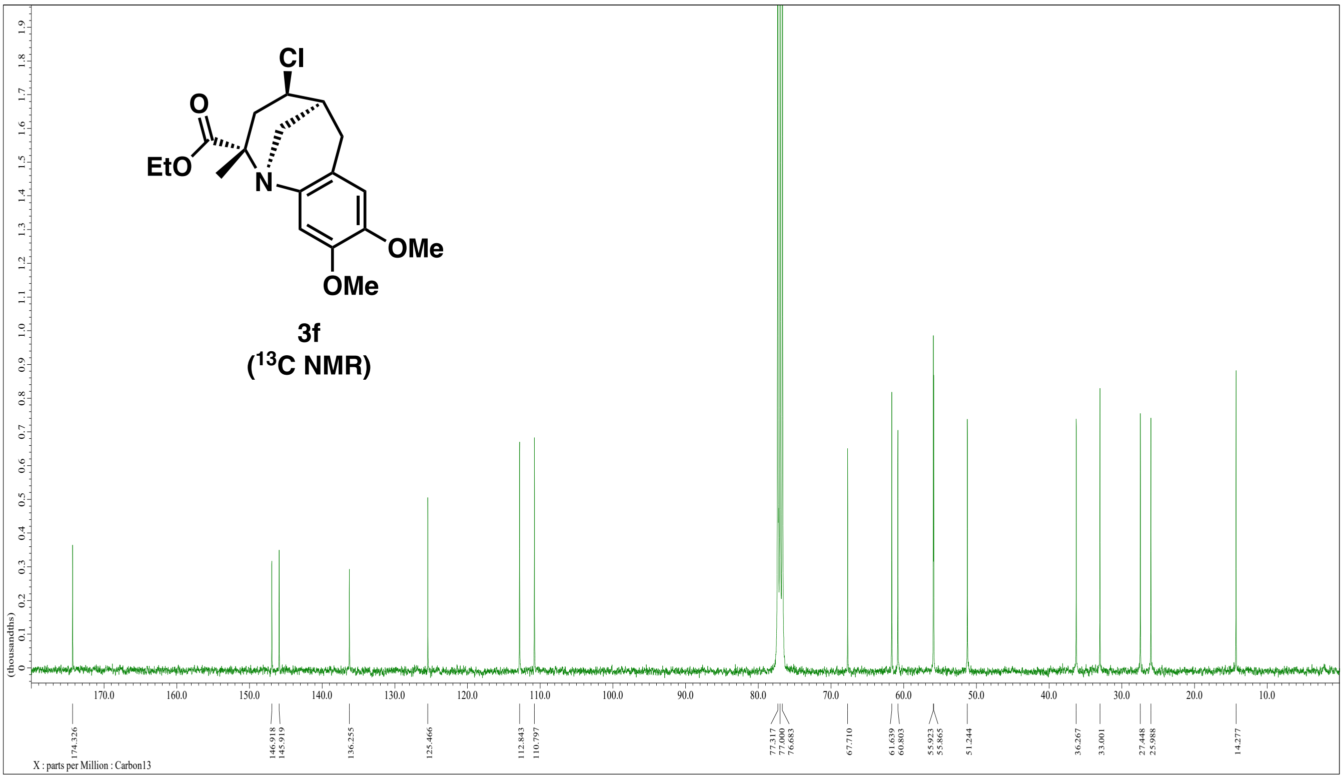

^{1}H NMR Spectrum of **3g** ($CDCl_3$, 400 MHz).

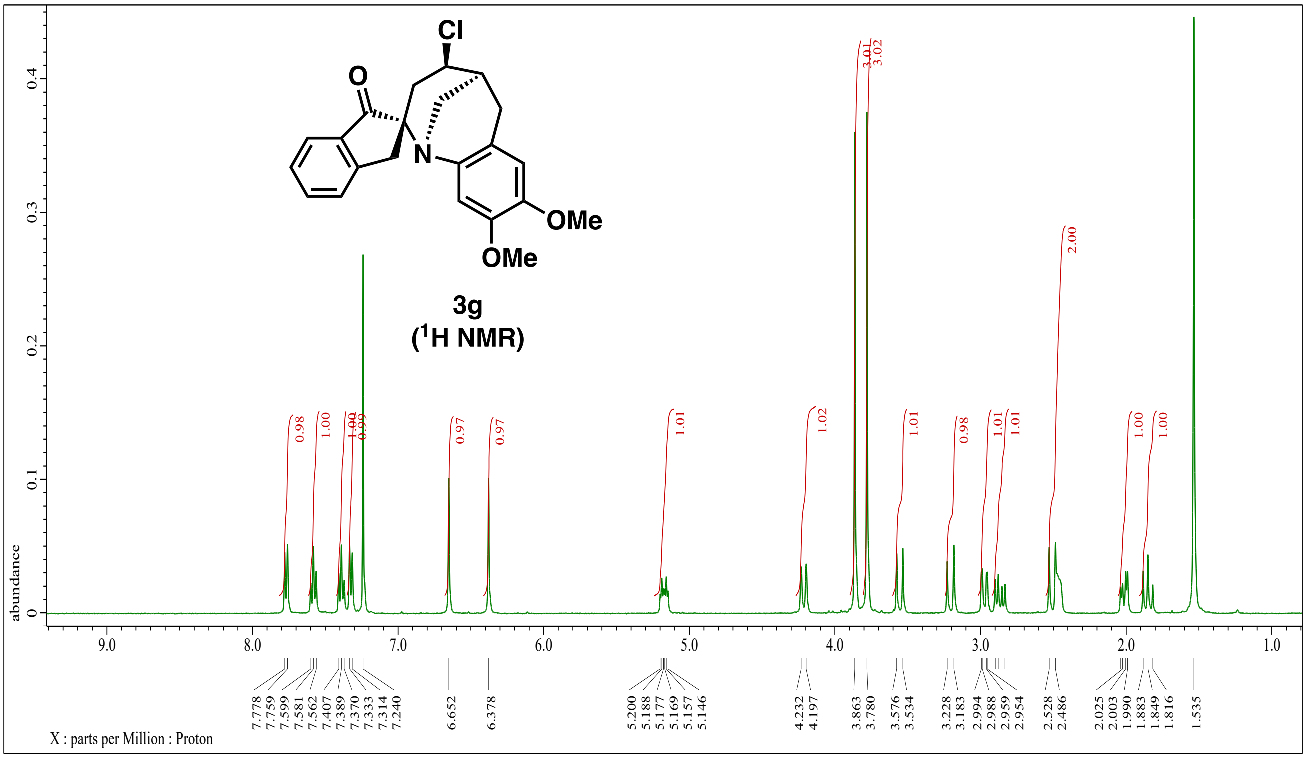

^{13}C{^{1}H} NMR Spectrum of **3g** ($CDCl_3$, 126 MHz).

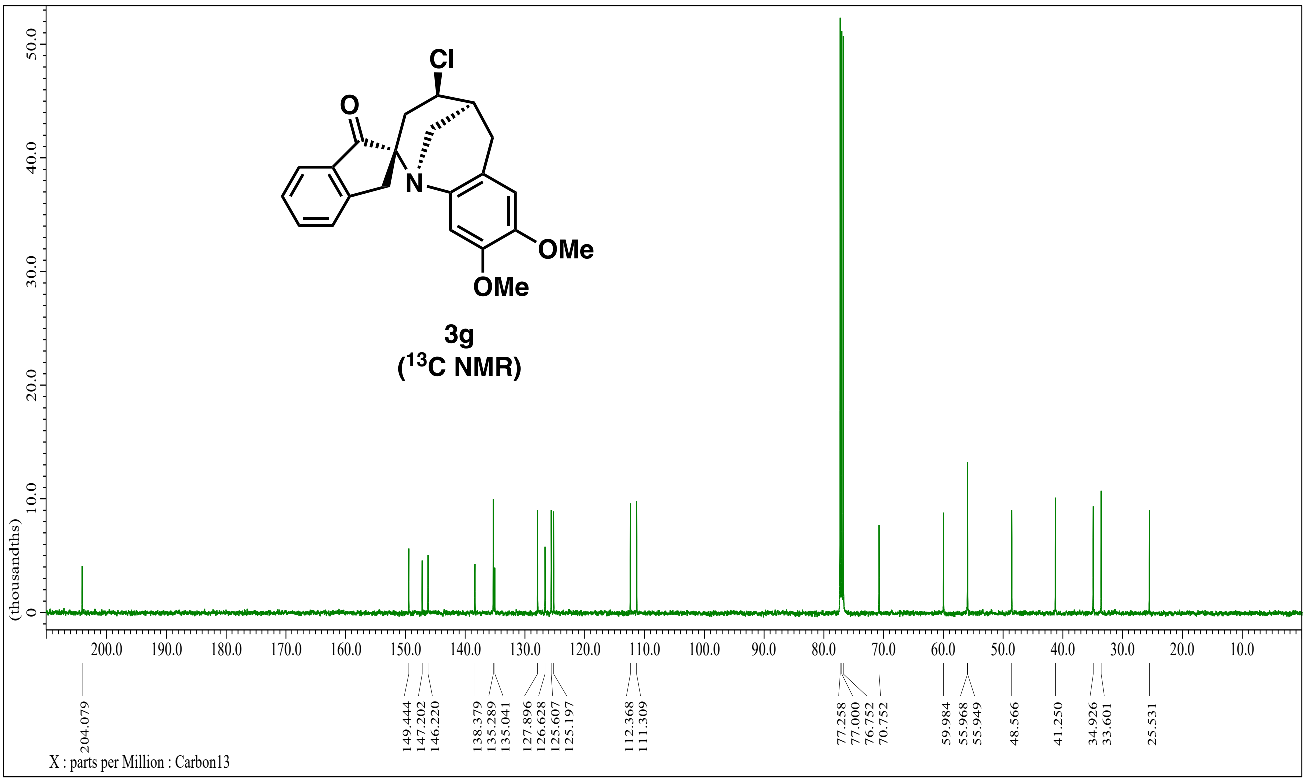

^{1}H NMR Spectrum of **3h** ($CDCl_3$, 400 MHz).

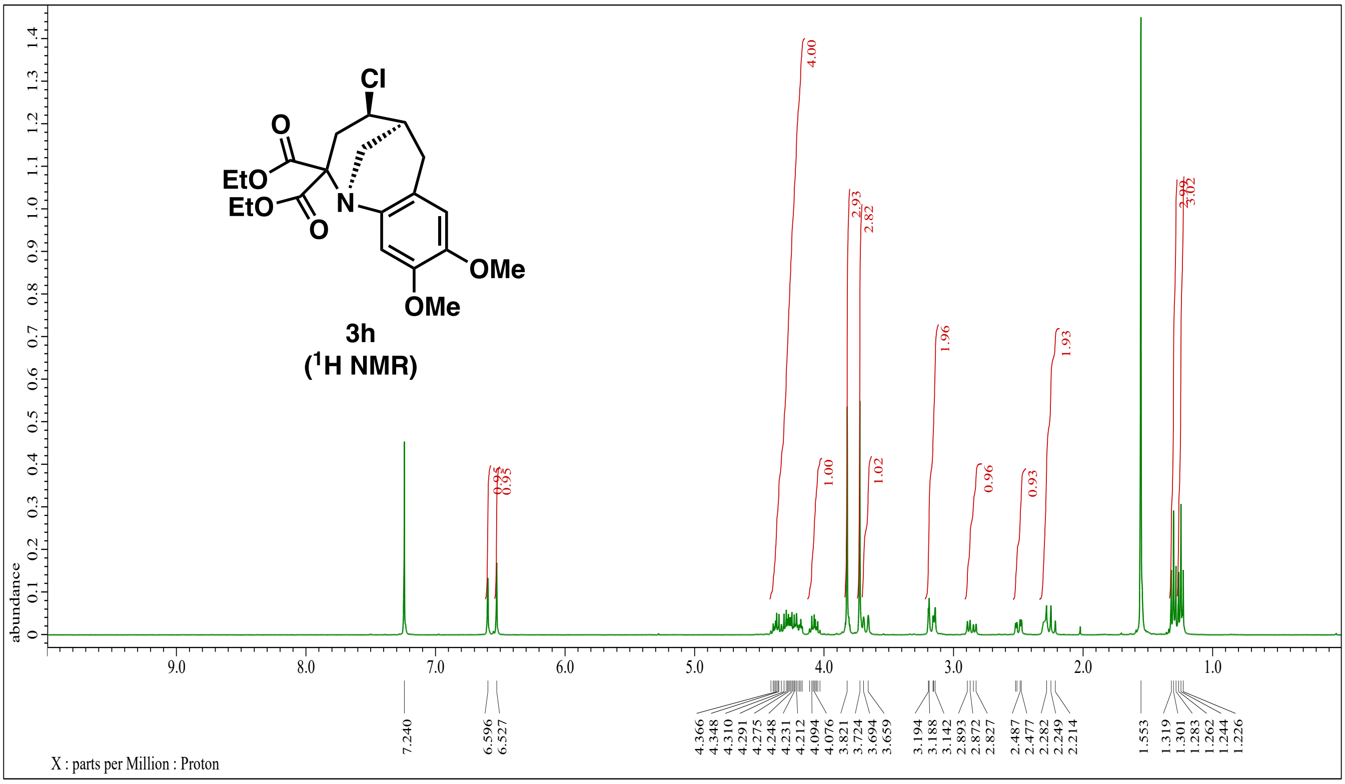

^{13}C{^{1}H} NMR Spectrum of **3h** ($CDCl_3$, 100 MHz).

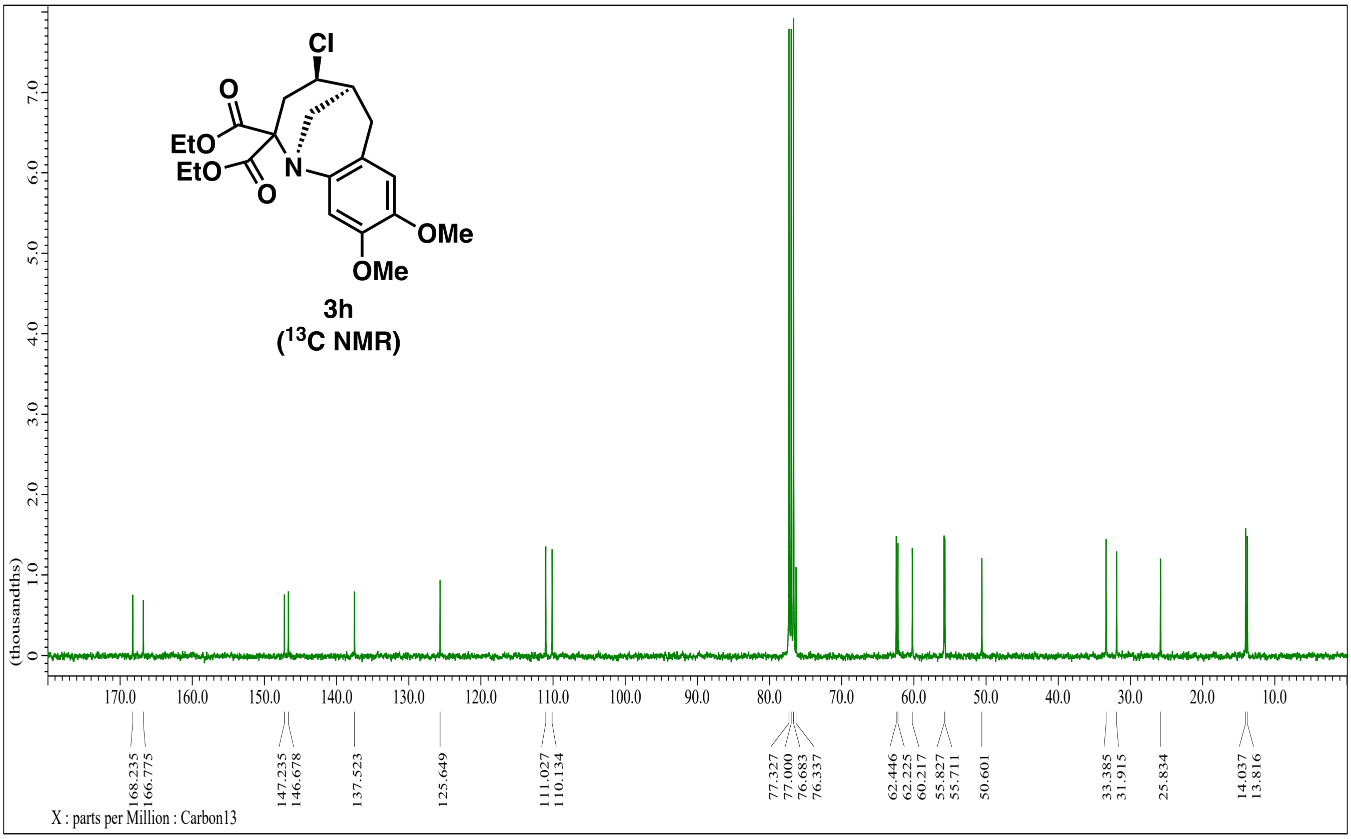

^{1}H NMR Spectrum of **2i** ($CDCl_3$, 400 MHz).

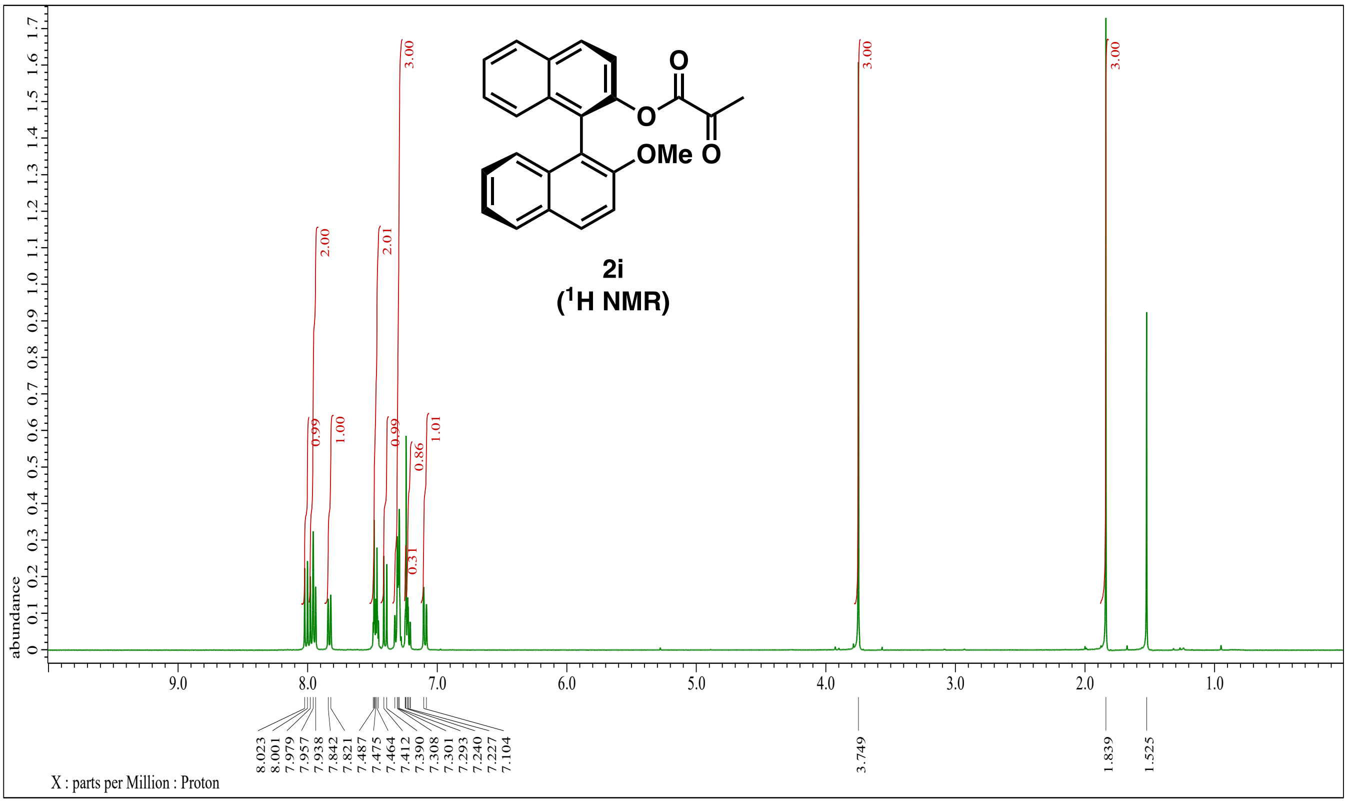

^{13}C{^{1}H} NMR Spectrum of **2i** ($CDCl_3$, 100 MHz).

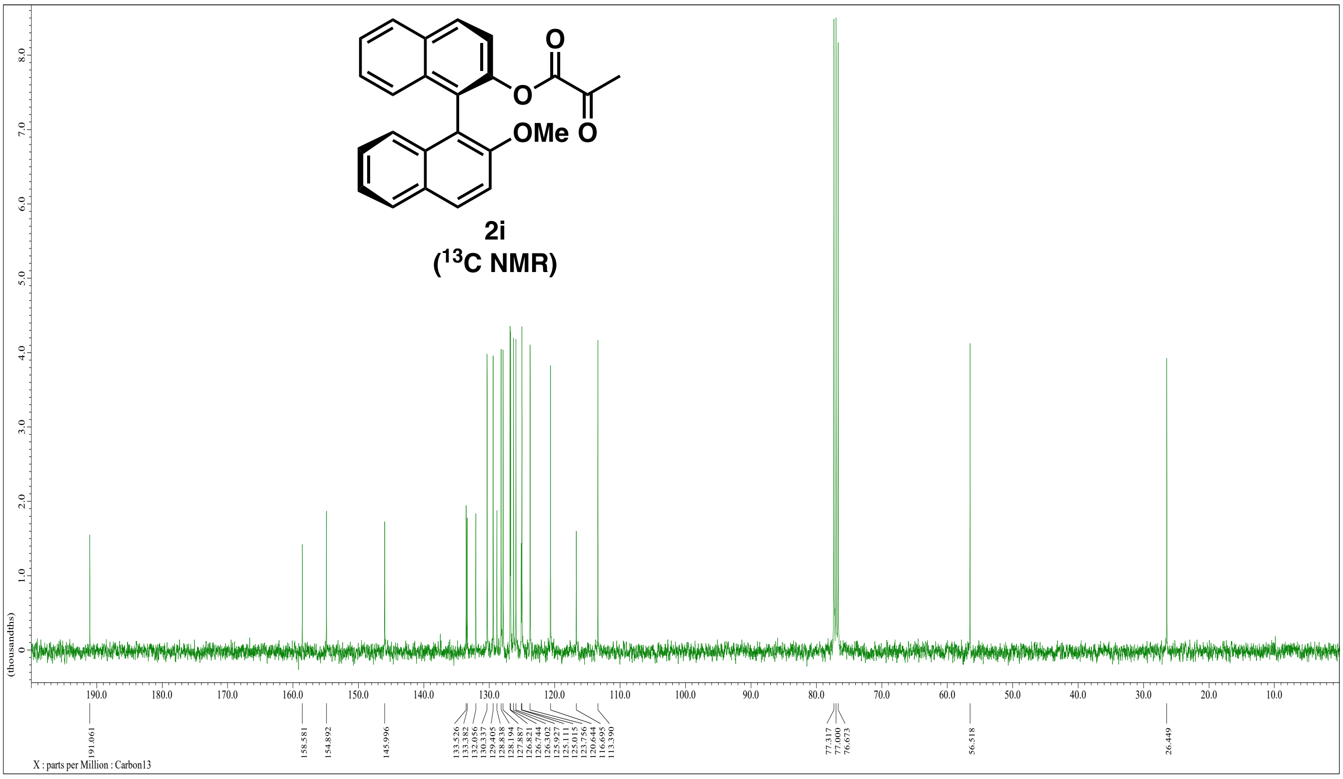

^{1}H NMR Spectrum of **2*S*-3i** ($CDCl_3$, 400 MHz).

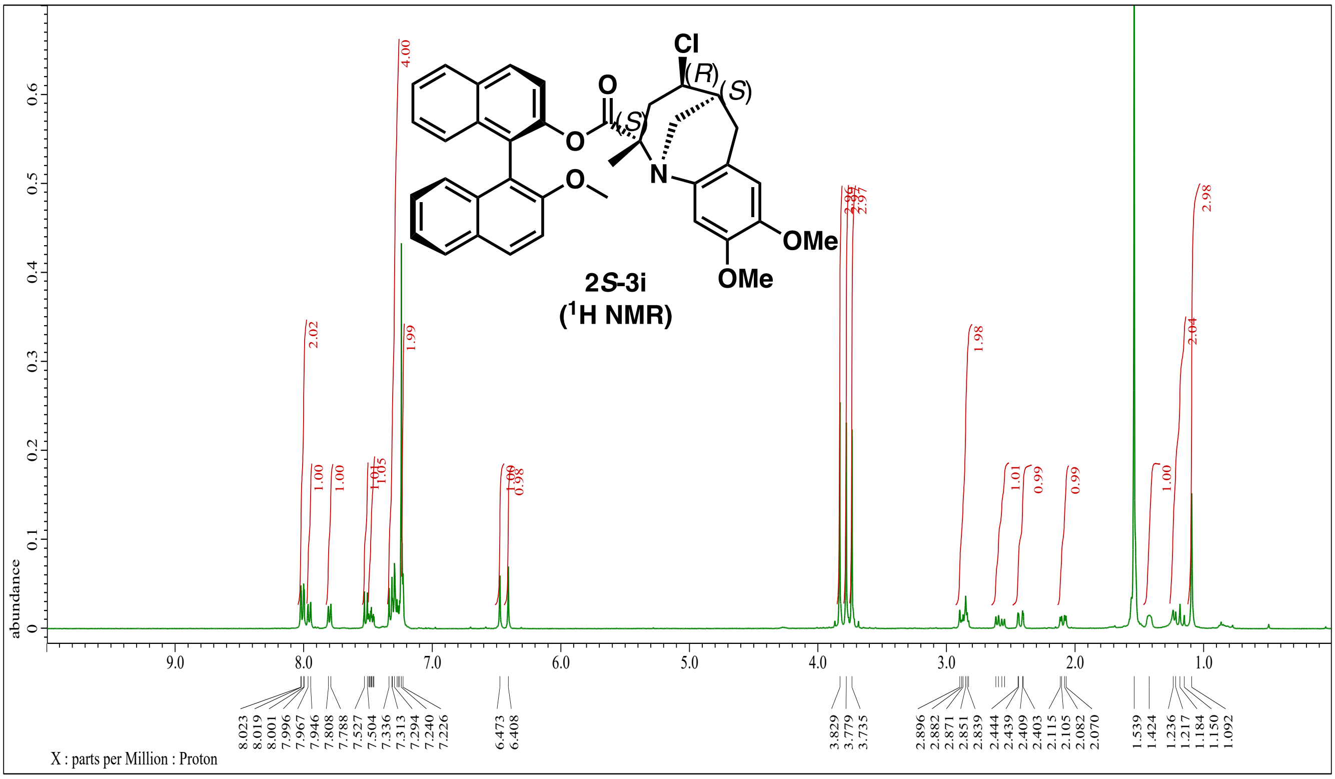

^{13}C{^{1}H} NMR Spectrum of **2*S*-3g** ($CDCl_3$, 126 MHz).

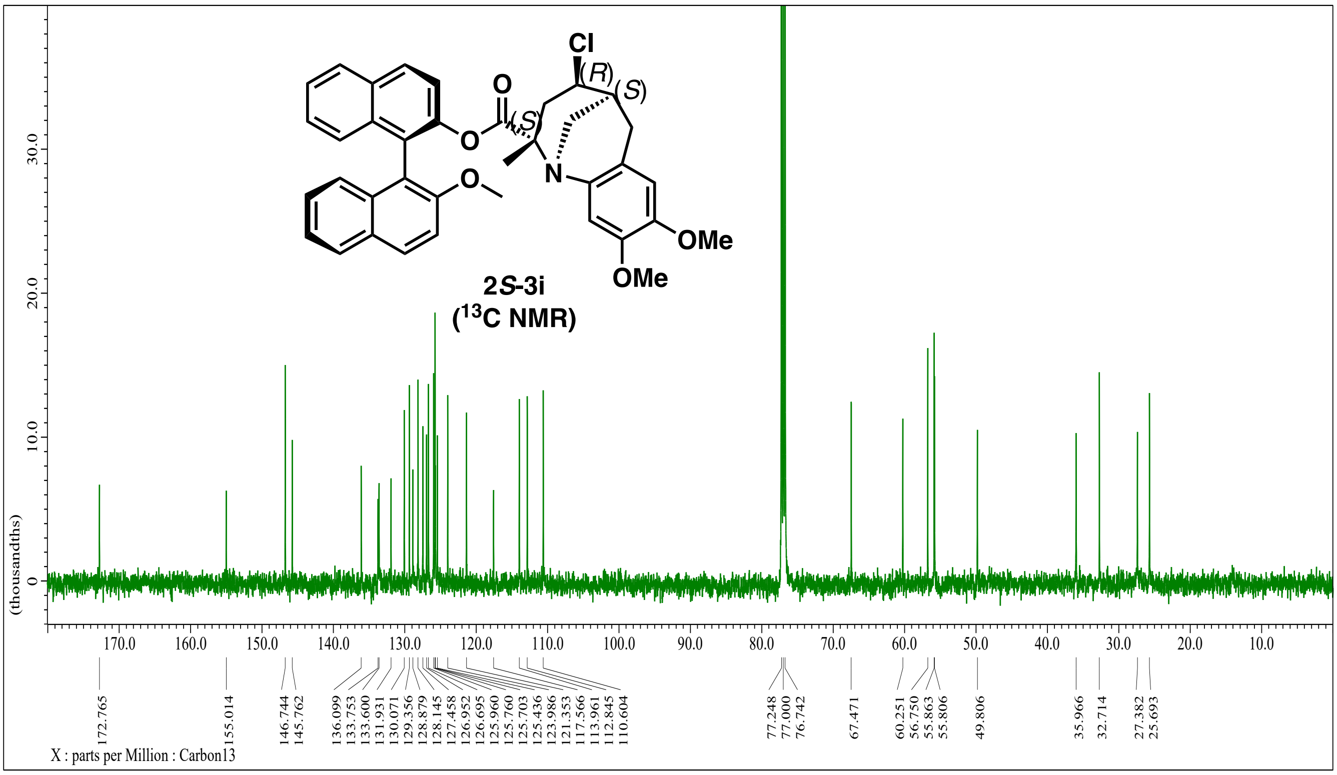

^{1}H NMR Spectrum of **2*S*-3i** (CD_2Cl_2, 400 MHz)**.** This spectrum was recorded to confirm the integration of the aromatic signals.

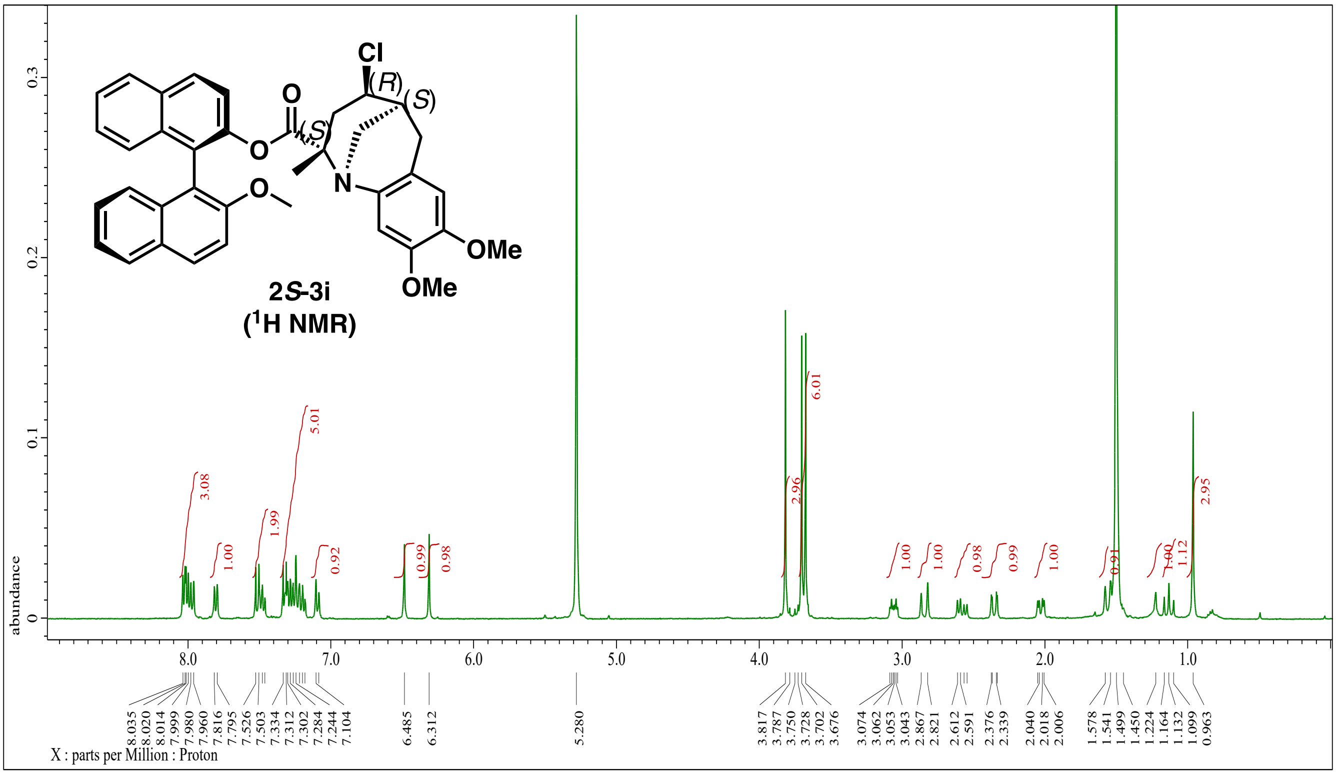

^{1}H NMR Spectrum of **2*R*-3i** ($CDCl_3$, 400 MHz).

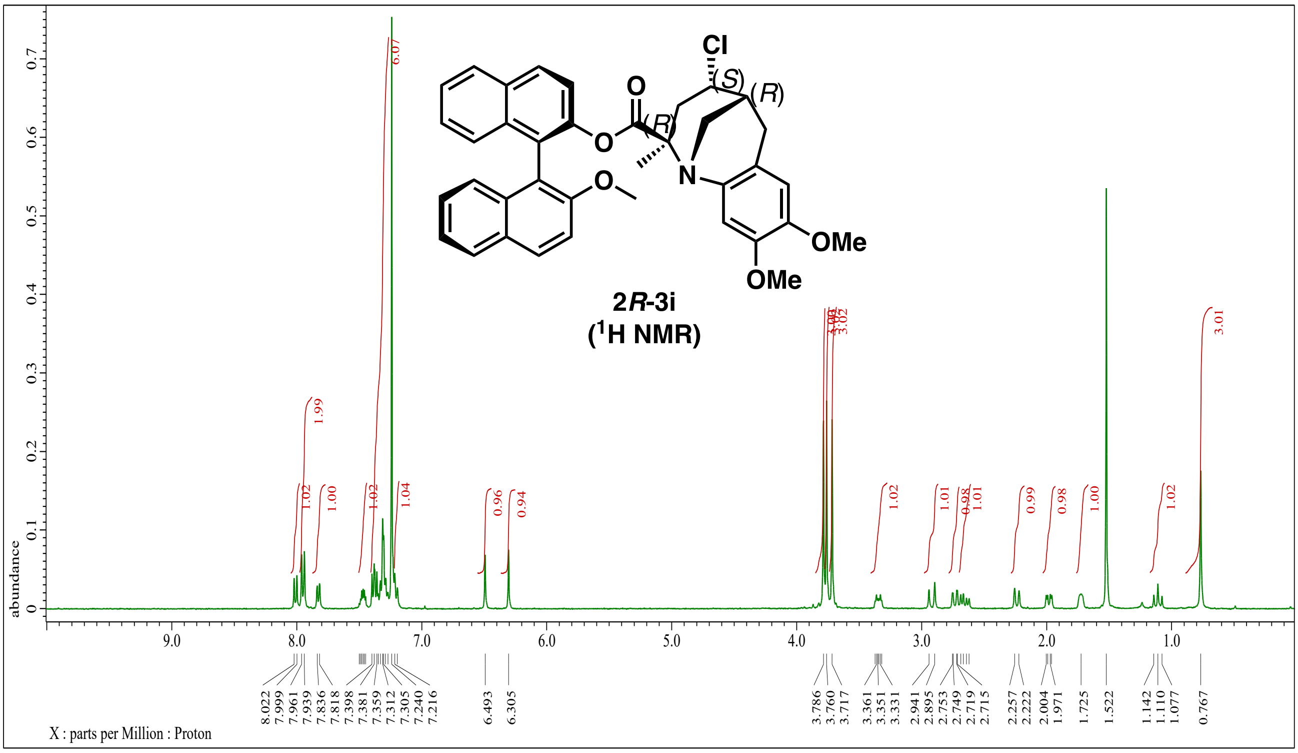

^{13}C{^{1}H} NMR Spectrum of **2*R*-3i** ($CDCl_3$, 126 MHz).

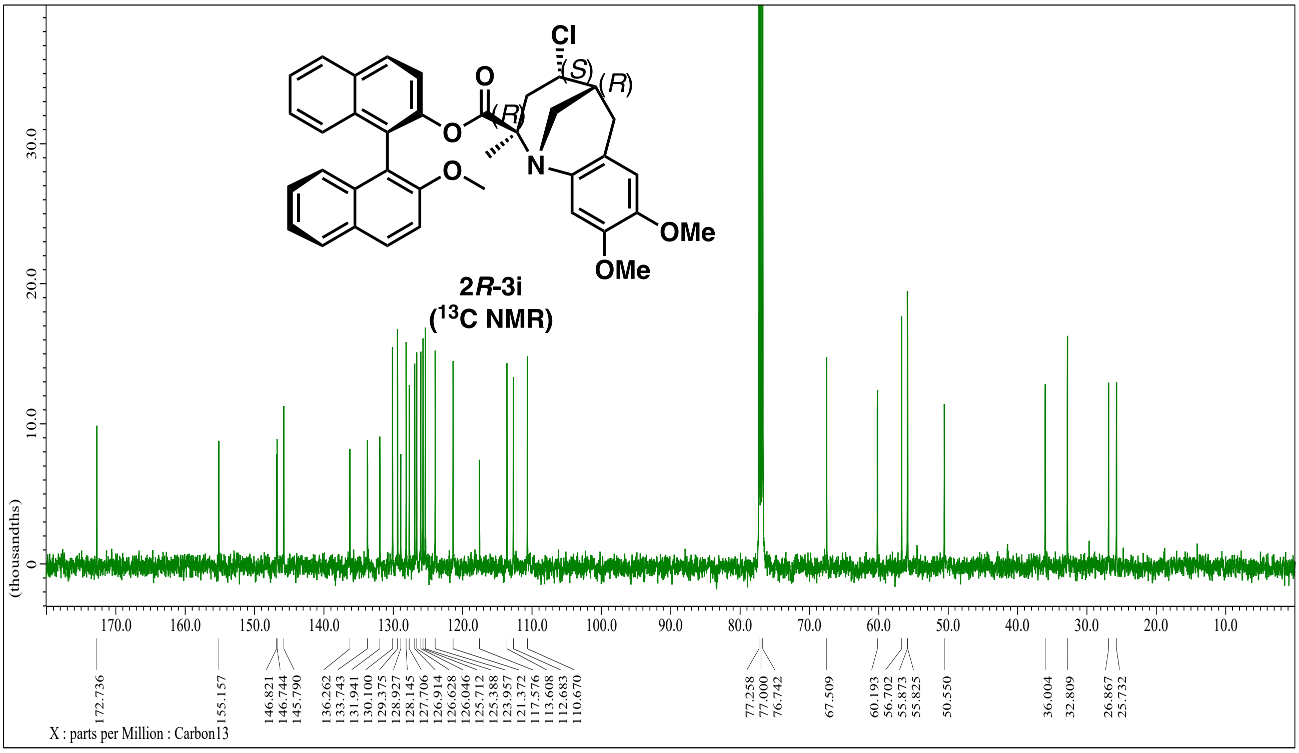

^{1}H NMR Spectrum of ***rac*-4** ($CDCl_3$, 400 MHz).

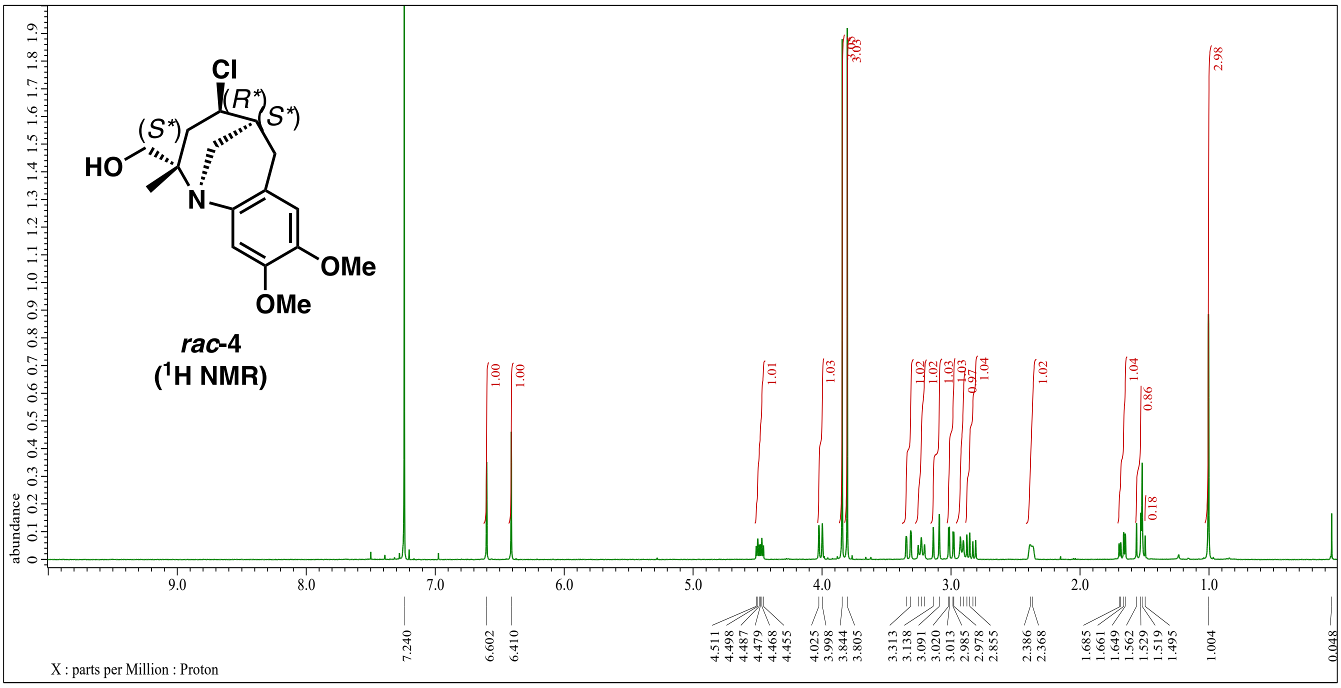

^{13}C{^{1}H} NMR Spectrum of ***rac*-4** ($CDCl_3$, 100 MHz).

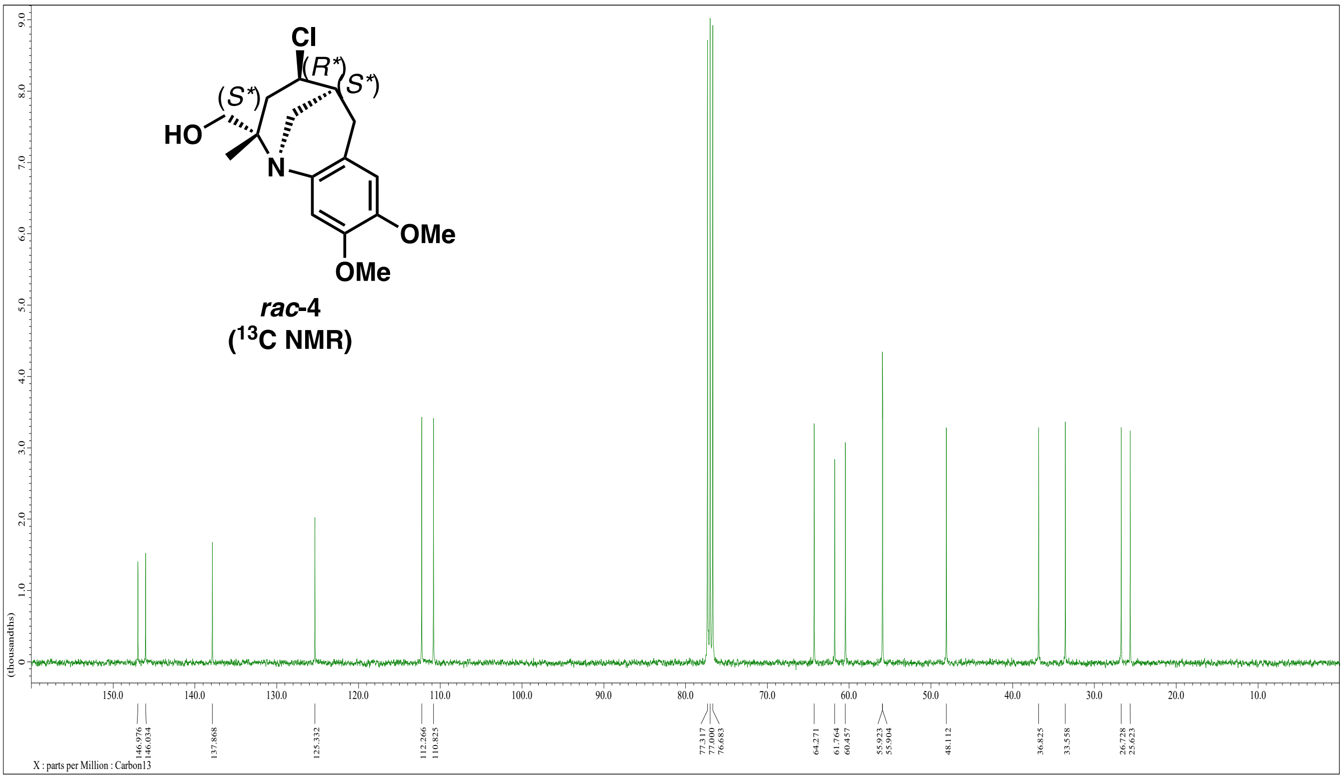

^{1}H NMR Spectrum of **6a** ($CDCl_3$, 400 MHz).

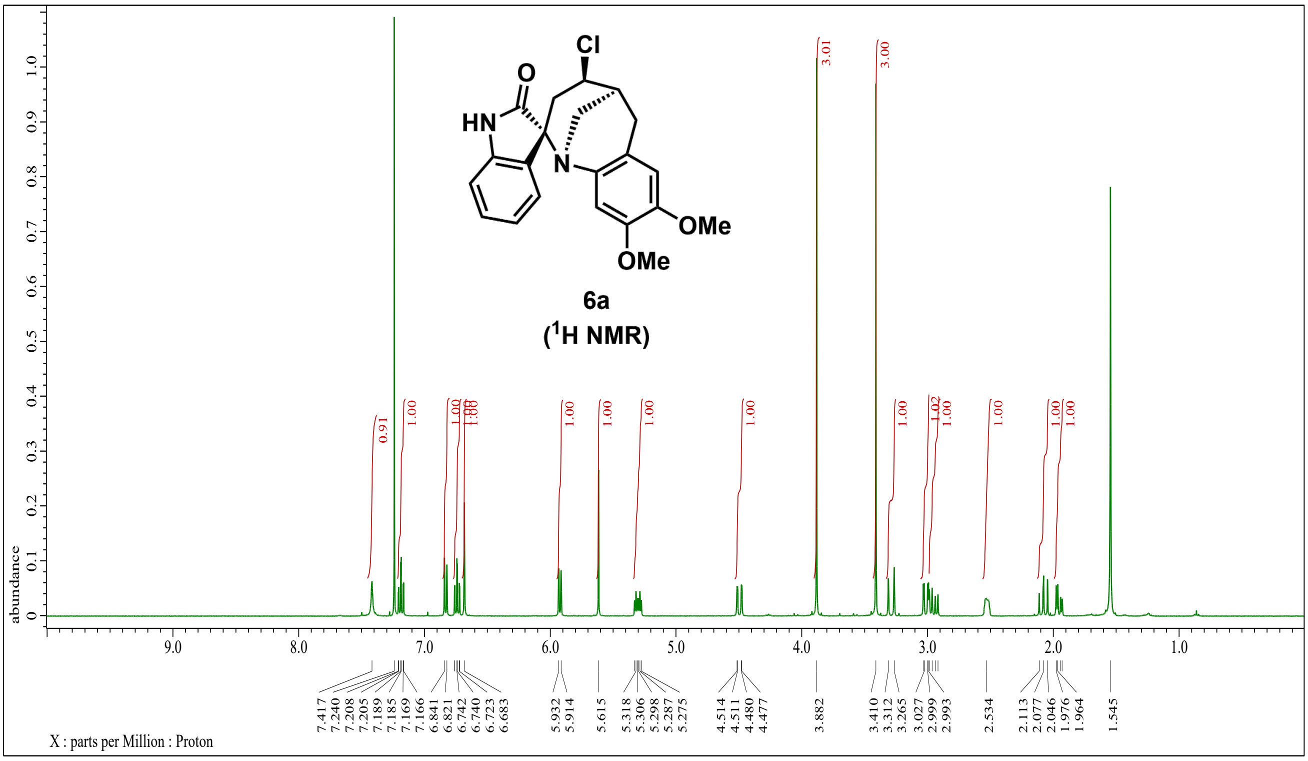

^{13}C{^{1}H} NMR Spectrum of **6a** ($CDCl_3$, 100 MHz).

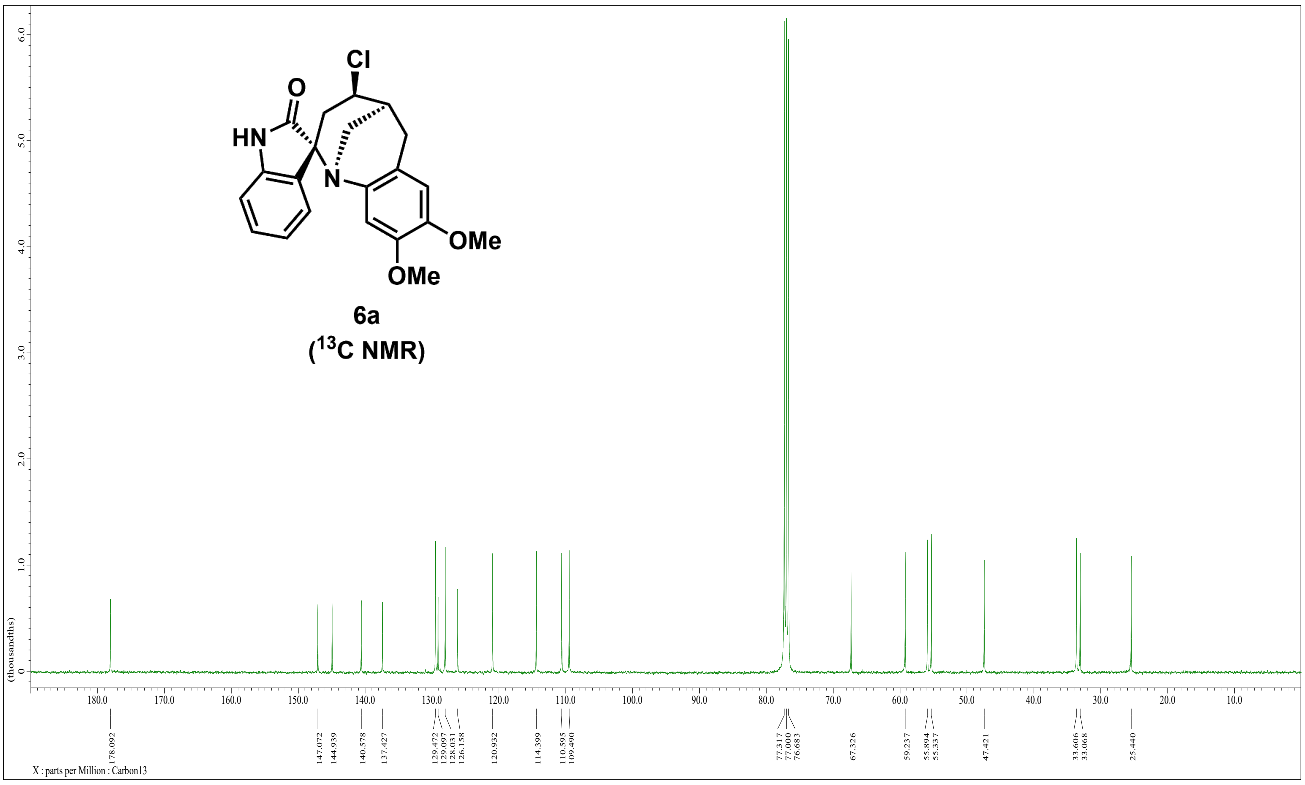

^{1}H NMR Spectrum of **6b** ($CDCl_3$, 400 MHz).

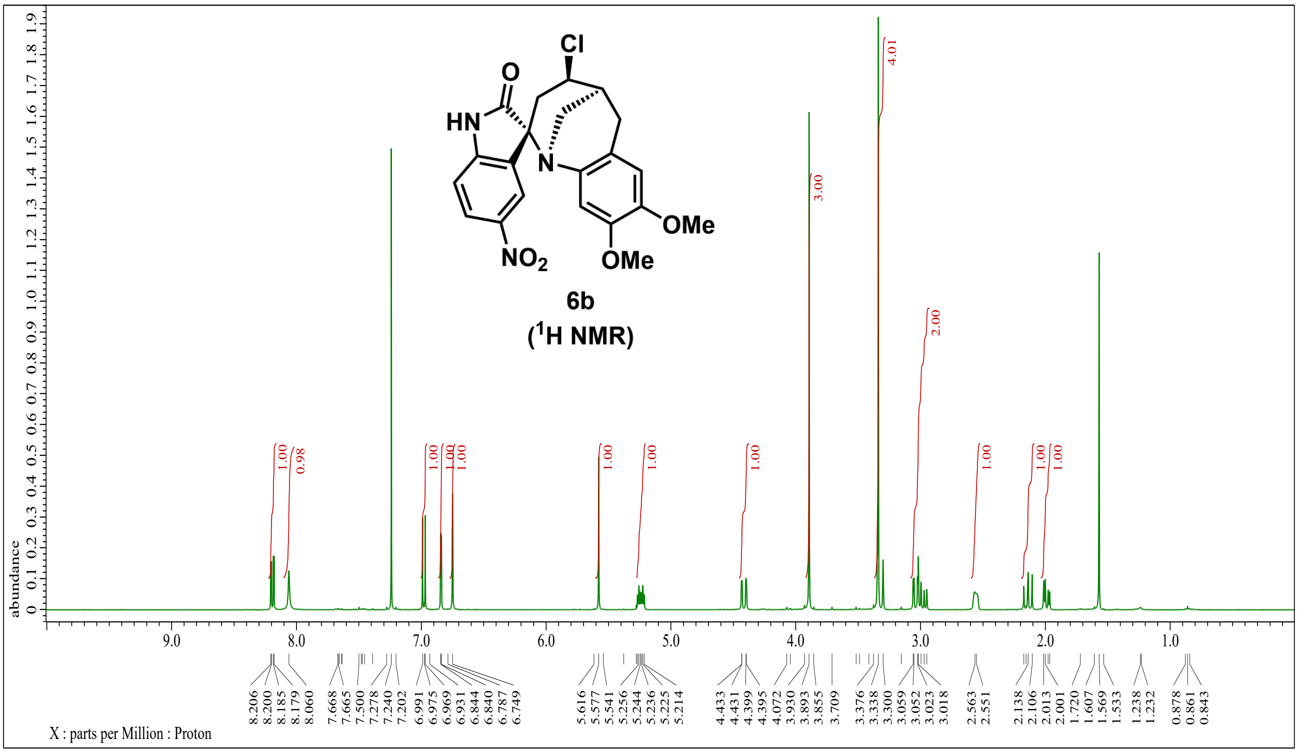

^{13}C{^{1}H} NMR Spectrum of **6b** ($CDCl_3$, 100 MHz).

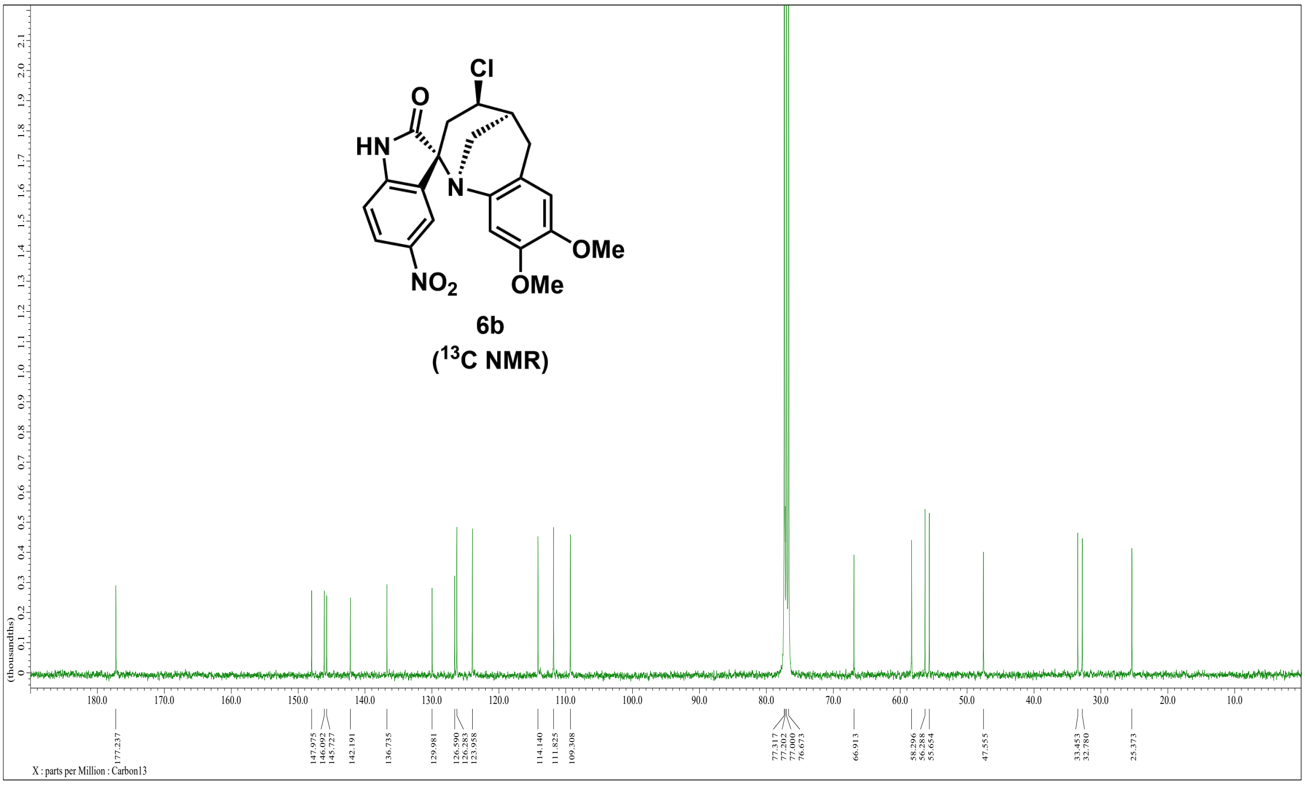

^{1}H NMR Spectrum of **6c** ($CDCl_3$, 400 MHz).

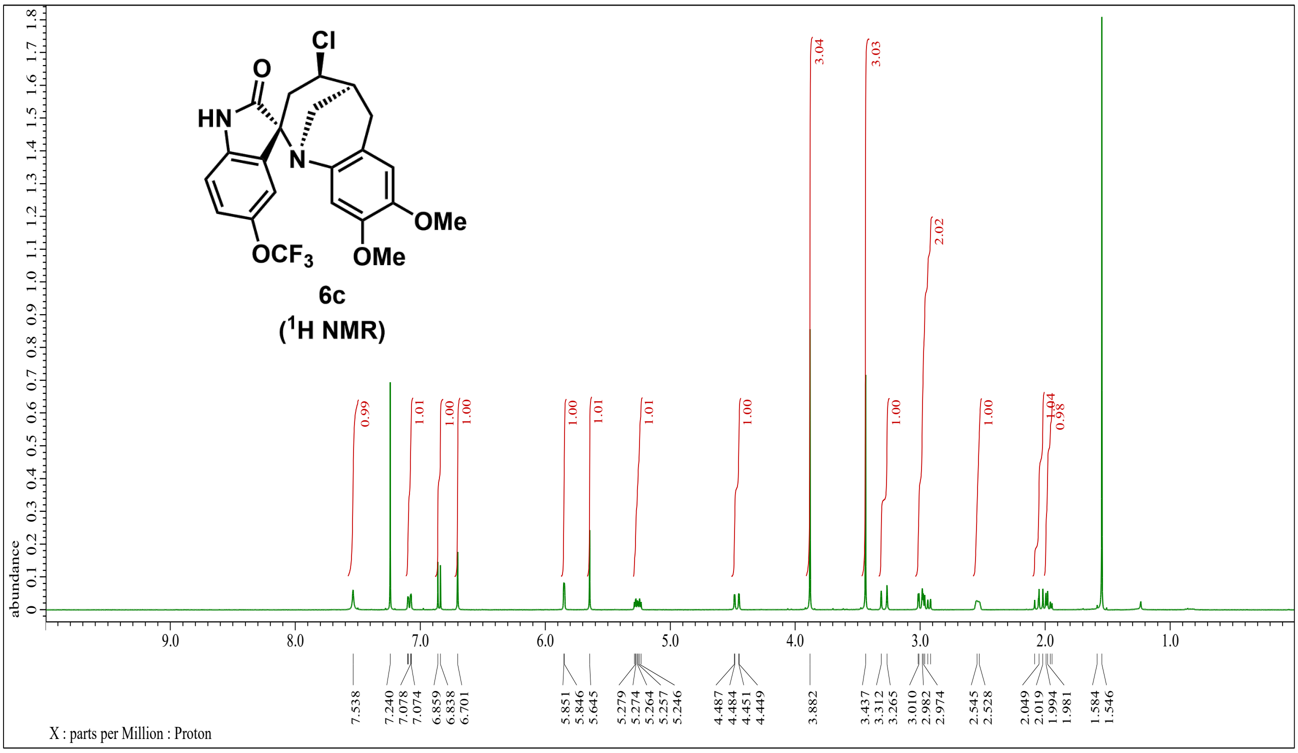

$^{13}C\{^{1}H\}$ NMR Spectrum of **6c** ($CDCl_3$, 100 MHz).

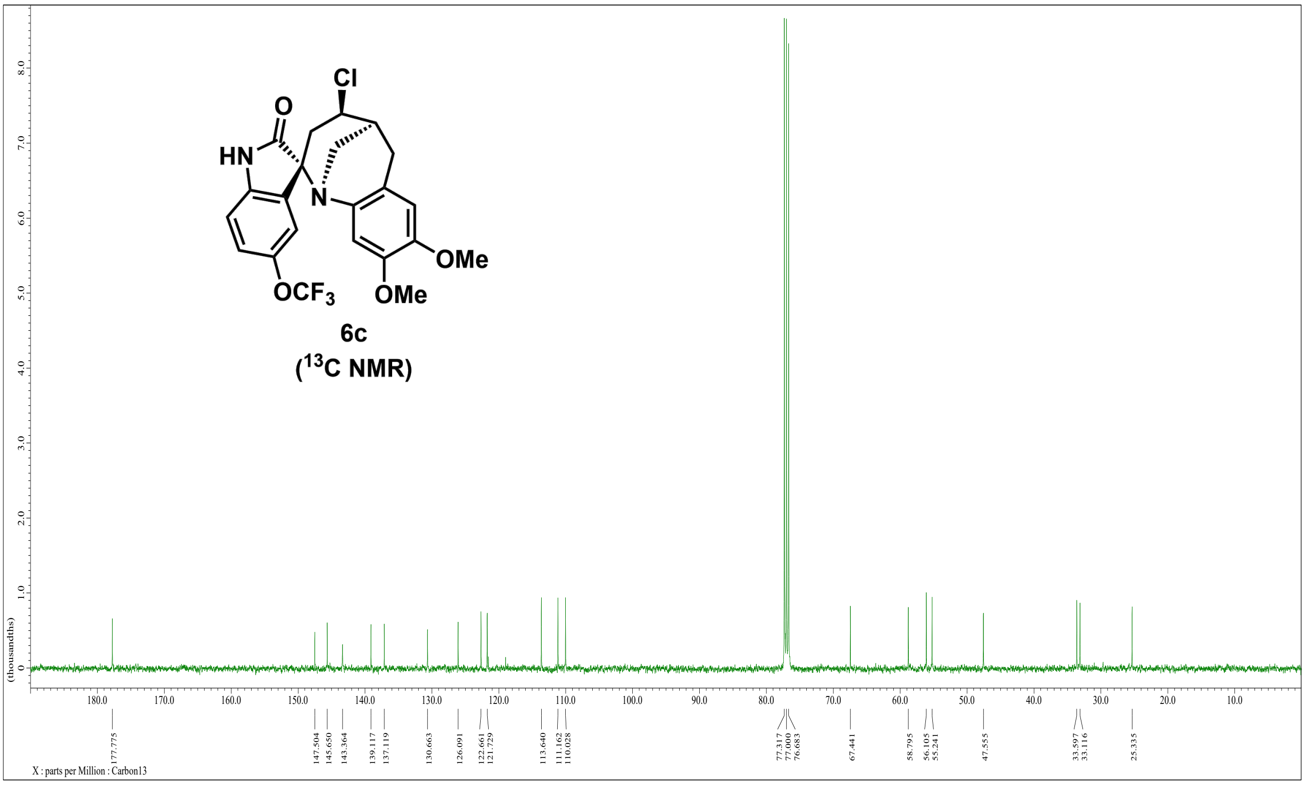

^{1}H NMR Spectrum of **6d** ($CDCl_3$, 400 MHz).

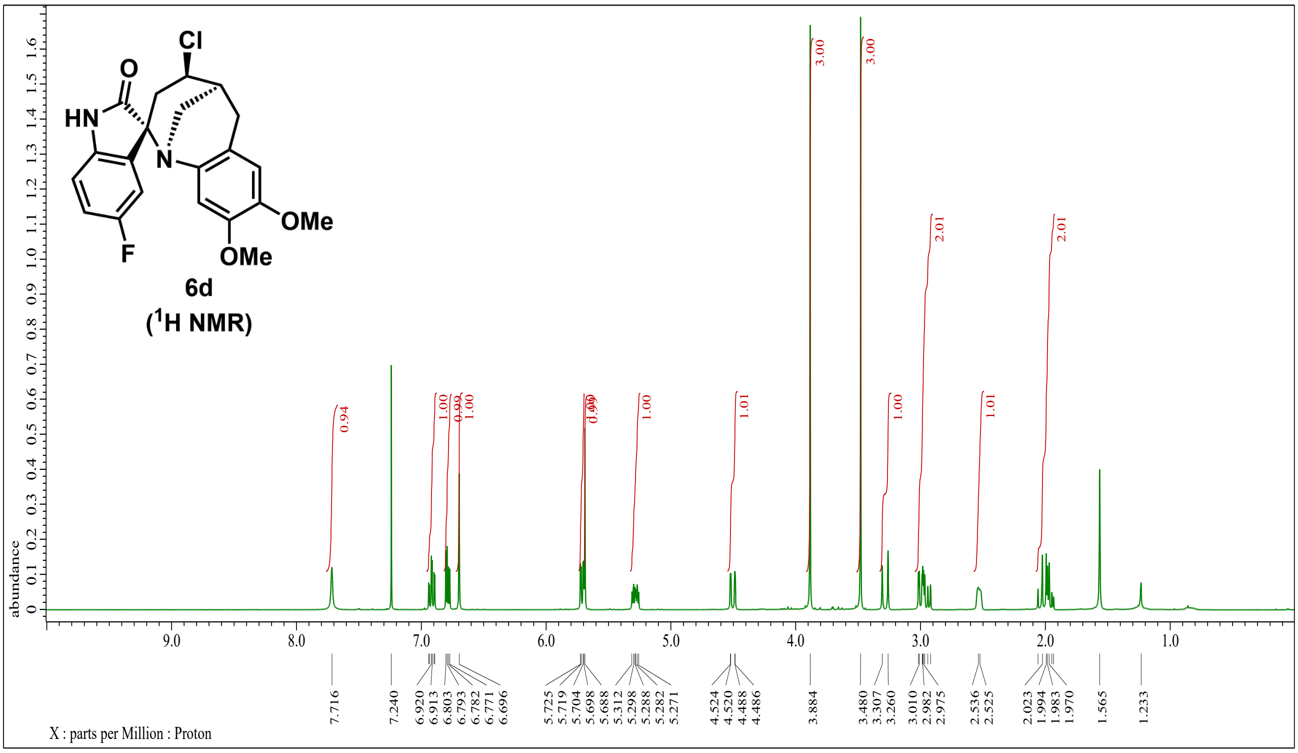

$^{13}C\{^{1}H\}$ NMR Spectrum of **6d** ($CDCl_3$, 100 MHz).

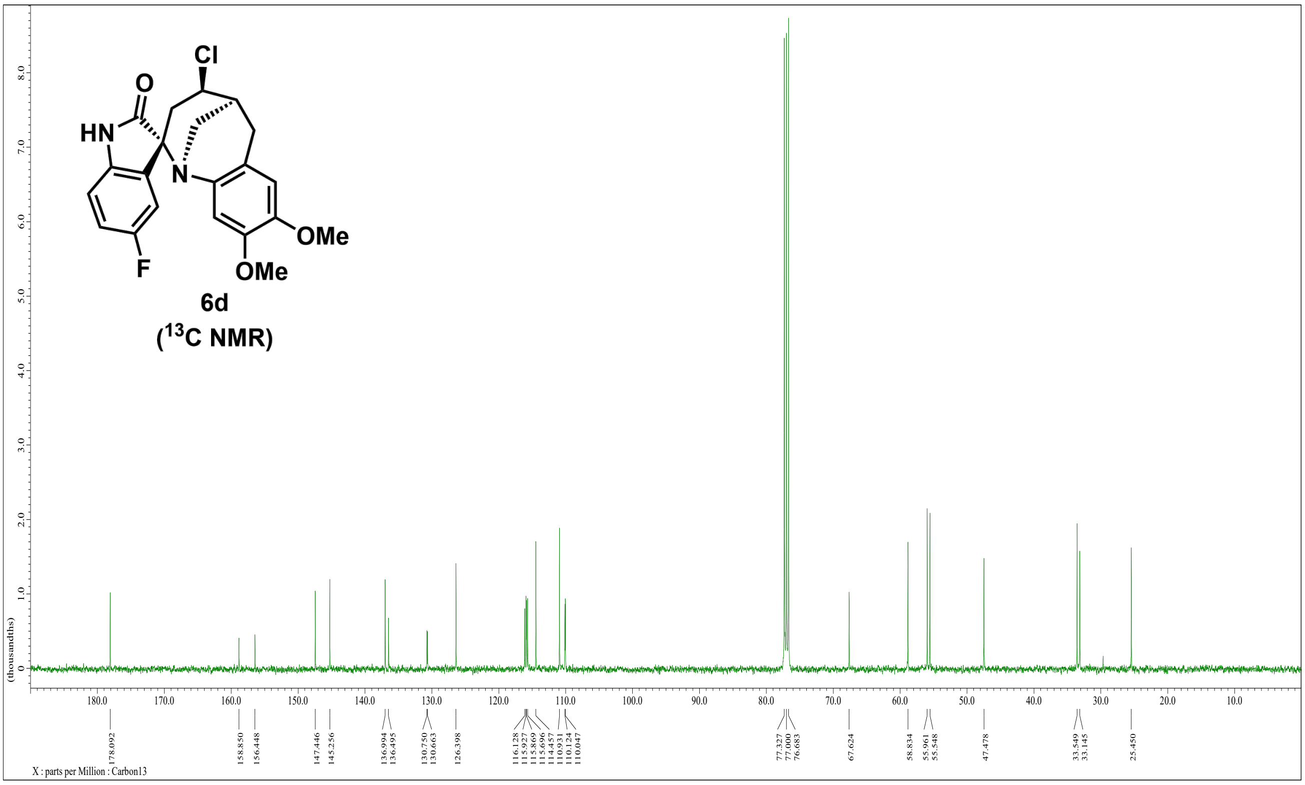

^{1}H NMR Spectrum of **6e** ($CDCl_3$, 400 MHz).

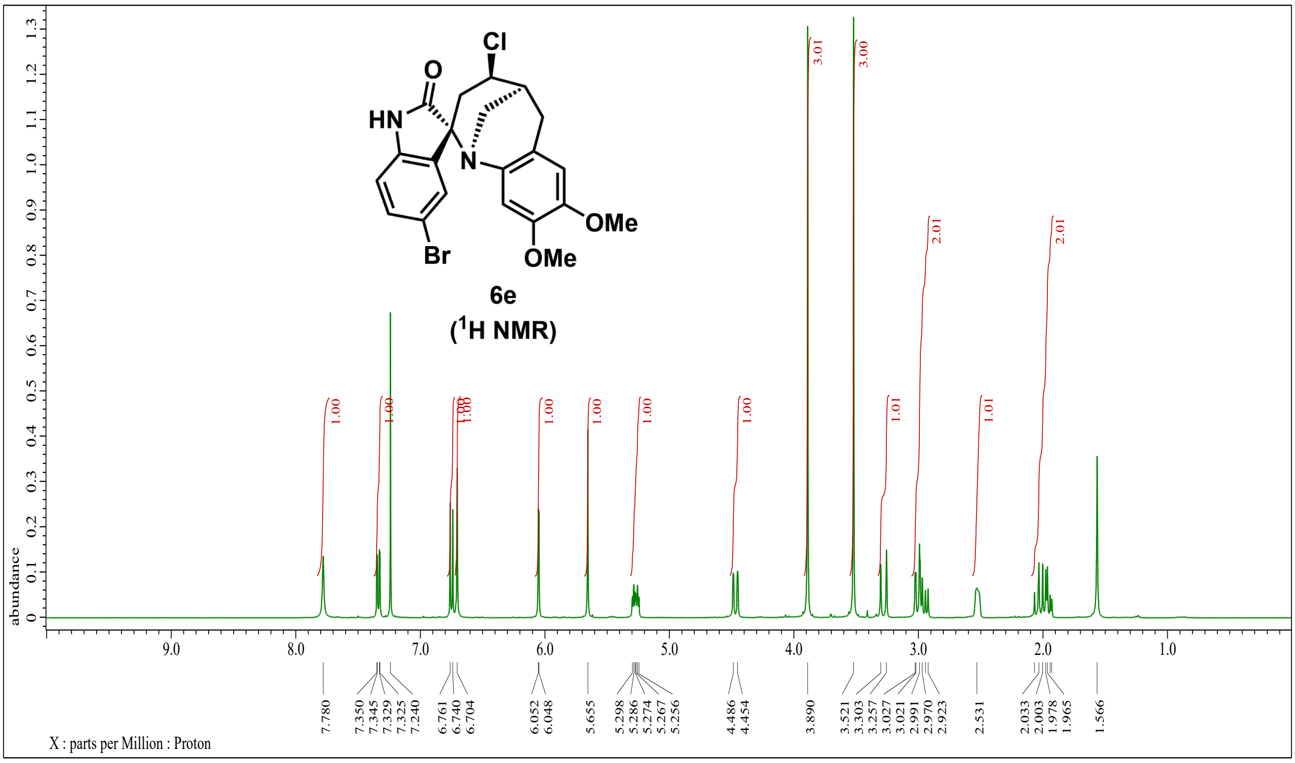

^{13}C{^{1}H} NMR Spectrum of **6e** ($CDCl_3$, 100 MHz).

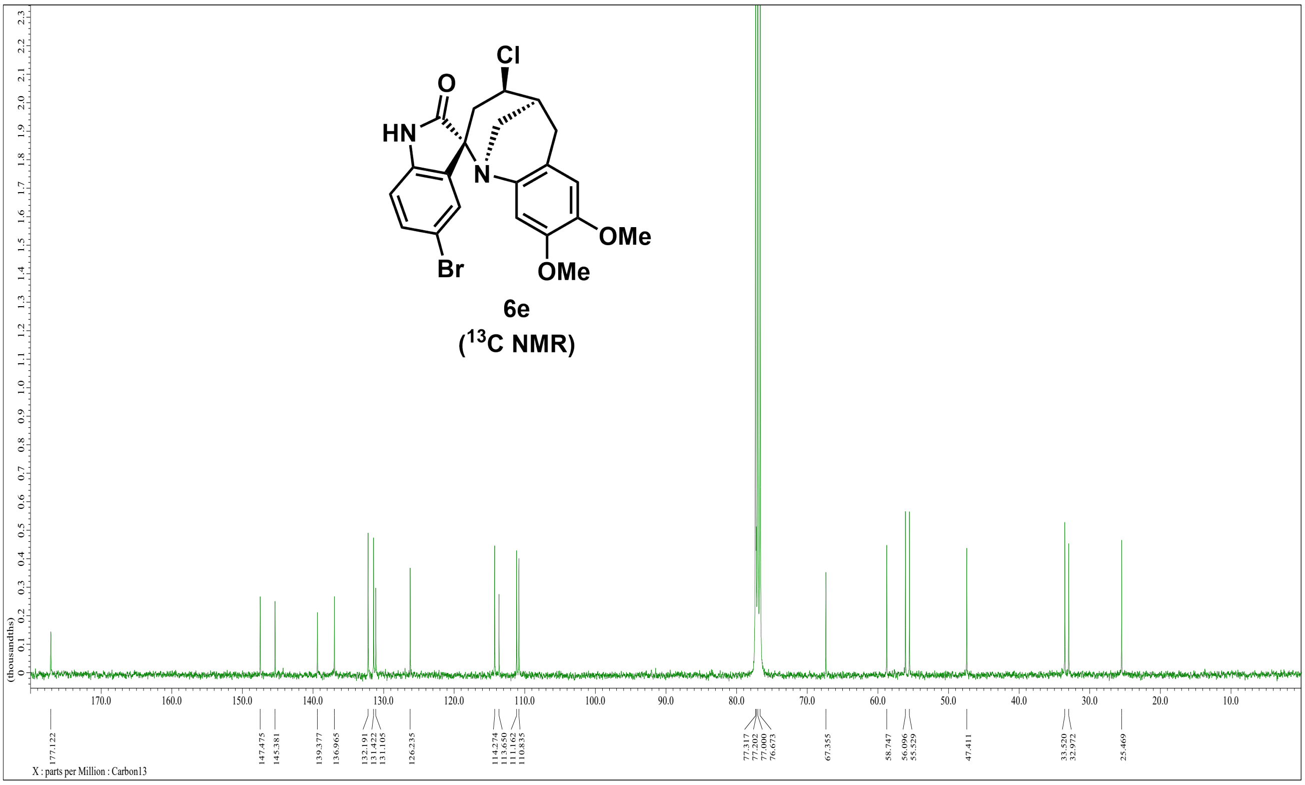

^{1}H NMR Spectrum of **6f** ($CDCl_3$, 400 MHz).

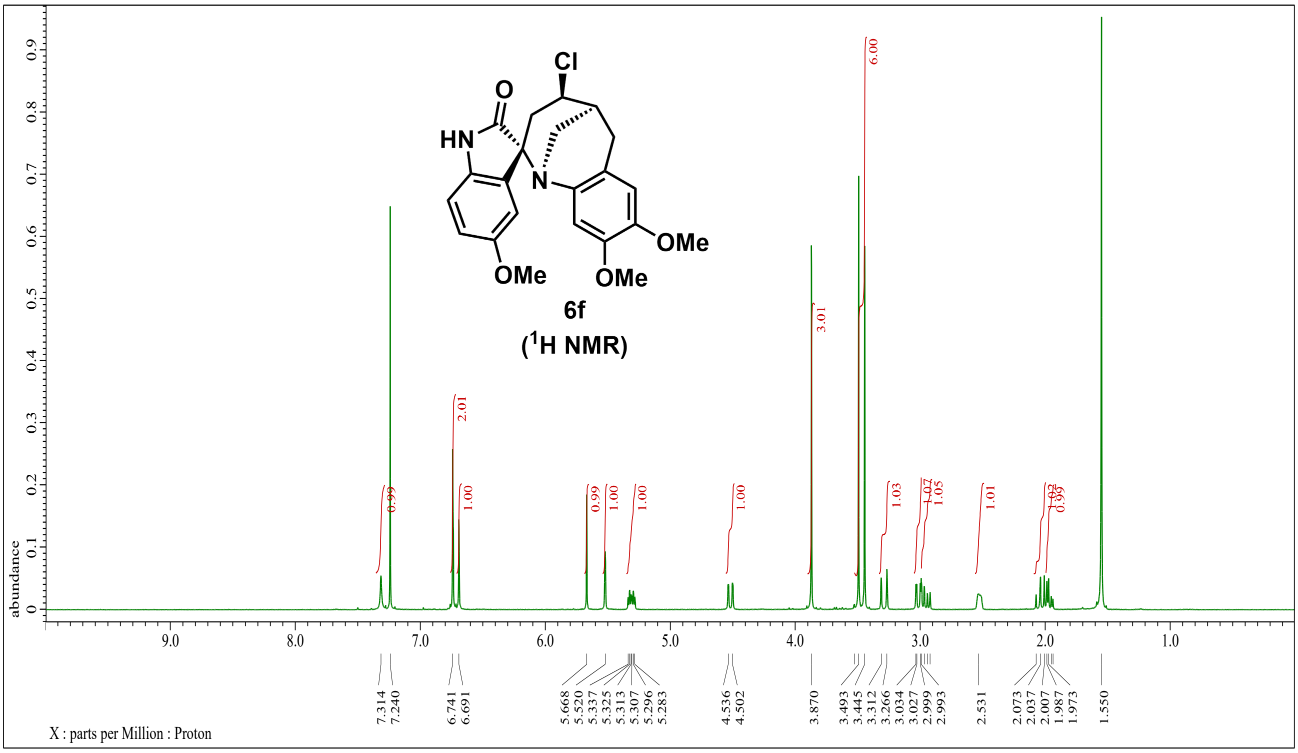

^{13}C{^{1}H} NMR Spectrum of **6f** ($CDCl_3$, 100 MHz).

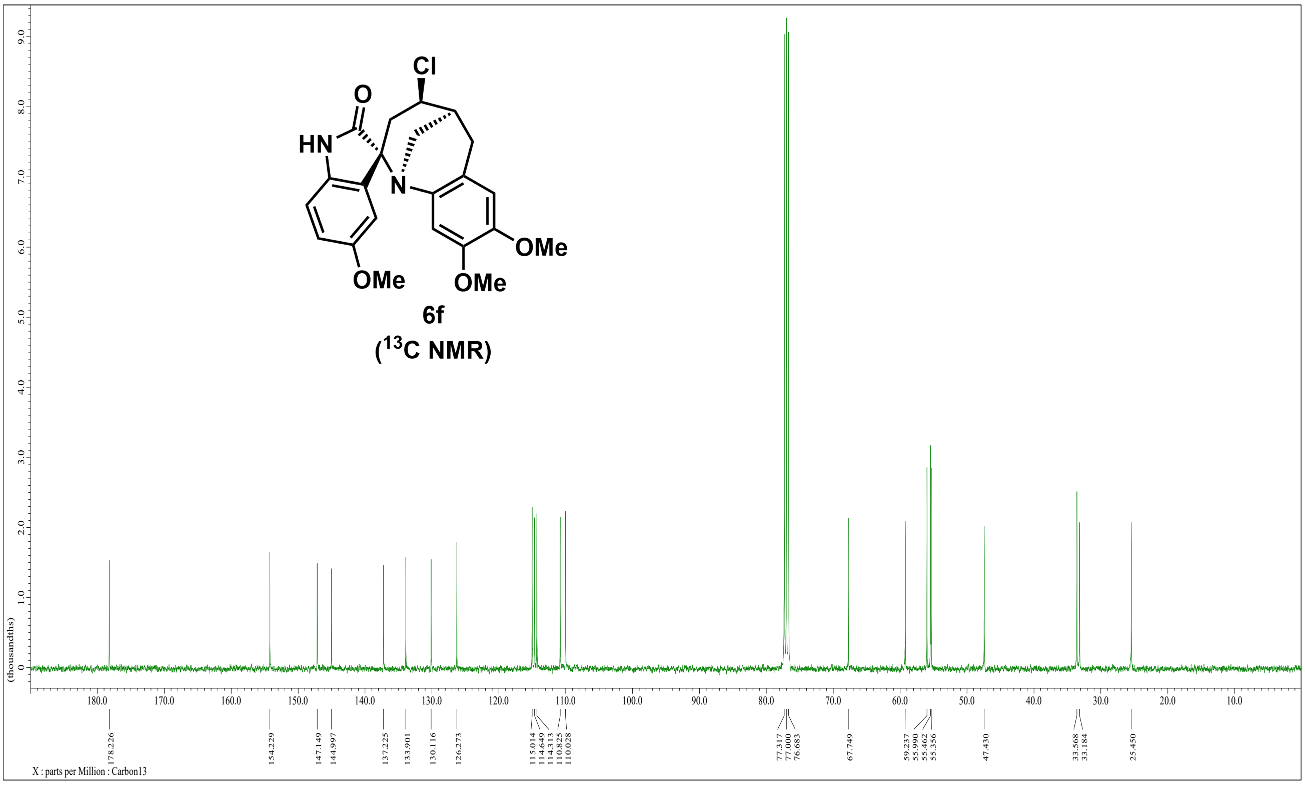

^{1}H NMR Spectrum of **6g** ($CDCl_3$, 400 MHz).

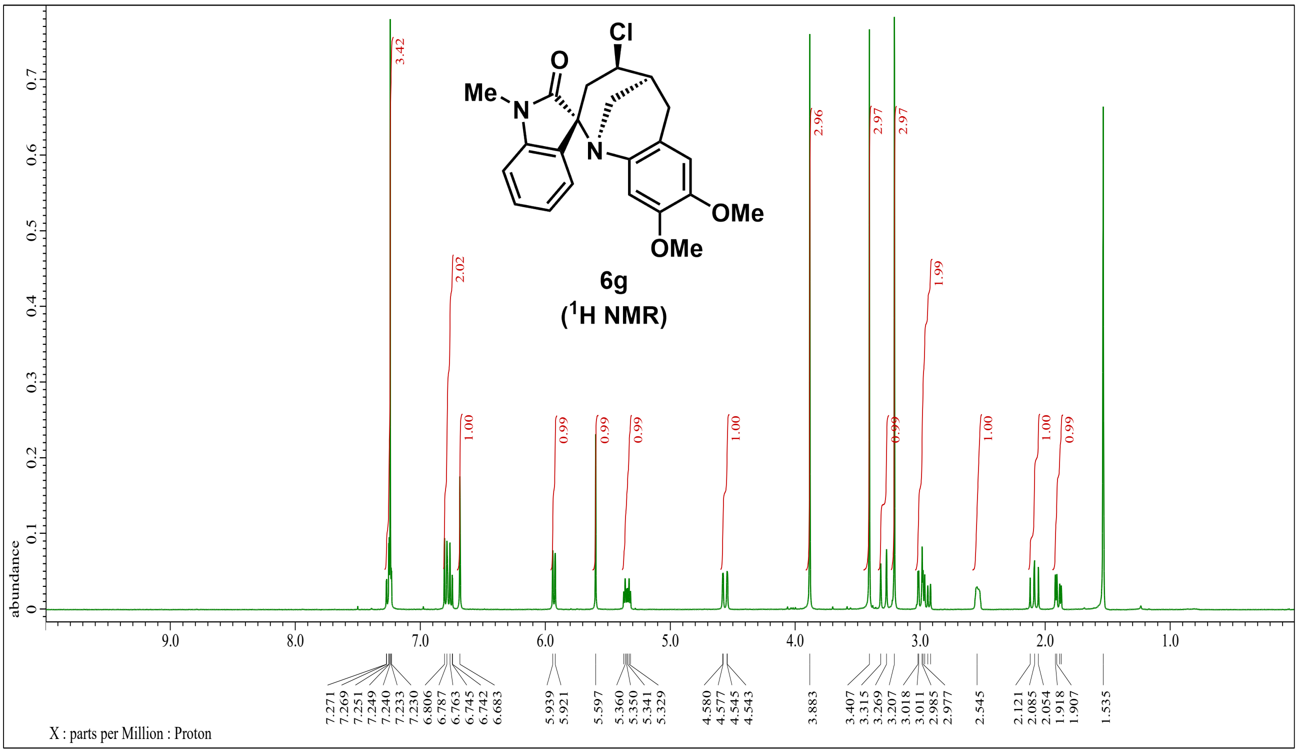

^{13}C{^{1}H} NMR Spectrum of **6g** ($CDCl_3$, 100 MHz).

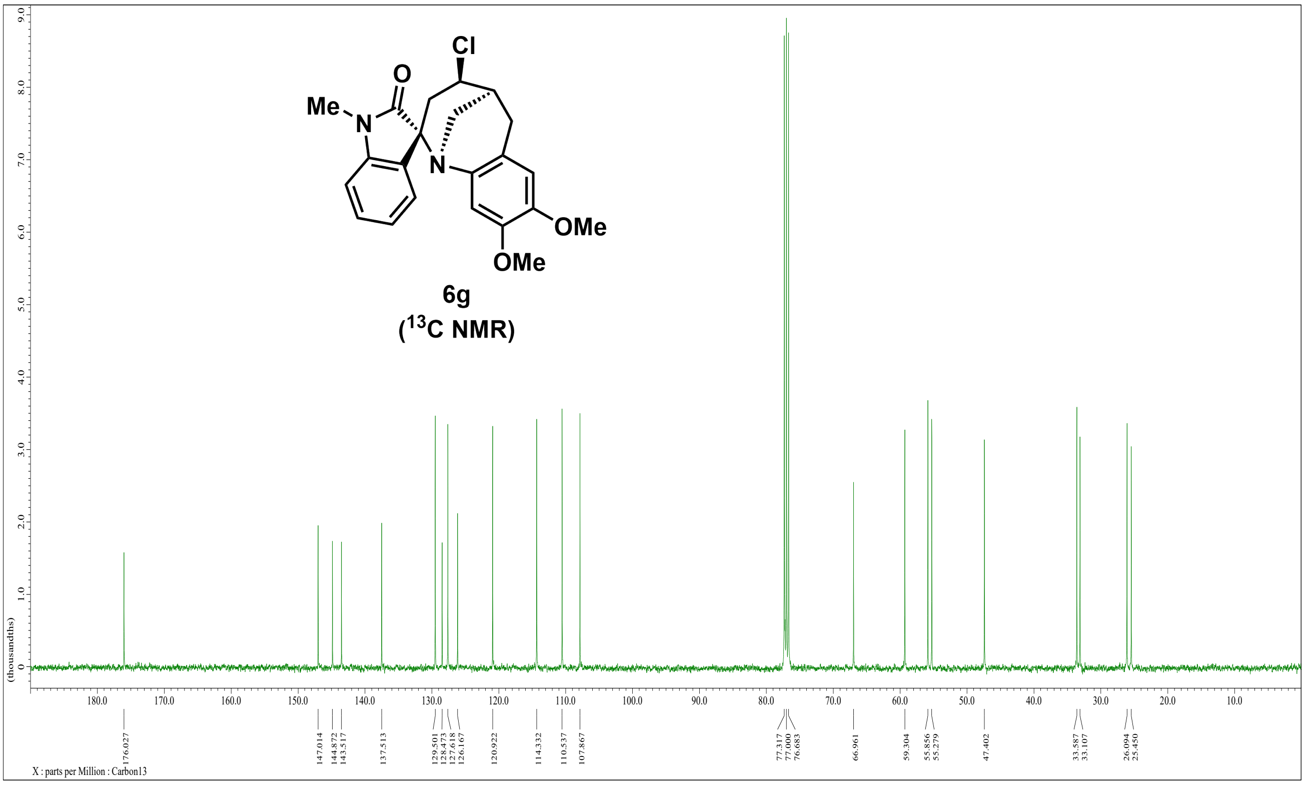

^{1}H NMR Spectrum of **6g** ($C_2D_2Cl_4$, 400 MHz)**.** This spectrum was recorded to confirm the integration of the aromatic signals.

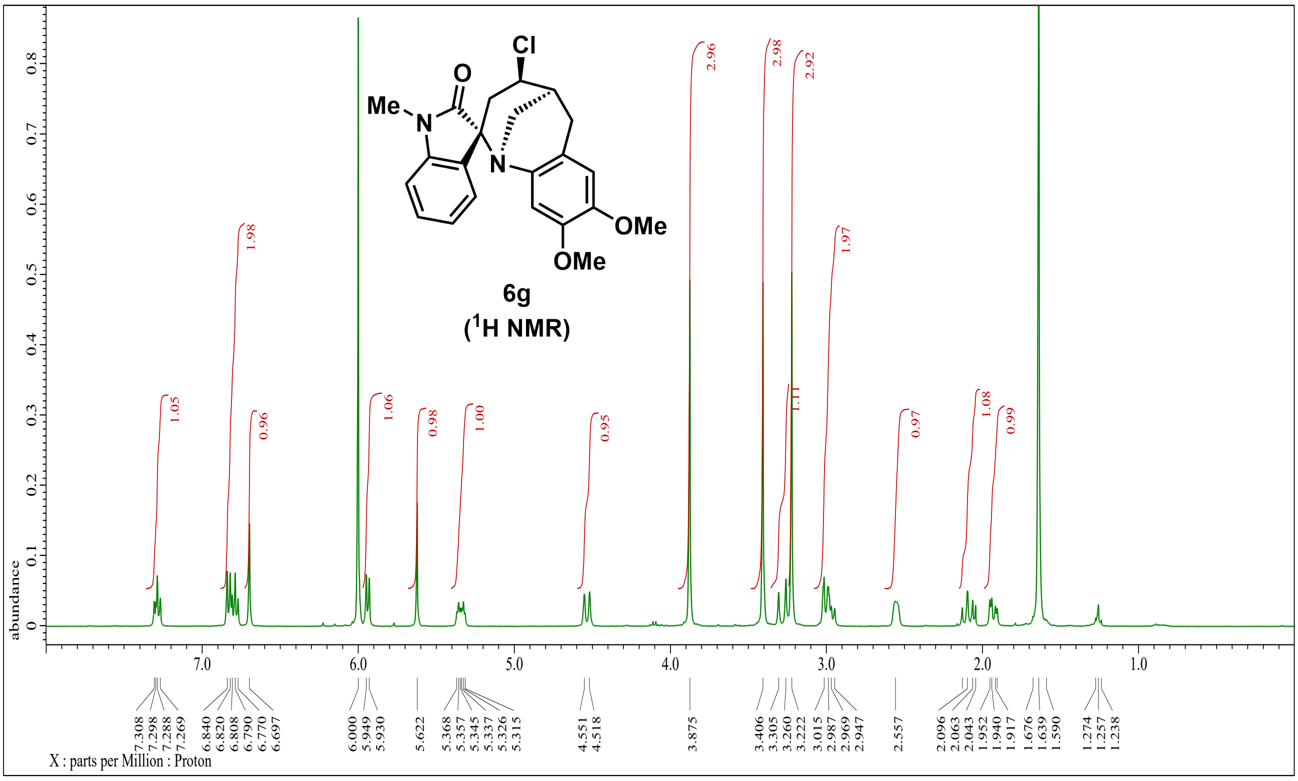

^{1}H NMR Spectrum of **6h** ($CDCl_3$, 400 MHz).

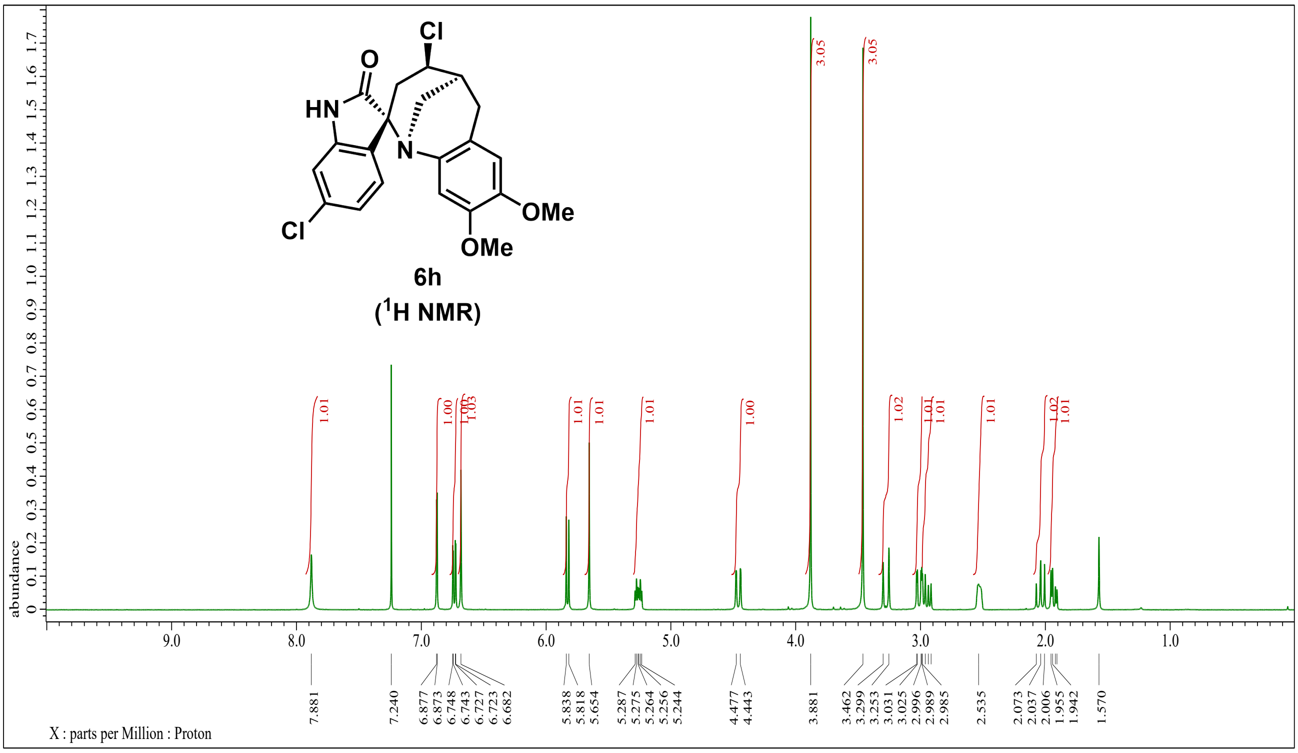

^{13}C{^{1}H} NMR Spectrum of **6h** ($CDCl_3$, 100 MHz).

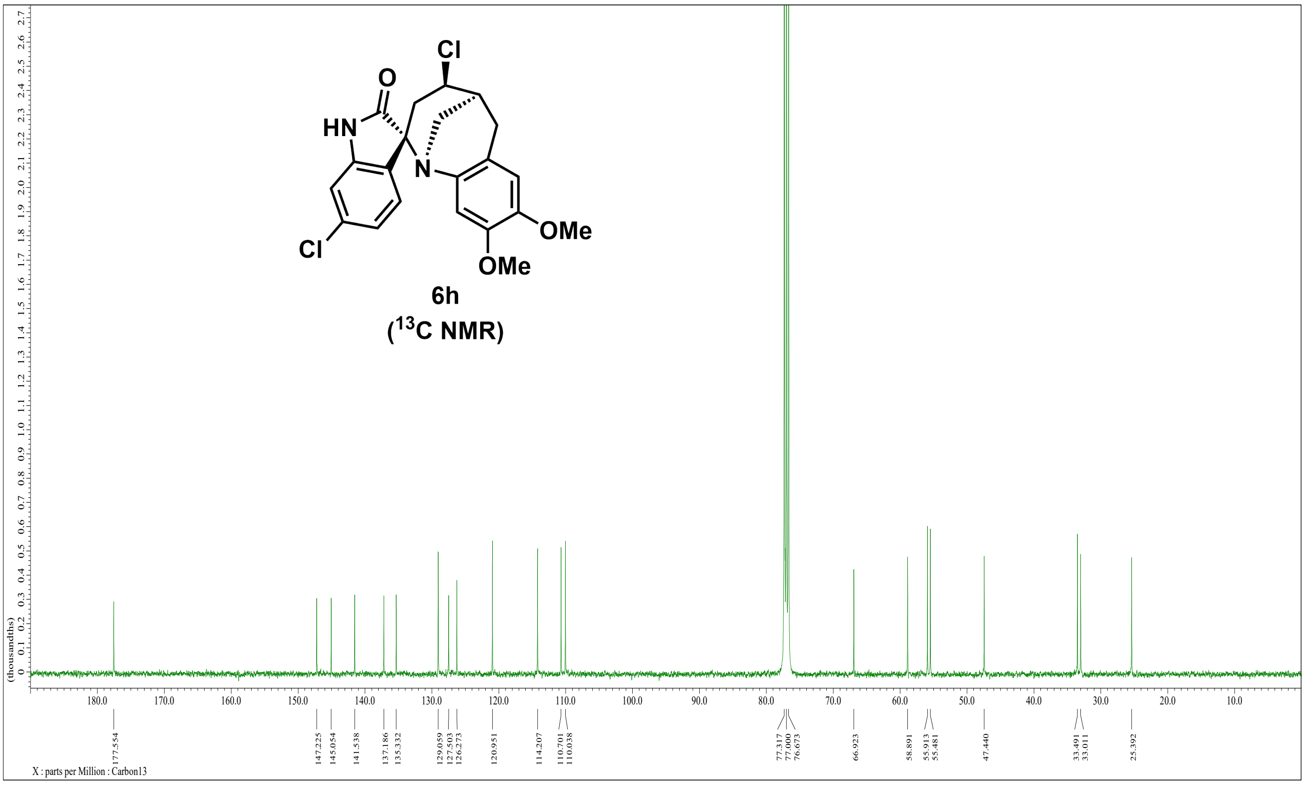

^{1}H NMR Spectrum of **6i** ($CDCl_3$, 400 MHz).

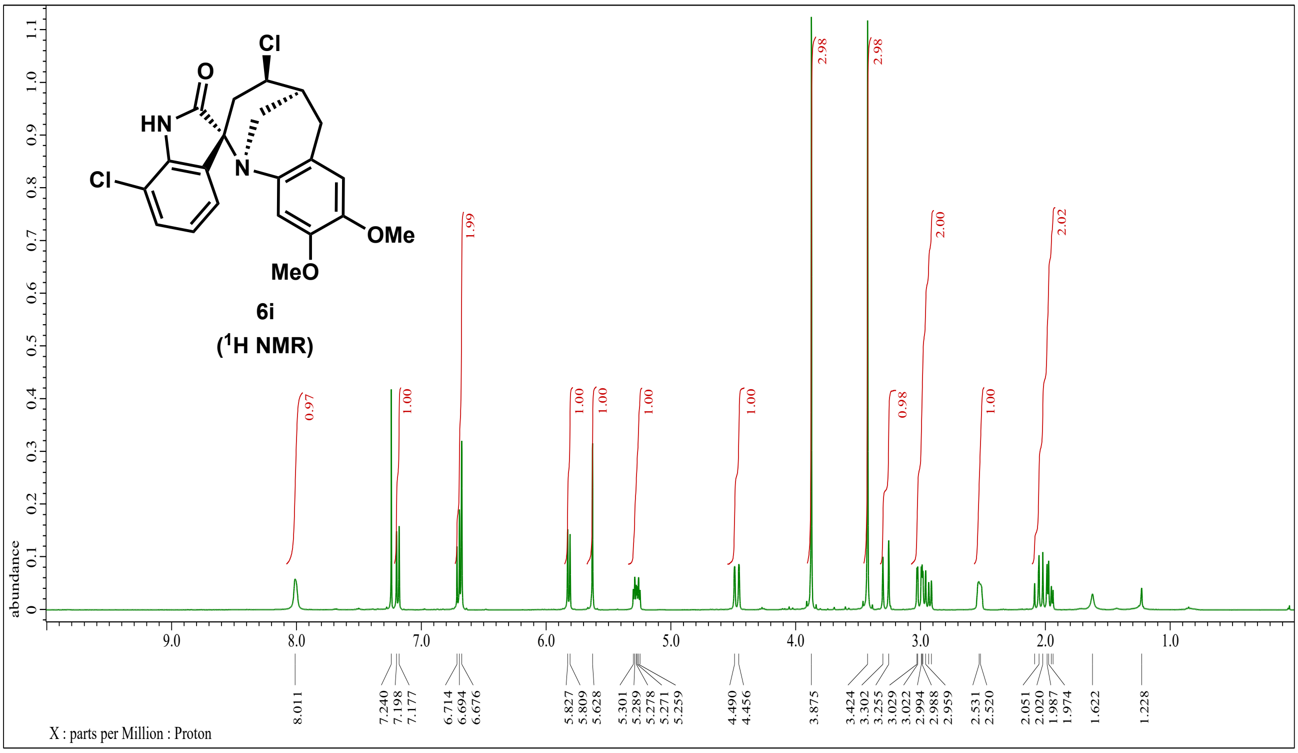

$^{13}C\{^1H\}$ NMR Spectrum of **6i** ($CDCl_3$, 100 MHz).

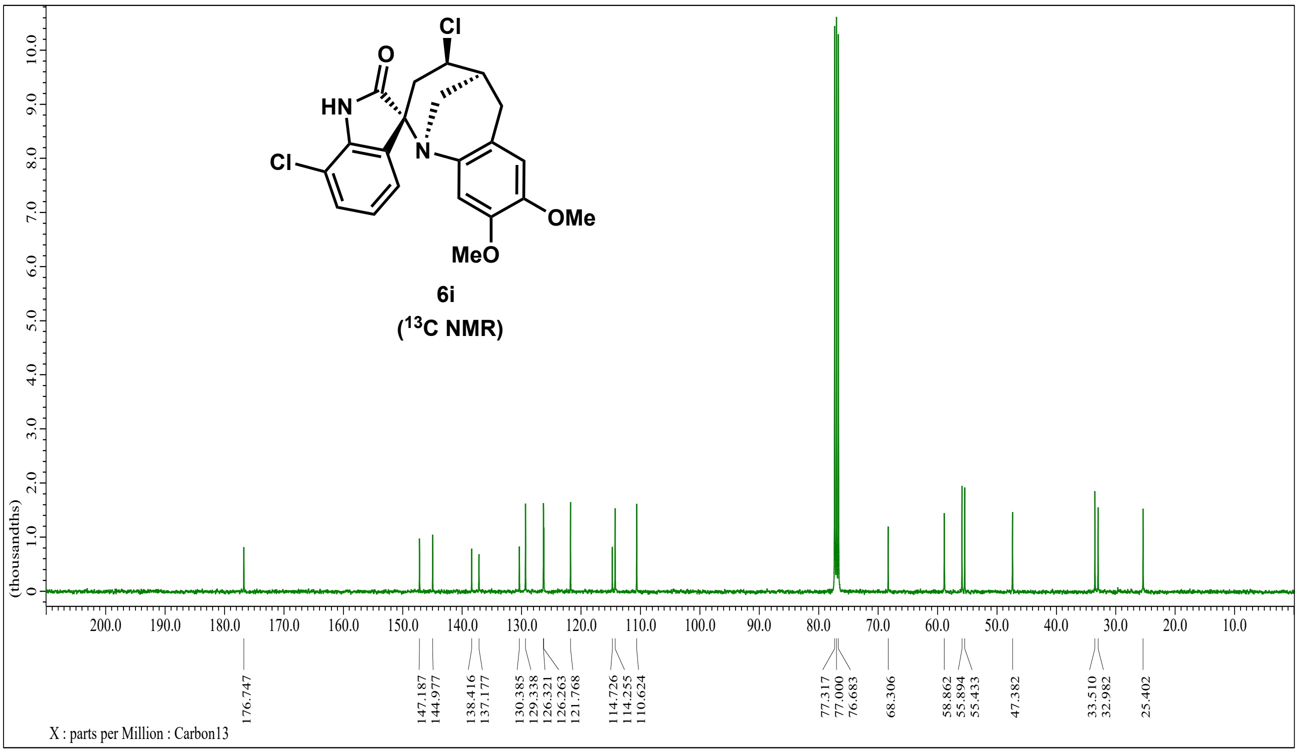

^{1}H NMR Spectrum of **6k** ($CDCl_3$, 400 MHz).

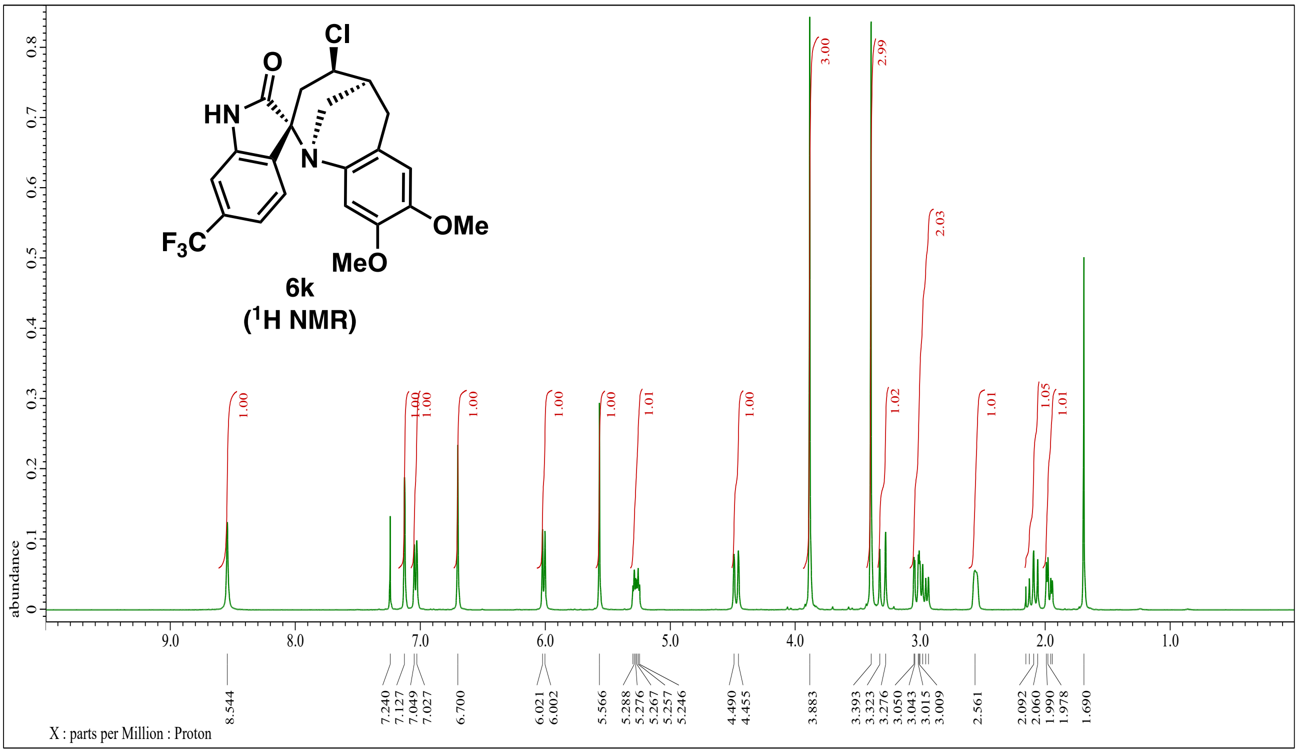

^{13}C{^{1}H} NMR Spectrum of **6k** ($CDCl_3$, 100 MHz).

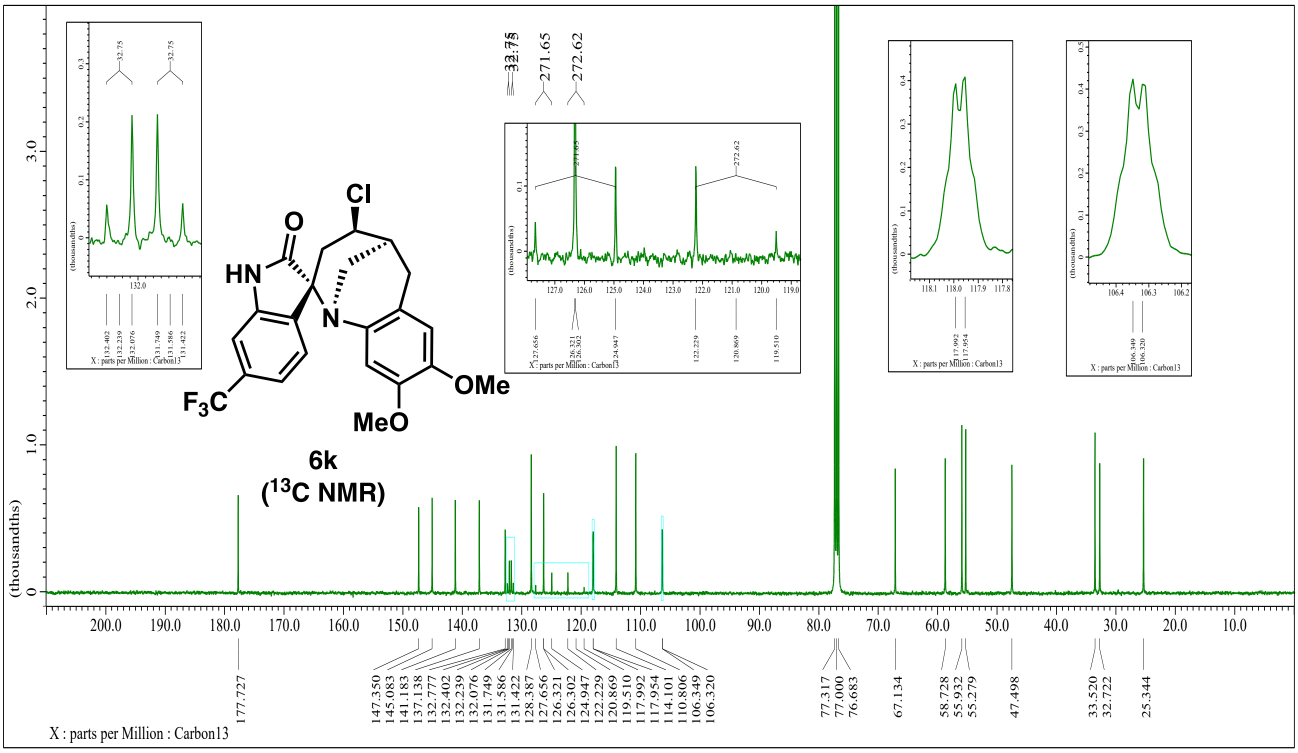

^{1}H NMR Spectrum of **6l** ($CDCl_3$, 400 MHz).

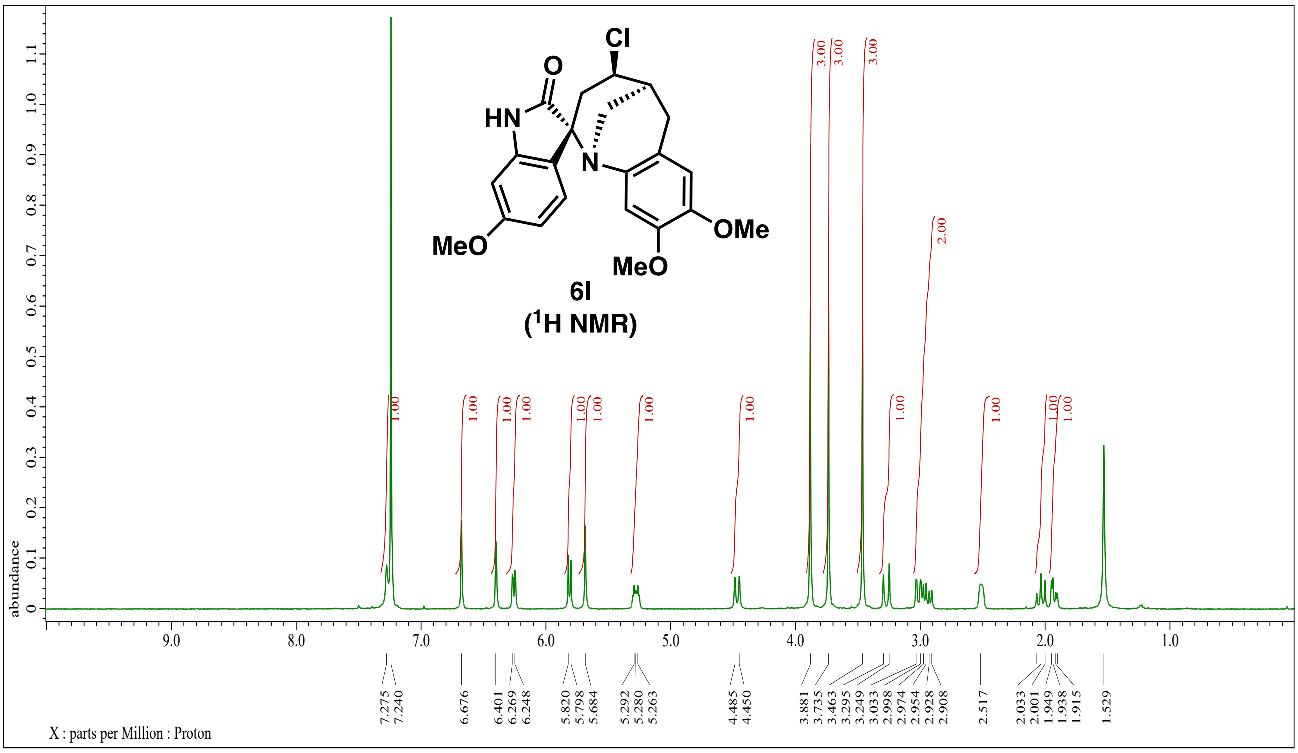

^{13}C{^{1}H} NMR Spectrum of **6l** ($CDCl_3$, 100 MHz).

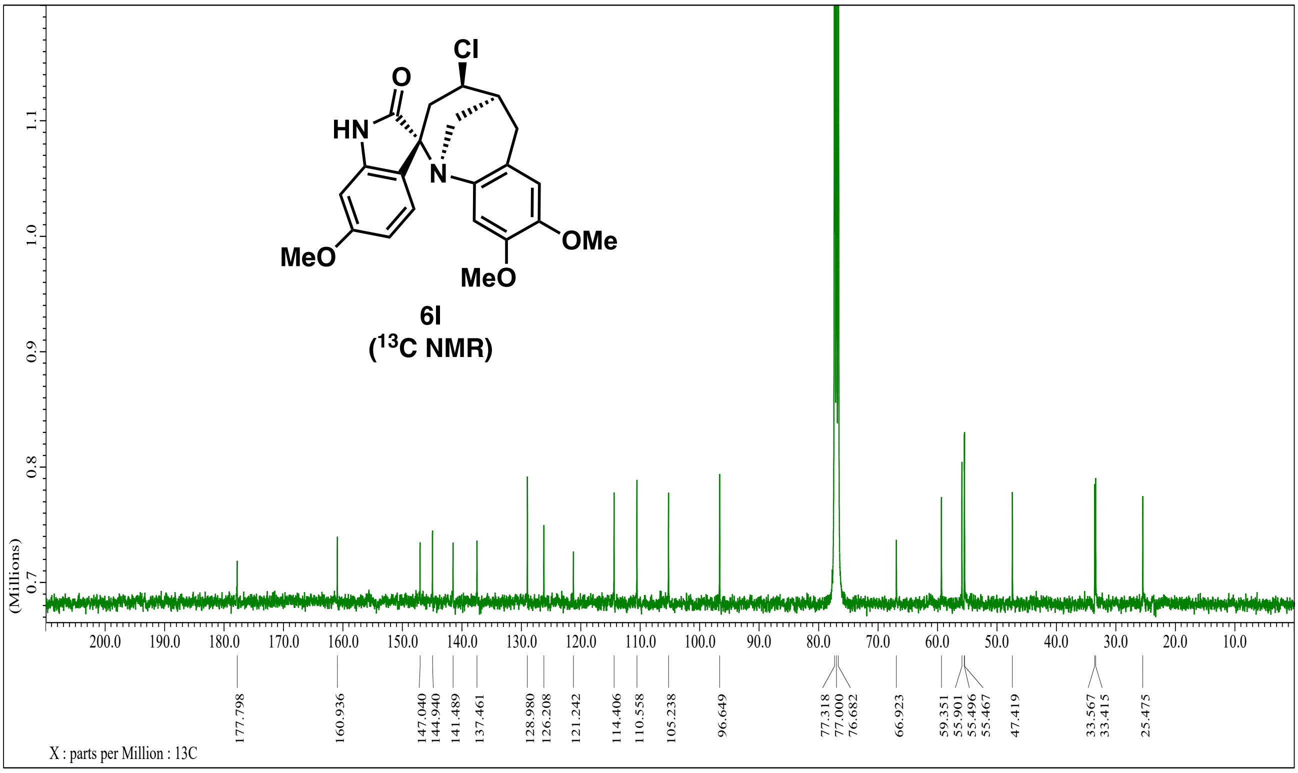

Supplement: Supplementary file 1 — jo1c01785_si_001.pdf [file jo1c01785_si_001.pdf]
